# Potentiating Activity of GmhA Inhibitors on Gram-Negative Bacteria

**DOI:** 10.1021/acs.jmedchem.4c00037

**Published:** 2024-04-10

**Authors:** François Moreau, Dmytro Atamanyuk, Markus Blaukopf, Marek Barath, Mihály Herczeg, Nuno M. Xavier, Jérôme Monbrun, Etienne Airiau, Vivien Henryon, Frédéric Leroy, Stéphanie Floquet, Damien Bonnard, Robert Szabla, Chris Brown, Murray S. Junop, Paul Kosma, Vincent Gerusz

**Affiliations:** † Mutabilis, 102 Avenue Gaston Roussel, Romainville 93230, France; ‡ Department of Chemistry, University of Natural Resources and Life Sciences, Muthgasse 18, Vienna A-1190, Austria; § Institute of Chemistry, Center for Glycomics, Slovak Academy of Sciences, Dúbravská cesta 9, Bratislava SK-845 38, Slovakia; ∥ Department of Pharmaceutical Chemistry, University of Debrecen, Debrecen 4032, Hungary; ⊥ Centro de Química Estrutural, Institute of Molecular Sciences, Faculdade de Ciências, Universidade de Lisboa, Ed. C8, 5° Piso, Campo Grande, Lisboa 1749-016, Portugal; # Activation, 10 Rue Jacquard, Chassieu 69680, France; ¶ Carbosynth Limited, 8&9 Old Station Business Park, Compton, Berkshire RG20 6NE, U.K.; ∇ Department of Biochemistry, University of Western Ontario, London, ON N6A 3K7, Canada

## Abstract

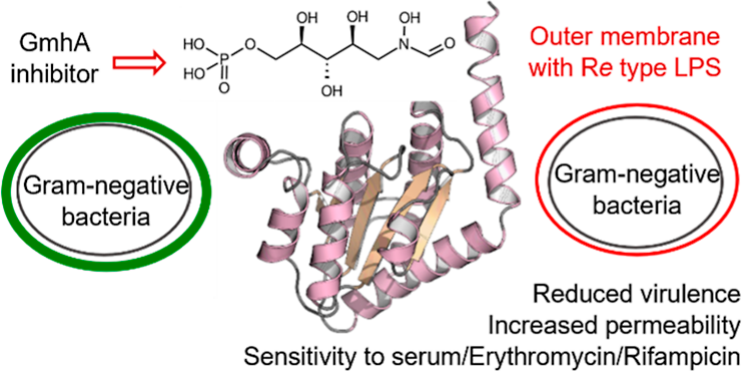

Inhibition of the biosynthesis of bacterial heptoses opens novel perspectives for antimicrobial therapies. The enzyme GmhA responsible for the first committed biosynthetic step catalyzes the conversion of sedoheptulose 7-phosphate into d-*glycero*-d-*manno*-heptose 7-phosphate and harbors a Zn^2+^ ion in the active site. A series of phosphoryl- and phosphonyl-substituted derivatives featuring a hydroxamate moiety were designed and prepared from suitably protected ribose or hexose derivatives. High-resolution crystal structures of GmhA complexed to two *N*-formyl hydroxamate inhibitors confirmed the binding interactions to a central Zn^2+^ ion coordination site. Some of these compounds were found to be nanomolar inhibitors of GmhA. While devoid of HepG2 cytotoxicity and antibacterial activity of their own, they demonstrated in vitro lipopolysaccharide heptosylation inhibition in *Enterobacteriaceae* as well as the potentiation of erythromycin and rifampicin in a wild-type *Escherichia coli* strain. These inhibitors pave the way for a novel treatment of Gram-negative infections.

## Introduction

Infections caused by Gram-negative pathogens are presently of significant medical importance due to increasing bacterial resistance with life-threatening consequences for patients.^1^ Development of new antimicrobial drugs in a forthcoming post-antibiotic era is thus an urgent task to be accomplished by joint efforts of the pharmaceutical industry and academia. In particular, novel modes of action need to be elaborated to overcome the rapid adaptation of many bacteria to conventional antibiotics.

The bacterial lipopolysaccharide (LPS) is located in the outer leaflet of the outer membrane of Gram-negative bacteria and exerts a barrier function against many antibacterial agents. LPS is an amphipathic molecule consisting of the endotoxically active lipid A, a core oligosaccharide domain, and for some Gram-negative bacteria the surface exposed polysaccharide O-antigen.^2^ The inner core of LPS is made of 2 to 3 phosphorylated heptoses. These residues together with divalent cations are responsible for the stability of the outer leaflet and for its gatekeeper role against lipophilic compounds. As such, they constitute a key bottleneck for the penetration of antibacterials in the cytosol of Gram-negative bacteria. Bacterial knockout mutants being devoid of the heptose region of their LPS (termed deep-rough phenotype) show reduced virulence and biofilm formation, are sensitive to serum complement, and display a more permeable outer membrane allowing potentiation of antibiotics such as macrolides or rifamycins.^3^ Thus, inhibition of the biosynthetic pathways leading to the incorporation of heptoses in LPS and bacterial glycoproteins is regarded as a valuable option to develop antivirulence and potentiating therapies.^4^

**Scheme 1:**
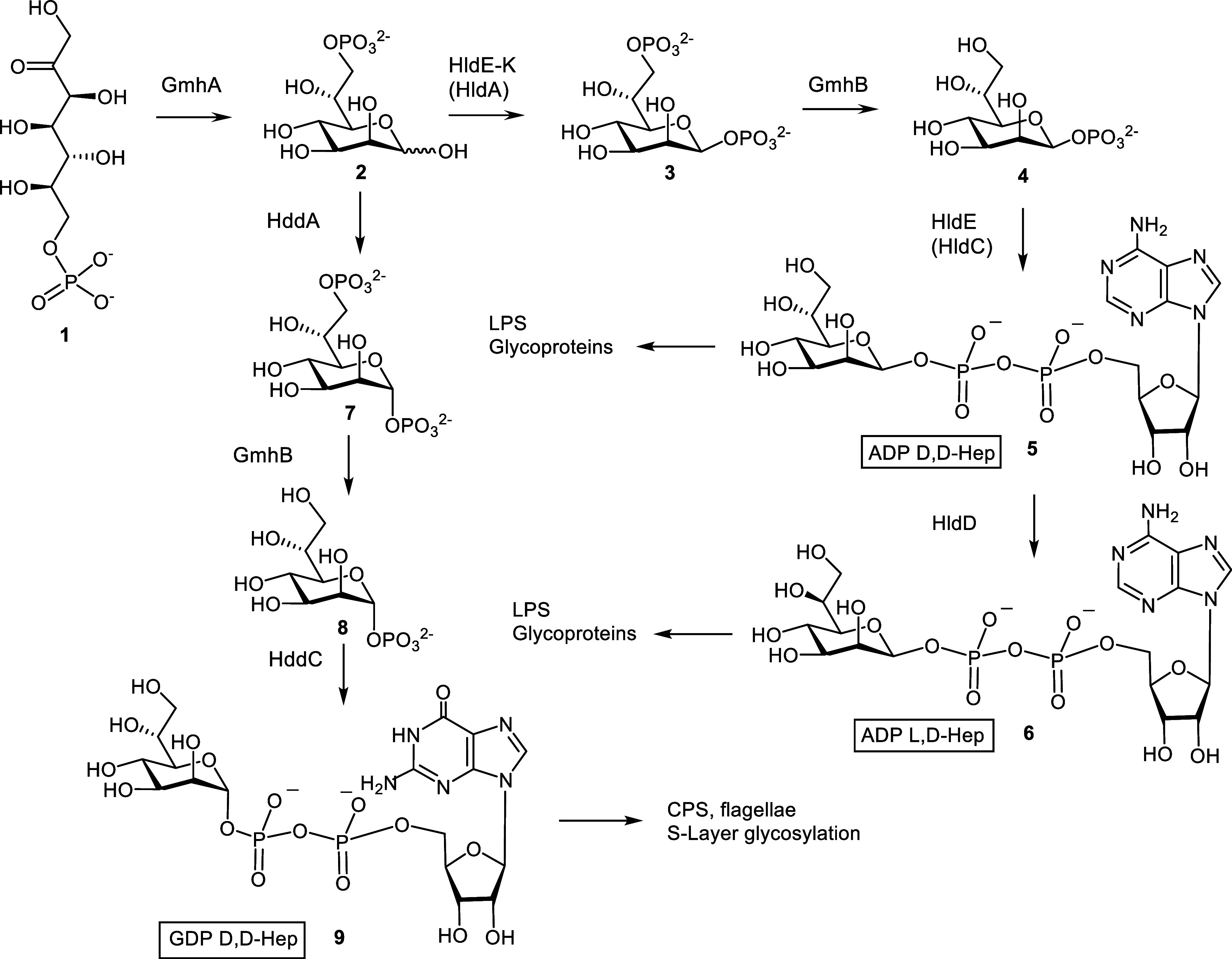
Biosynthetic Pathways toward Nucleotide Activated Heptoses That Would Be Affected by Inhibiting the GmhA Reaction

The biosynthesis of these bacterial heptoses has been elucidated in the past decades and starts from the conversion of sedoheptulose 7-phosphate **1** into d-*glycero*-d-*manno*-heptose 7-phosphate **2** by the action of the ketose-aldose isomerase GmhA (Scheme 1).^5^

Next, the pathway diverges into two branches, eventually leading to the formation of ADP d-*glycero*- (**5**) and l-*glycero*-β-d-*manno*-heptose (**6**) involved in the assembly of bacterial LPS and alternatively produces GDP d-*glycero*-α-d-*manno*-heptose (**9**) needed for the biosynthesis of heptose constituents in capsular polysaccharides as well as for S-layer glycoproteins.^6^ As the first ketose to aldose isomerization step is common to the biosynthesis of all of these bacterial heptoses, inhibition of the sedoheptulose 7-phosphate isomerase reaction constitutes an attractive target to generate antimicrobial activities. This enzyme also called GmhA has been biochemically and structurally well characterized.^7^ Crystal structures of apo-forms have been reported for *Escherichia coli*,^7^*Colwellia psychrerythraea,* and *Neisseria gonorrhoeae*,^8^ as well as in liganded form with heptose 7-phosphate **2** for *Burkholderia pseudomallei*(9) and *Pseudomonas aeruginosa*.^7^ In addition, liganded structures containing sedoheptulose-7-phosphate **1** from *E. coli*, *B. pseudomallei,* and *Mycobacterium tuberculosis* have also been published.^10^ GmhA is well conserved among many Gram-negative bacteria but also some Gram-positive bacteria and shares little similarity with human enzymes, which reinforces its interest as a potential drug target.^6a^ Previously, several small molecules have been generated as inhibitors of GmhA and the downstream kinases and transferases.^11^ The most potent GmhA inhibitor was identified as methyl 7-*O*-phosphoryl-d-*glycero*-d-*gluco*-heptopyranoside, a substrate analogue, with IC_50_ values in the low micromolar range.^12^ It was yet still too high to demonstrate heptosylation inhibition in a wild-type *E. coli* strain at 1 mM. In 2010, Harmer reported the presence of a zinc atom in *B. pseudomallei* GmhA as a key feature contributing to its catalytically active conformation.^13^ The presence of a metal at the heart of the active site was also postulated to be a general motif across a wide-spectrum of Gram-negative and Gram-positive bacterial GmhA as inferred from the excellent conservation of the zinc-binding side chains. The addition to substrate analogues of an hydroxamic acid moiety that is able to chelate zinc ions^14^ and therefore improve their potency would constitute a logical exploration strategy.

However, this strategy would make sense only if the designed GmhA inhibitors could address the well-known permeation and efflux challenge in Gram-negative bacteria and efficiently reach their target in the cytosol. Heptose analogues are small hydrophilic compounds that are expected to permeate easily into the periplasm through the outer membrane via the porins. Then, crossing the lipophilic inner membrane to reach the cytosol requires a priori an active transport mechanism. From the many transporter candidates for sugar import,^15^ the hexose-phosphate transporter UhpT would be considered the most plausible one as it can import a variety of phospho-sugars like pentose, hexose, and heptose phosphates but also phosphonate derivatives like fosfomycin and fosmidomycin. Importantly, the GmhA substrate sedoheptulose 7-phosphate **1** as well as the GmhA product d-*glycero*-d-*manno*-heptose 7-phosphate **2** are also transported by UhpT (Figure S3).

Based on our synthetic experience of heptose derivatives,^16^ we therefore decided to explore GmhA inhibition with phospho- or phosphono-sugars substituted with an hydroxamic acid moiety, postulated to reach their cytosolic target via porins and UhpT with the added advantage of excellent compound solubility. Herein, we describe the synthesis and structure–activity relationship of this novel series of GmhA inhibitors and two crystal structures of inhibitors liganded to GmhA from *B. pseudomallei*. We also report for the first time inhibition of LPS heptosylation on wild-type *Enterobacteriaceae*, leading to potentiation of macrolide and rifamycin antibiotics against Gram-negative pathogens.

## Chemical Syntheses

**Scheme 2:**
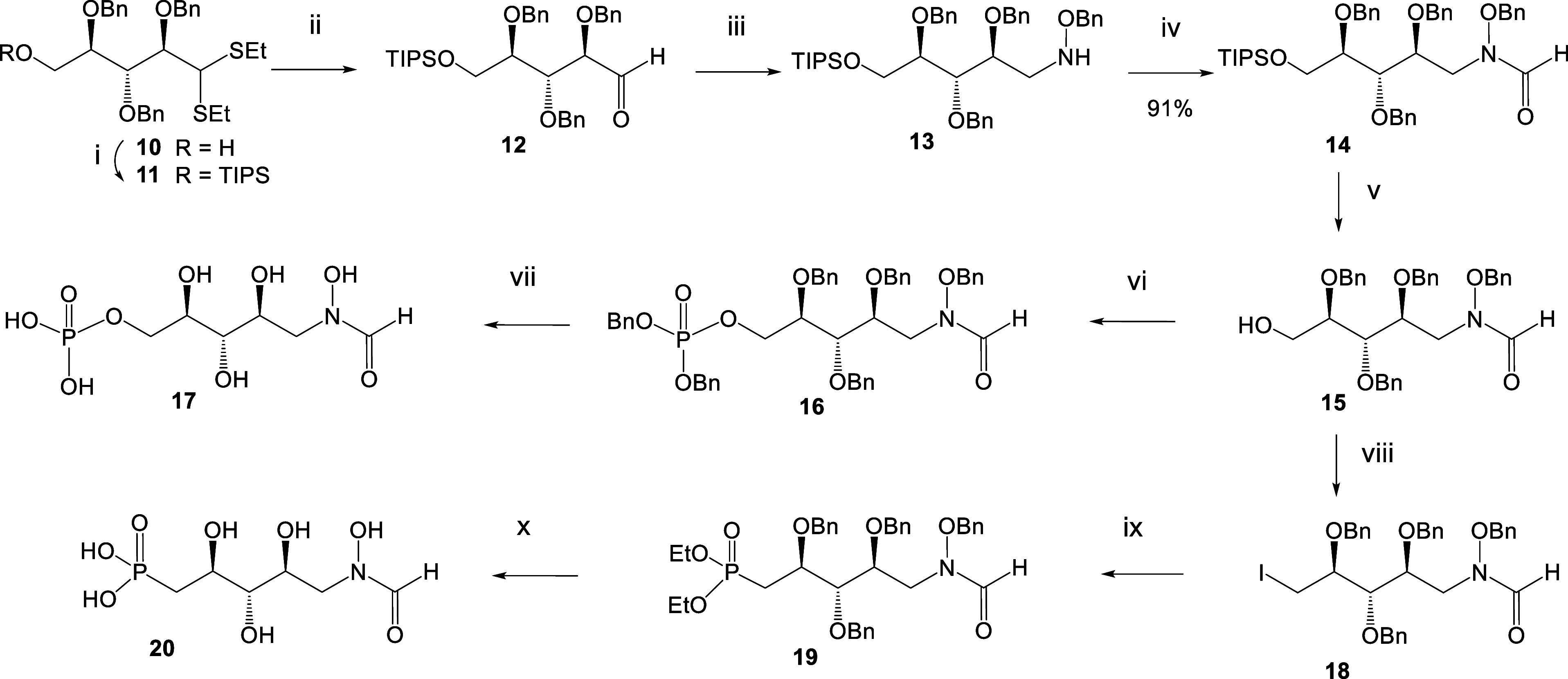
Synthesis of the *N*-formyl-*N*-hydroxy Inhibitors **17** and **20** Reagents and Conditions: (i) TIPSCl, DMAP, pyr, 93%; (ii) NBS, aq. Me_2_CO, 97%; (iii) 1. H_2_NOBn, pyr., MeOH 2. NaCNBH_3_, AcOH, 75% over two steps; (iv) CDI, HCOOH, THF, 0 °C, 94%; (v) TBAF, THF, 87%; (vi) 1. 1*H*-tetrazole, phosphoramidite, rt. 2. *m*CPBA, TEA, −78 °C, 93%; (vii) Pd/C, H_2_, aq. THF-HOAc rt, 30%; (viii) I_2_, Imidazole, P(Ph)_3_, 66%; (ix) P(OEt)_3_, 44%; (x) 1. TMSBr, pyr. 2. Pd(OH)_2_/C, H_2_, aq. THF, 1% AcOH, 23%.

For the synthesis of the *N*-hydroxyformate derivatives **17** and **20**, the known 2,3,4-tri-*O*-benzyl ribose dithioacetal **10**(17) was converted into the 7-*O*-triisopropylsilyl derivative **11** using triisopropylsilyl chloride in pyridine in the presence of *N,N*-dimethylaminopyridine. Next, the aldehyde was liberated by the action of *N*-bromosuccinimide in aqueous acetone to afford compound **12** (Scheme 2). Reductive amination of **12** was achieved first by treatment with benzyloxyamine in pyridine/MeOH, isolation of the crude oxime by column chromatography followed by reduction with sodium cyanoborohydride in acetic acid to give the benzyloxyamine derivative **13**.^18^*N*-formylation was carried out by reaction with carbonyldiimidazole in formic acid/THF to produce compound **14**. NMR-analysis of the tertiary amide was complicated by the occurrence of rotamers.^19^ In order to assign broadened ^1^H and ^13^C NMR signals, measurements had to be performed in deuterated toluene at 80 °C. In contrast, measurements performed at 40 °C resulted in two well separated spin systems. Removal of the silyl ether was smoothly accomplished in the presence of TBAF in THF to furnish primary alcohol **15**. Subsequent phosphorylation of **15** was achieved by reaction with dibenzyl *N,N*-diisopropylphosphoramidite in the presence of 1*H*-tetrazole at room temperature, followed by oxidation with *m*CPBA at −78 °C.^20^ Phosphotriester **16** was isolated by HPLC purification, and catalytic hydrogenation of **16** in the presence of Pd-carbon using a solvent mixture of aqueous THF-HOAc followed by purification on a HILIC column afforded the target monophosphate **17**. The modest yield was due to significant byproduct formation, in particular during longer reaction times, giving rise to deformylation as well as N–O reduction of the hydroxylamino group. Progress of the deprotection had thus to be checked by HILIC separation of aliquots using an analytical HILIC column and several cycles of HILIC runs had to be performed with concomitant purity assessment by NMR analysis prior to pooling of the individual fractions (see Figure S1, Supporting Information). Similar to the protected *N*-formyl derivatives **14**–**16**, the deprotected derivative **17** was also present as a rotameric mixture, leading to two proton signals of the formyl unit at 8.35 and 7.90 ppm, respectively. To prove the dynamic exchange between the two rotamers, 1D gradient NOE-difference spectra according to Ley^19^ were measured. Irradiation of a solution of **17** at 7.90 ppm also led to a negative signal at the second formyl proton thus confirming that both protons are engaged in a chemical exchange equilibrium (Figure S2, Supporting Information).

For the synthesis of the shortened *C*-phosphonate analogue **20**, alcohol **15** was treated with triphenyl phosphine, imidazole, and iodine to afford the deoxy-iodo derivative **18**. The structure of the iodo-derivative was confirmed by the high-field-shifted ^13^C NMR signal of C-5 at 9 ppm. Alternative approaches utilizing displacement of a 5-*O*-substituted triflate, mesylate, or tosylate group were less efficient due to concomitant formation of a cyclic *C*-glycosidic byproduct. Next, the Arbuzov reaction of **18** in triethylphosphite at 150 °C gave *C*-phosphonate **19**. Global deprotection of **19** involved cleavage of the ethyl phosphonate using the McKenna reaction^21^ with trimethylsilyl bromide in pyridine followed by hydrogenation in the presence of Pd-hydroxide. Again, this step also generated byproducts due to the loss of the *N*-hydroxy as well as the formyl group and formation of reductive amination products. Isolation of the target compound **20** had thus to rely on HILIC-separation and careful NMR analysis of separated fractions prior to pooling and lyophilization. Structures of compounds **17** and **20** were eventually fully confirmed by NMR analysis, which revealed ^31^P NMR signals for **17** at 2.97/2.92 and 20.54 ppm for compound **20**, respectively.

**Scheme 3:**
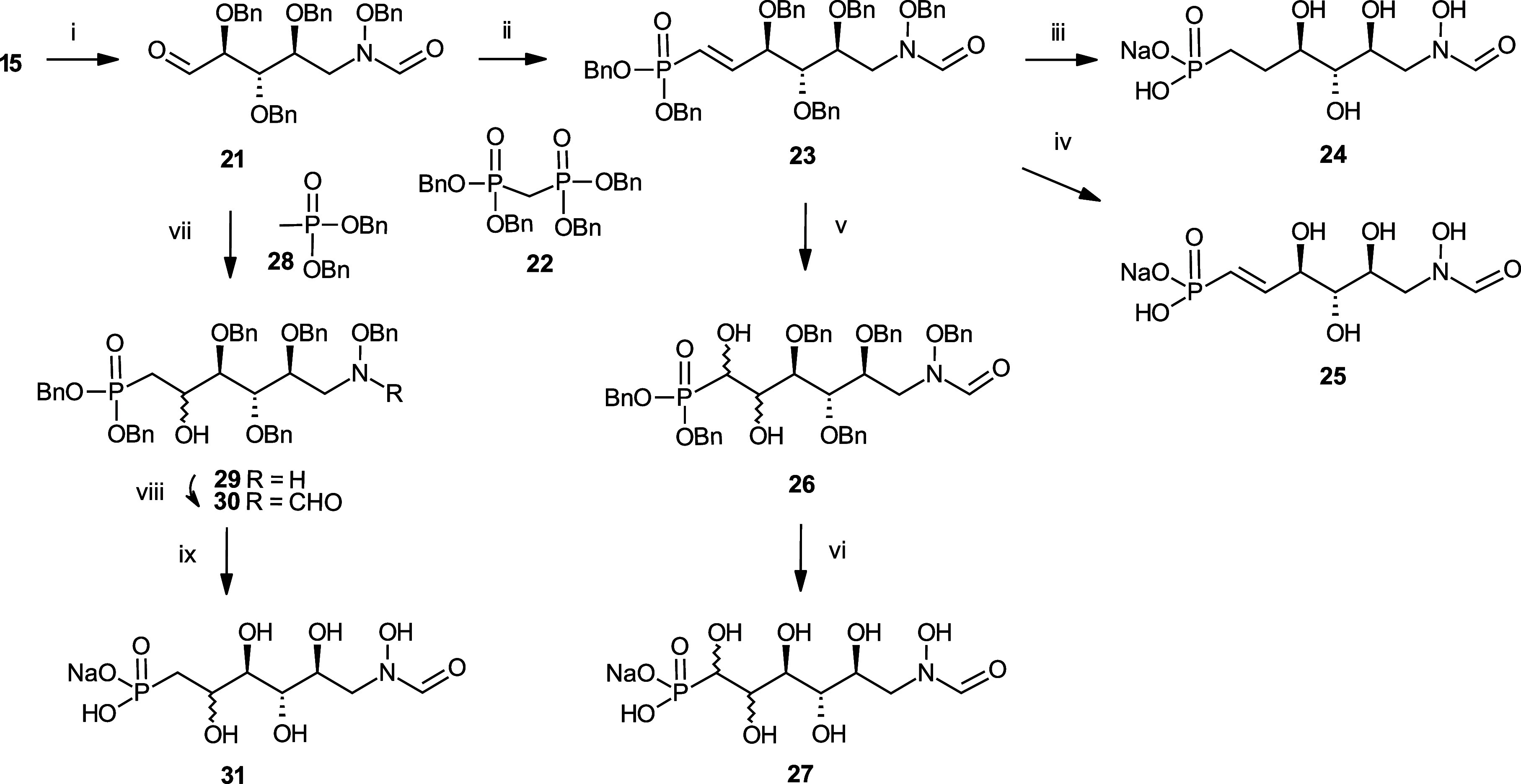
Synthesis of the *N*-formyl-*N*-hydroxy Inhibitors **24**, **25**, **27**, and **31** Reagents and Conditions: (i) Dess-Martin ox, 100%; (ii) **22**, BuLi, THF, 76%; (iii) Pd(OH)_2_, aq. THF, MeOH, 16%; (iv) SnCl_4_, 54%; (v) NMO, K_2_OsO_4_, 79%; (vi) Pd(OH)_2_, aq. THF, 24%; (vii) **28**, BuLi, THF, −78 °C, 22%; (viii) CDI, 73%; (ix) Pd(OH)_2_, aq. THF, 9%.

In order to study the impact of the distal fragment, additional analogues containing a hydrolytically stable *C*-phosphonate group with or without additional hydroxyl groups at the chain extension were synthesized (Scheme 3). Oxidation of the primary alcohol **15** under Dess-Martin conditions proceeded smoothly to give the aldehyde **21** which was directly used for the chain elongation steps. Wittig–Horner reaction of **21** with the lithium salt of tetrabenzylphosphonate **22**(22) at −78 °C gave the *E*-configured alkene **23**. Deprotection of **23** was performed with and without preserving the double bond. Hydrogenation of **23** in the presence of Pd(OH)_2_ again met with difficulties due to significant byproduct formation. Repeated HILIC chromatography enabled the isolation of a small amount of pure *N*-formyl derivative **24**, which was fully characterized by NMR and HRMS analysis. Alternatively, benzyl groups were removed by the action of the Lewis acid SnCl_4_,^23^ followed by extensive HILIC purification of the reaction mixture. The presence of the double bond in **25** was confirmed by the characteristic ^1^H and ^13^C NMR data (see Supporting Information). Introduction of additional hydroxy groups was accomplished by the catalytic *cis*-dihydroxylation of **23** with NMO and potassium osmate in aqueous acetone.

The diastereomeric mixture **26** was not separated and was directly subjected to deprotection as described above to furnish compound **27**. Introduction of a single hydroxy group was carried out by reaction of the aldehyde **21** with the lithium salt of dibenzyl methanephosphonate **28** in THF, which resulted in a low yield of the *N*-formyl product **30**. In addition, the deformylated derivative **29** was isolated in 22% yield and unreacted aldehyde **21** was recovered in 28% yield. In order to generate additional amounts of the formyl derivative **30**, formylation of **29** was performed with formic acid and carbonyldiimidazole that gave **30**. Deprotection of **30** by hydrogenolysis in the presence of Pd(OH)_2_ afforded **31** as a diastereomeric mixture.

**Scheme 4:**
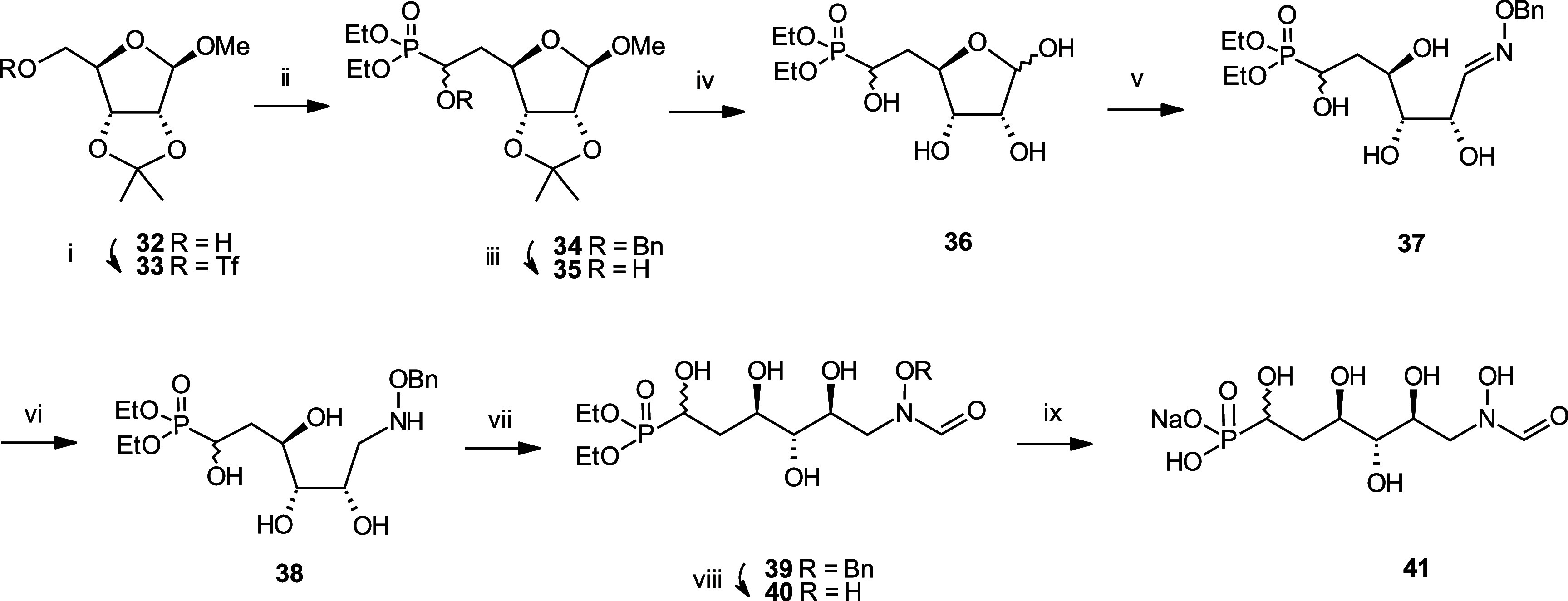
Synthesis of the *N*-formyl-*N*-hydroxy Inhibitor **41** Reagents and Conditions: (i) Tf_2_O, DCM, −10 °C, 86%; (ii) Diethyl [(benzyloxy)methyl]Phosphonate, nBuLi, diisopropylamine, THF, −78 °C, 61%; (iii) H_2_, 10 bar, Pd/C, EtOH, 50 °C, 95%; (iv) Amberlyst 15, water, 60 °C; (v) Benzylhydroxylamine, NaHCO_3_, MeOH, water, 60 °C, 56% over two steps; (vi) 1. BH_3_.THF, THF 0 °C. 2. MeOH, 60 °C, 78%; (vii) Trifluoroethyl formate, THF, 60 °C, 65%; (viii) H_2_, 1 bar, Pd/C, MeOH, rt, 97%; (ix) 1. TMSBr, pyr, DCM, 5 °C. 2. NaHCO_3_, MeOH, water, 45%.

Synthesis of distal hydroxyl analogue **41** started with conversion of **32** to triflate **33** with trifluoromethanesulfonic anhydride (Scheme 4). Nucleophilic substitution of **33** with [diethylphosphono(benzyloxy)methyl]lithium afforded **34**, which was debenzylated under catalytic hydrogenation conditions. **35** was demethylated using Amberlyst 15, and the resulting ribose analogue **36** was converted into **37** using benzylhydroxylamine. Oxime **37** was then reduced using borane in THF, and the corresponding hydroxylamine **38** was carbonylated with trifluoroethyl formate to afford **39**. Debenzylation under catalytic hydrogenation conditions followed by hydrolysis of the diethylphosphonate using TMSBr produced **41**.

**Scheme 5:**
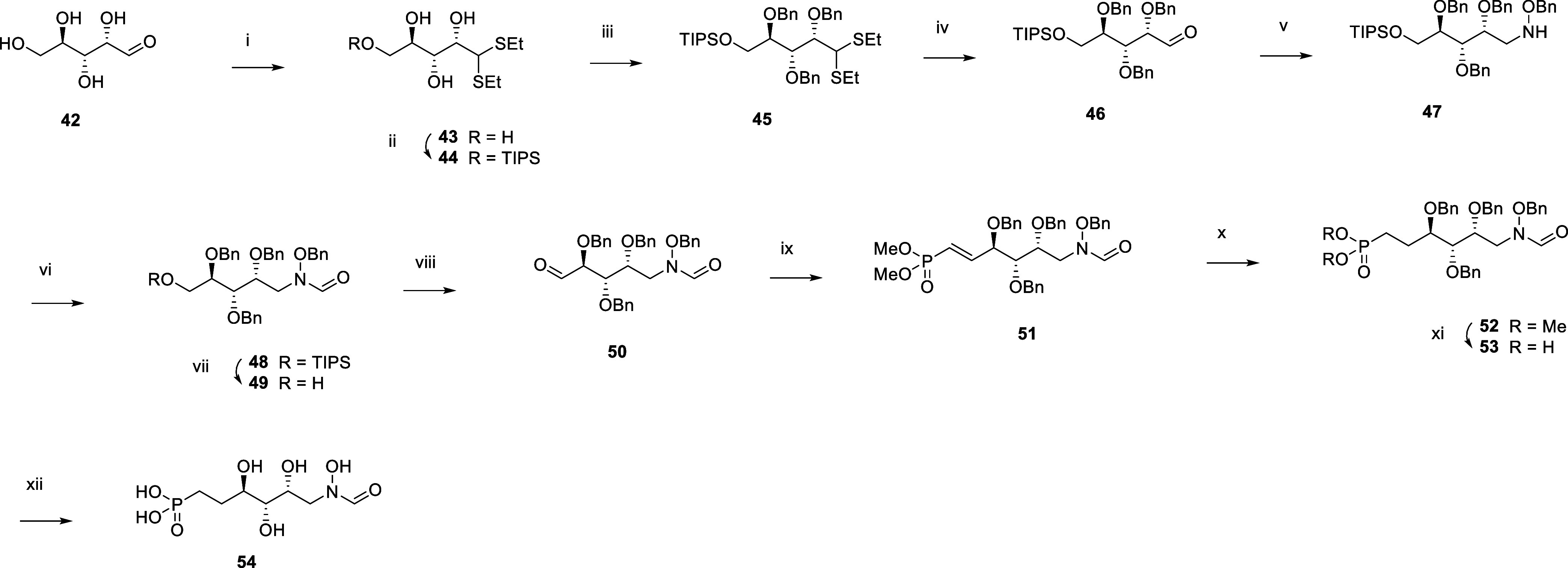
Synthesis of the *N*-formyl-*N*-hydroxy Inhibitor **54** Reagents and Conditions: (i) EtSH, HCl, 0 °C, 83%; (ii) TIPSCl, DMAP, pyr, rt, 87%; (iii) Benzyl bromide, NaH, DMF, 0 °C to rt, 51%; (iv) NBS, acetone, water, 0 °C; (v) 1. H_2_NOBn.HCl, pyr, MeOH, 60 °C. 2. NaCNBH_3,_ AcOH, rt, 60% over two steps; (vi) CDI, HCO_2_H, TEA, THF, 0 °C to rt, 77%; (vii) TBAF, THF, rt, 91%; (viii) DMP, DCM, 0 °C to rt, 48%; (ix) Tetramethyl methylenediphosphonate, NaH, Et_2_O, 0 °C, 55%; (x) H_2_, 1 bar, Pt/C, EtOAc, rt, 85%; (xi) TMSBr, pyr, DCM, 0 °C to rt, 78%; (xii) H_2_, 1 bar, Pd(OH)_2_, THF, AcOH, water, rt, 7%.

To study the impact of the absolute configuration of hydroxyl substituents, **54**, the 2-epimeric form of **17** was made (Scheme 5). Synthesis started with the conversion of tetrahydroxypentanal **42** into dithiane **43** using ethanethiol. After protection of the primary alcohol using TIPSCl and benzylation of the remaining hydroxyl groups with benzyl bromide, **45** was converted into aldehyde **46** using *N*-bromosuccinimide. Reducing the level of amination with benzylhydroxylamine and sodium cyanoborohydride afforded **47**. Carbonylation with carbonyldiimidazole followed by TBAF mediated silyl deprotection produced **49**, which was oxidized into aldehyde **50** using Dess-Martin periodinane. Wittig reaction with tetramethyl methylenediphosphonate, phosphonic ester deprotection using TMSBr, and final debenzylation under catalytic hydrogenation conditions afforded **54**.

**Scheme 6:**
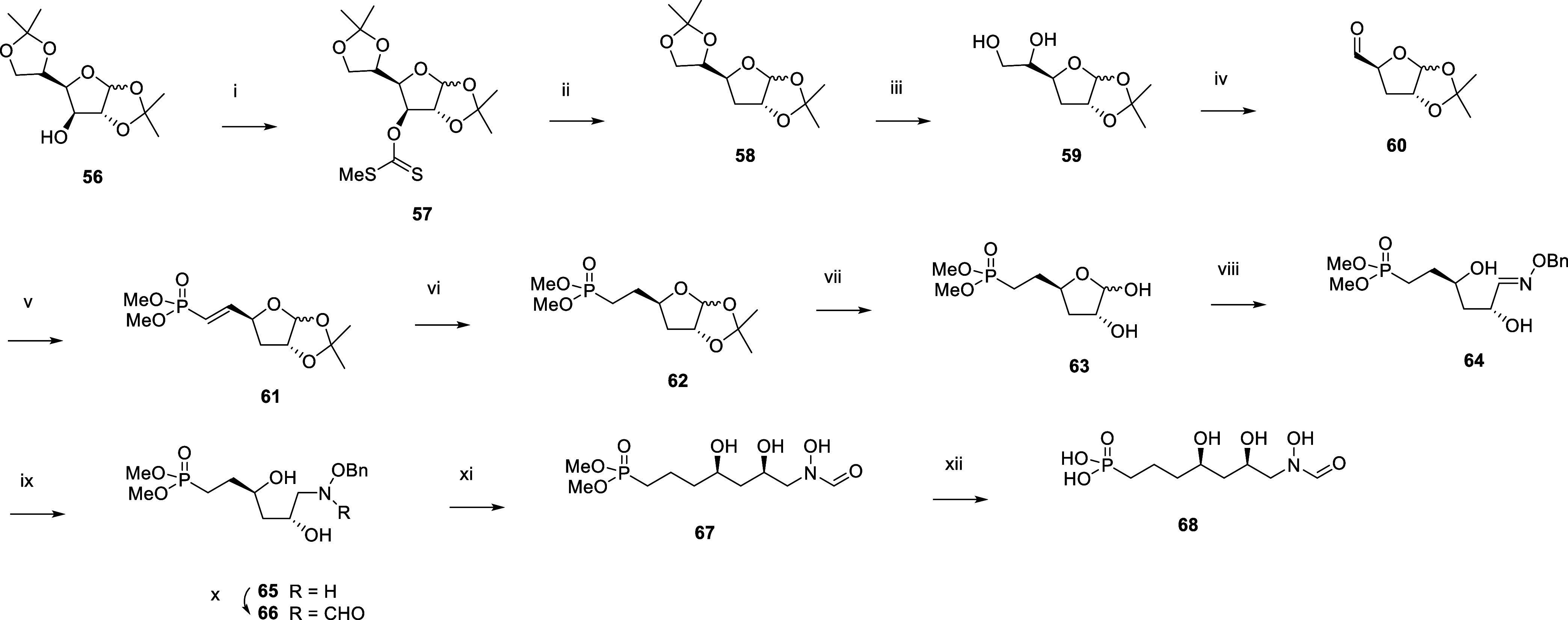
Synthesis of the *N*-formyl-*N*-hydroxy Inhibitor **68** Reagents and Conditions: (i) NaH, CS_2_, MeI, imidazole, THF, 0 °C to rt; (ii) Bu_3_SnH, AIBN, toluene, reflux, 76% over two steps; (iii) aq. H_2_SO_4_, MeOH, 0 °C, 60%; (iv) NaIO_4_, MeOH, rt; (v) Tetramethyl methylenediphosphonate, NaH, Et_2_O, 0 °C to rt, 60% over two steps; (vi) H_2_, 1 bar, Pd/C, MeOH, rt; (vii) aq. AcOH, 80 °C, 40% over two steps; (viii) H_2_NOBn.HCl, pyr, MeOH, 60 °C, 67%; (ix) NaCNBH_3,_ AcOH, 0 °C to rt, 88%; (x) Trifluoroethyl formate, THF, 65 °C, 51%; (xi) H_2_, 1 bar, Pd(OH)_2_, THF, AcOH, water, rt; (xii) TMSBr, pyr, DCM, 0 °C, 12% over two steps.

To study the impact of removing a hydroxyl group on the inhibitory activity, analogue **68** was made (Scheme 6). Deoxygenation of glucofuranose **56** was achieved with carbon disulfide, sodium hydride, and methyl iodide to afford dithiocarbonate **57**, which was submitted to radical reduction with tributyltin hydride and AIBN. Selective acetonide deprotection using aqueous sulfuric acid in methanol was followed by oxidative cleavage with sodium periodate to afford **60**. Wittig reaction with tetramethyl methylenediphosphonate, subsequent catalytic hydrogenation to reduce the carbon–carbon double bond, and acetonide deprotection using acetic acid yielded **63**, which was further converted into **68** using the reaction suite analogous to the one described in Scheme 4.

**Scheme 7:**
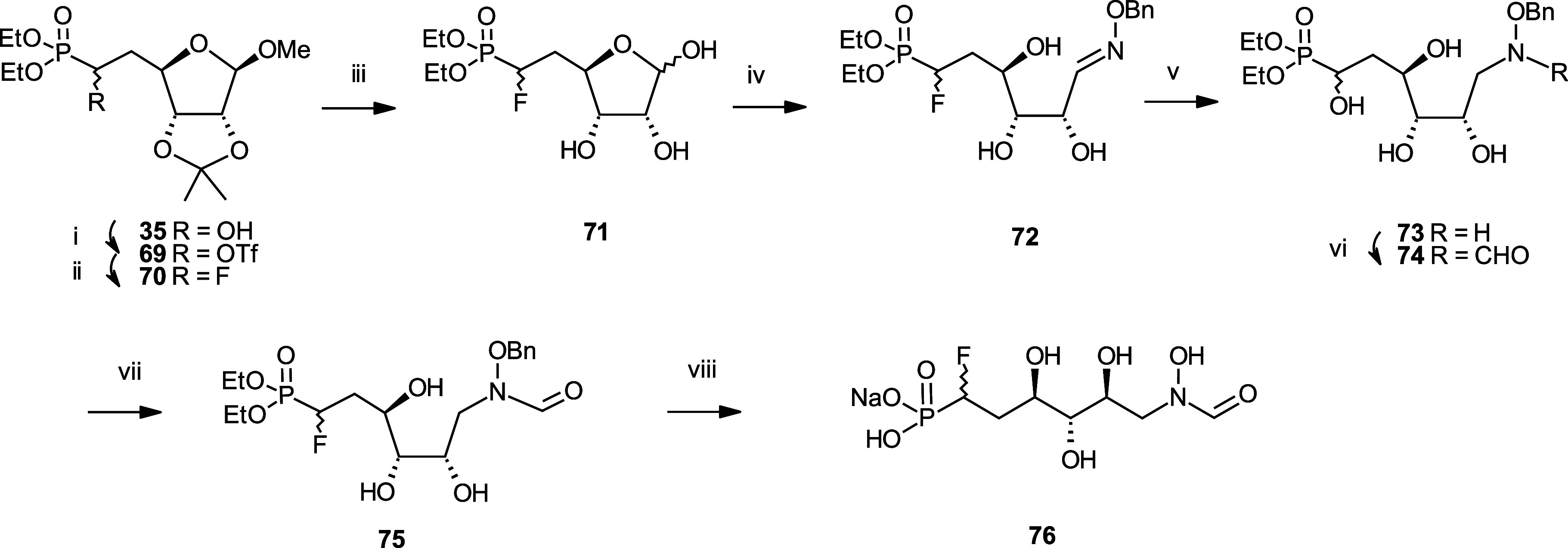
Synthesis of the *N*-formyl-*N*-hydroxy Inhibitor **76** Reagents and Conditions: (i) Triflic anhydride, pyr, DCM, −10 °C; (ii) CsF, tBuOH, 40 °C, 58% over two steps; (iii) Amberlyst 15, water, 60 °C; (iv) H_2_NOBn.HCl, NaHCO_3_, EtOH, water, 60 °C, 81% over two steps; (v) 1. BH_3_.THF, THF, 0 °C. 2. MeOH, 60 °C, 71%; (vi) Trifluoroethyl formate, THF, 60 °C, 63%; (vii) H_2_, 1 bar, Pd/C, MeOH, rt, 90%; (viii) TMSBr, pyr, DCM, 0 °C, 40%.

**Scheme 8:**
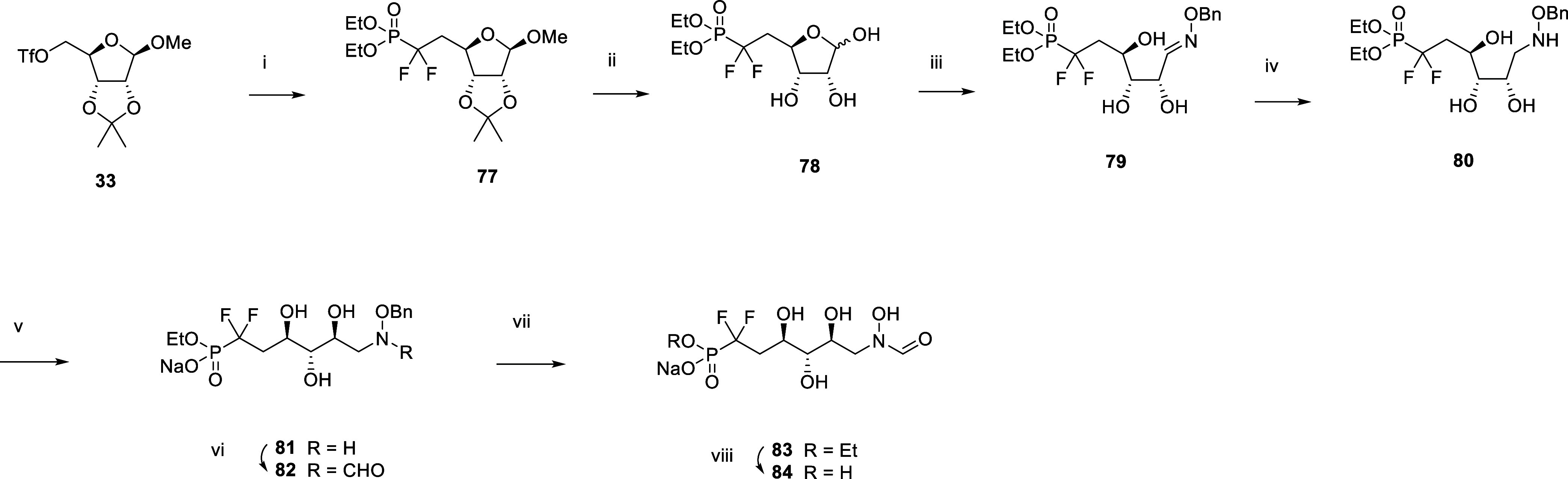
Synthesis of the *N*-formyl-*N*-hydroxy Inhibitor **84** Reagents and conditions: (i) nBuLi, diisopropylamine, tris(*N,N*-tetramethylene)phosphoric acid triamine, diethyl(difluoromethyl)phosphonate, THF −78 °C, 18%; (ii) Amberlyst 15, water, 60 °C; (iii) H_2_NOBn.HCl, NaHCO_3_, water, EtOH, 60 °C, 73% over two steps; (iv) 1. BH_3_.THF, THF, 0 °C. 2. MeOH, 60 °C, 71%; (v) aq. NaOH, water, rt, 84%; (vi) Trifluoroethyl formate, THF, rt, 29%; (vii) H_2_, 1 bar, Pd/C, MeOH, rt; (viii) TMSBr, pyr, DCM, 0 °C, 66% over two steps.

Then, we focused our efforts on the synthesis of distal fluorinated analogues **76** and **84** (Schemes 7 and 8). The synthesis of monofluorinated **76** started with the conversion of intermediate **35** into **71** via its triflation with trifluoromethanesulfonic anhydride, followed by nucleophilic substitution with cesium fluoride. The reaction suite analogous to the one already described in Scheme 4 involving oxime formation, reduction, carbonylation, and phosphonate ester hydrolysis was used to produce **76**. Difluorinated analogue **84** was made similarly from intermediate **77**, which was obtained via nucleophilic substitution of triflate **33** with diethyl(difluoromethyl)phosphonate.

**Scheme 9:**
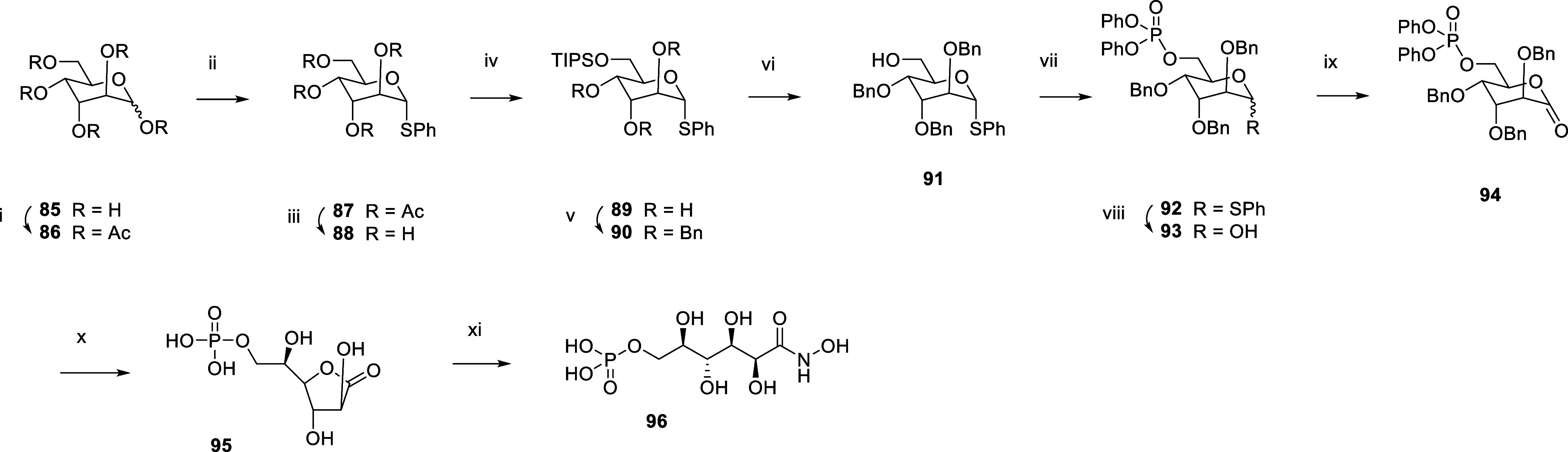
Synthesis of the Hydroxamic Acid Inhibitor **96** Reagents and Conditions: (i) Ac_2_O, pyr, DMAP, rt; (ii) PhSH, BF_3_. Et_2_O, DCM, 0 °C to rt, 82% over two steps; (iii) TEA, water, MeOH, rt, 91%; (iv) TIPSCl, DMAP, rt, 89%; (v) BnBr, NaH, DMF, rt, 96%; (vi) TBAF, THF, rt, 64%; (vii) Diphenyl phosphochloridate, TEA, DMAP, DCM, rt, 97%; (viii) NBS, acetone, water, −11 to 0 °C, 98%; (ix) PCC, DCM, rt, 65%; (x) 1. H_2_, 1 bar, Pd/C, THF, rt. 2. H_2_, 1 bar, PtO_2_, THF, rt, 90%; (xi) aq. NH_2_OH, rt, 88%.

The reverse form of hydroxamic acid **17** was also produced (analogue **96**, Scheme 9). Synthesis started with the peracetylation of d-altrose **85** using acetic anhydride. Thioether protection of the anomeric position using thiophenol and boron trifluoride, followed by acetyl deprotection with triethylamine in water and methanol, afforded **88**. The primary alcohol was silylated with TIPS chloride, and the remaining hydroxyl groups were benzylated with benzyl bromide. Deprotection of the primary alcohol with TBAF followed by phosphorylation with diphenyl phosphochloridate afforded **92**. Conversion of thioether into hemiacetal **93** was achieved with *N*-bromosuccinimide. Oxidation with pyridinium chlorochromate afforded ester **94**, which was debenzylated and dephenylated to **95** by catalytic hydrogenation. Final lactone transamidification with aqueous hydroxylamine afforded hydroxamate **96**.

## Results And Discussion

**Scheme 10:**
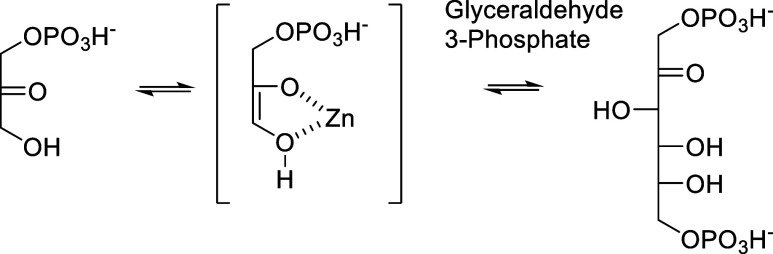
Mechanism of Class II Fbas

On the basis of the critical presence of zinc in the catalytic site of GmhA^13^ and the hypothesis that this metal stabilizes the high energy enediolate intermediate as this is the case for other bacterial enzymes such as class II fructose bis-phosphate aldolases (Fbas)^24^ (Scheme 10), we have set out to exploit the strong zinc chelating ability of hydroxamic acids^16^ to design our inhibitors. Precedents such as phosphoglycolohydroxamic acid^25^ have been reported for the inhibition of Fbas, and *N*-formyl hydroxamate derivatives^26^ for the inhibition of peptide deformylase (PDF). Studies based on crystal structures, ab initio calculations, and IR-measurements of formylhydroxamic acid in gas phase indicated that the Z1-tautomeric form is the predominant one, thus being well suited for interaction with the catalytic Zn^2+^ ion.^27^

**Figure 1:**
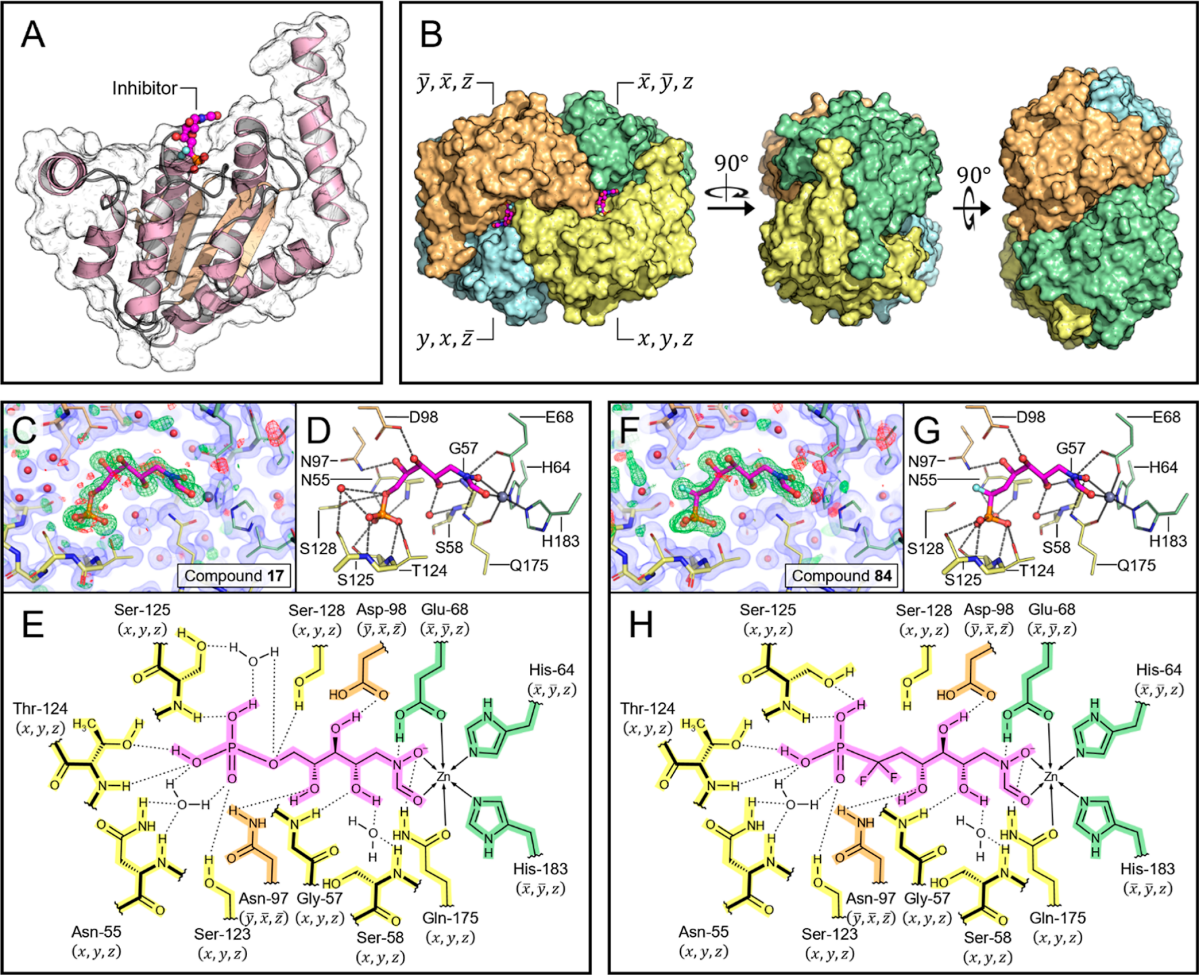
Crystal structures of GmhA in complex with different orthosteric inhibitors. (A) ASU of a GmhA-inhibitor cocrystal structure. The ASU contains one copy of GmhA and one copy of inhibitor. (B) GmhA tetramer assembled from 4 symmetry related ASUs. Each of the 4 active sites contains residues from 3 separate chains of GmhA. (C–E) Co-crystal structure of GmhA with compound **17** deposited under PDB accession code 8V4J. (F–G) Co-crystal structure of GmhA with compound **84** deposited under PDB accession code 8V2T. (C,F) OMIT map verifying the presence of inhibitor in the crystal structure. Observed electron density is represented by a blue volume, while positive and negative features of the OMIT map are shown on green and red meshes, respectively. (D,G) Hydrogen-bonding and zinc-chelating interactions that stabilize the inhibitors in the GmhA active site. (E,H) 2D representation of the same interaction network. GmhA residues are colored according to the ASU, while the inhibitor molecule is labeled in magenta.

Evidence for the primary role of Zn^2+^ ion chelation in the active site by the *N*-formyl hydroxamate moiety was then obtained by crystallizing GmhA followed by soaking with inhibitors **17** and **84**, respectively. The crystal structures of GmhA with compound **17** and compound **84** were both solved in the *P*4_2_2_1_2 space group (Table S1, Supporting Information), revealing a single copy of GmhA, inhibitor, and zinc ion in the asymmetric unit (ASU) (Figure 1A,E,H). The unit cell contains 8 symmetry-related copies of the ASU, forming two identical GmhA tetramers. The GmhA tetramer contains 4 active sites that are each occupied by a zinc ion and an inhibitor (Figure 1B). Both inhibitors chelate the Zn^2+^ ion at two coordination sites via the hydroxamic acid moiety that is common to both molecules (Figure 1D,G). The Zn^2+^ metal center is further coordinated by other donor atoms from residues spanning two chains of GmhA, yielding a Zn complex with an octahedral geometry. An extensive hydrogen-bonding network involving GmhA residues and structured solvent molecules further stabilizes each inhibitor in the active site (Figure 1E,H). Compound **84** appears to have greater overall shape complementarity with the GmhA active site due to the additional “bulkiness” of the difluorophosphonate group compared to that of the phosphate group in **17**. However, the phosphate group in **17** appears to accommodate additional H-bonds that are not possible with **84** (Figure 1C,E,F,H).

**Figure 2:**
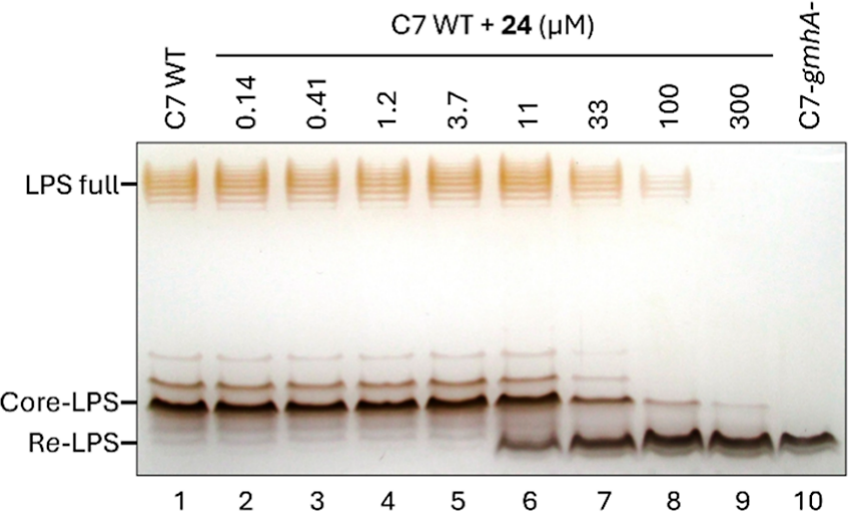
Silver-stained SDS-PAGE of the LPS of *E. coli* C7 grown with 0 to 300 μM compound **24** (columns 1 to 9) and control heptose-deficient R*e*-LPS of *gmhA*-deleted strain (column 10).

The GmhA inhibitory activity of our sedoheptulose-7-phosphate hydroxamic acid analogues was tested by using a luminescent assay (see the experimental section), which allowed the determination of IC_50_ values (Table 1). The ability of these analogues to inhibit LPS biosynthesis in wild-type *E. coli* was also visualized on silver-stained LPS SDS-PAGE electrophoretic images (Figure 2) using strain C7 (018:K1:H7), which is a newborn meningitis *E. coli* strain containing inner- and outer-core oligosaccharides as well as the O-antigen. Inhibition was monitored by formation of the heptose-deficient R*e* LPS, which only comprises lipid A and Kdo, and the EC_50_ data are reported in Table 1.

**Table 1:**
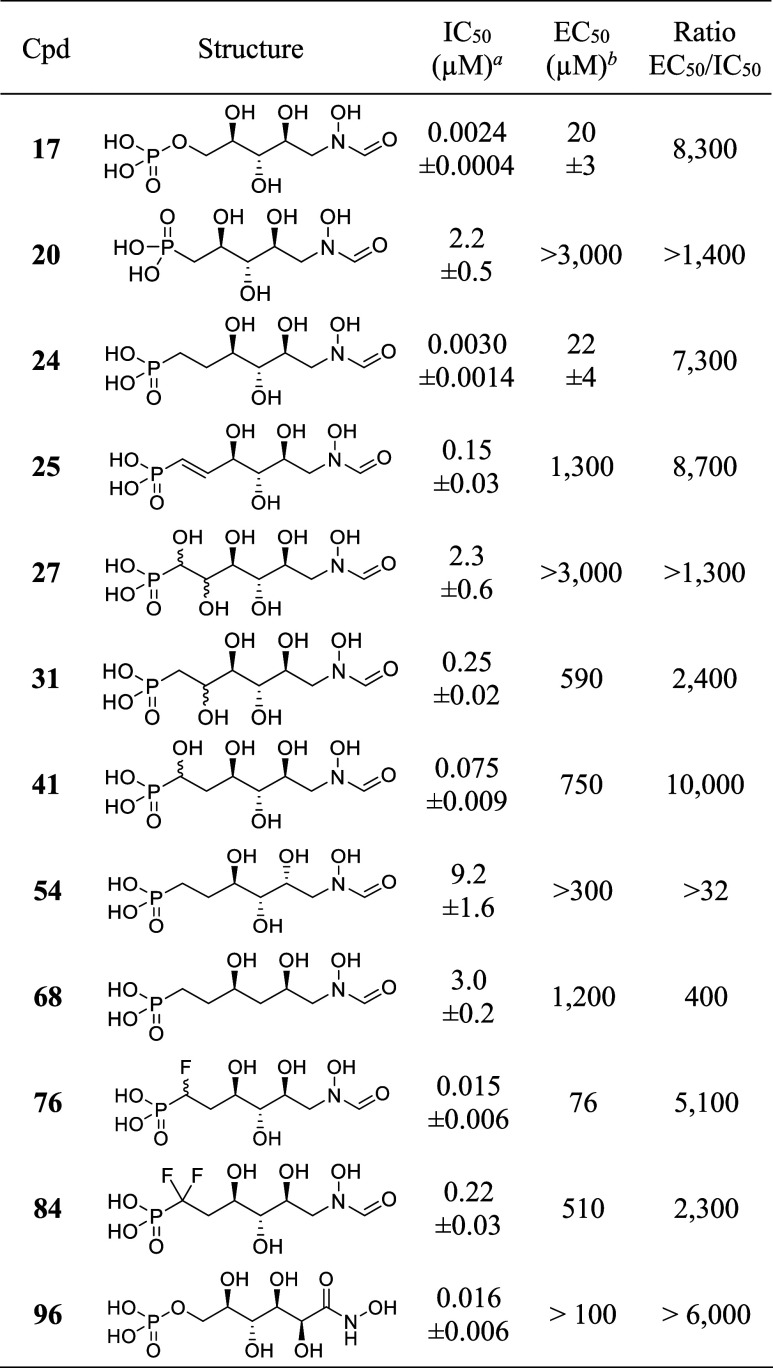
*E. coli* GmhA and LPS Biosynthesis Inhibitory Activity of Sedoheptulose-7-Phosphate Hydroxamic Acid Analogues

a Inhibition of the enzymatic activity of *E. coli* GmhA by luminescence assay. Mean ± SD of at least 3 independent experiments.

b Inhibition of *E. coli* LPS biosynthesis by SDS-PAGE electrophoresis. Mean ± SD of 3 independent experiments for **17** and **24**, *n* = 1 for other compounds.

The hydroxamic acid analogue **96** of sedoheptulose-7-phosphate **1** proved to be a potent GmhA inhibitor with an IC_50_ in the low nanomolar range. This compound did not inhibit *E. coli* LPS biosynthesis up to 100 μM but started to affect bacterial growth above this concentration. The reverse *N*-formyl hydroxamate analogue **17** displayed an even better GmhA inhibitory activity at 2.4 ± 0.4 nM, with this time an observable effect on LPS biosynthesis at an EC_50_ of 20 ± 3 μM. This encouraging result prompted us to investigate its phosphonate analogue **24**, which should be more stable to in vivo phosphatases than its phosphate counterpart. Gratifyingly, compound **24** also displayed low nanomolar GmhA inhibition at 3.0 ± 0.2 nM and LPS biosynthesis inhibition at 22 ± 4 μM. To further explore the structure–activity relationship around phosphonate analogue **24**, we prepared and screened several closely related derivatives: the shorter analogue **20**, the des-hydroxyl derivative **68**, the diastereoisomer **54**, the ene-analogue **25**, and the hydroxylated derivatives **41**, **31,** and **27**. Although inhibiting GmhA to various degrees, none of these analogues proved as potent as phosphonate **24**. Of note, the des-hydroxyl derivative **68** displays a lower EC_50_/IC_50_ ratio than the other heptose analogues, which may reflect a better UhpT transport to the cytosol.

**Figure 3:**
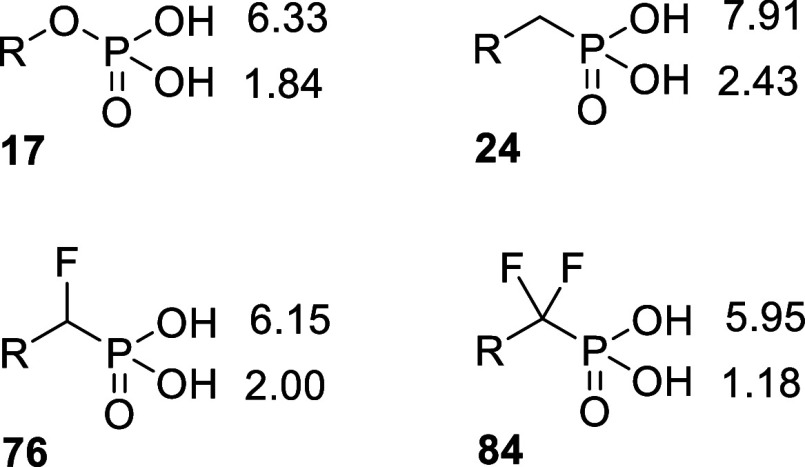
Predicted p*K*_*a*_ of phosphate **17**, phosphonate **24**, monofluorophosphonate **76**, and difluorophosphonate **84** according to ACD Laboratories V12.5.

Since α-halogenated phosphonates are known to be potentially better bioisosteres of the phosphates than the nonhalogenated phosphonates,^28^ we turned our attention to the α-fluorinated analogues of phosphonate **24** in the hope of improving enzymatic inhibition and active transport. The parameters that may favor α-fluorinated phosphonates over their nonfluorinated congeners are a reduced p*K*_a_, an increased C–CHF–P or C–CF_2_–P dihedral angle, and the possibility of forming fluorine-hydrogen bonding. A p*K*_*a*_ prediction analysis of phosphate **17** vs phosphonate **24** and its fluorinated analogues **76** and **84** indeed indicates that the monofluorinated phosphonate has closer p*K*_a_ values to the phosphate than the nonhalogenated phosphonate (Figure 3). Although still displaying GmhA inhibition in the low nanomolar range, the monofluorinated diastereomeric **76** did not demonstrate any improvement over phosphonate **24**, possibly due to stereoelectronic factors disfavoring fluorine vs hydrogen in this enzyme location, a trend confirmed by the even lower activity of its difluorinated congener **84**.

**Figure 4:**
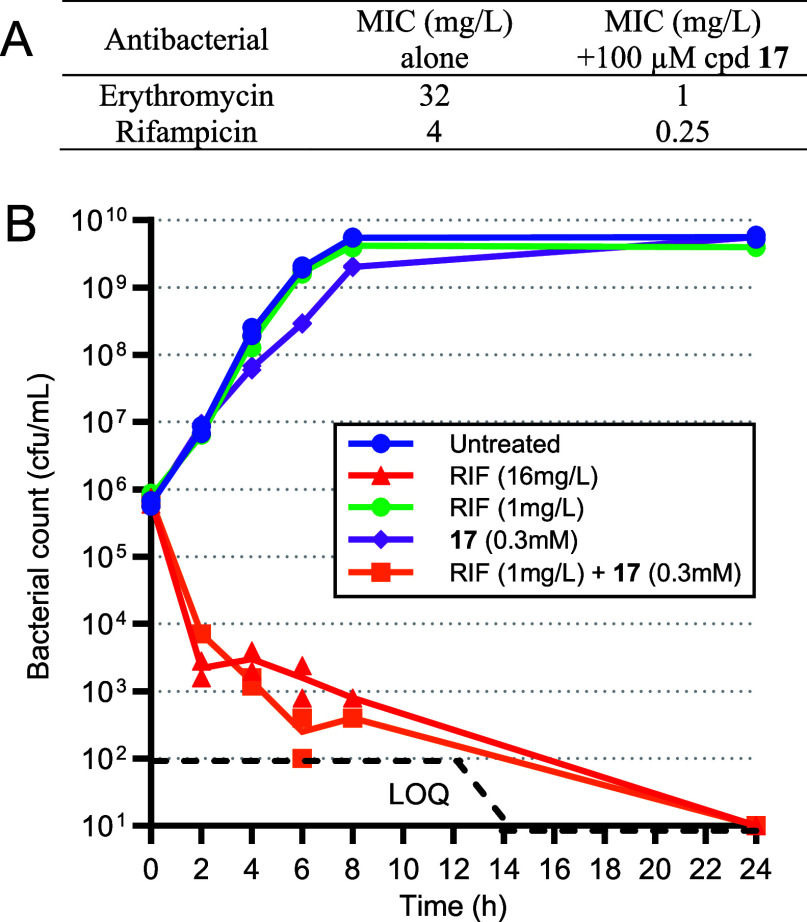
Combination of compound **17** with antibacterials. (A): *E. coli* C7 MIC of Erythromycin and Rifampicin alone or combined with 100 μM **17** (*n* = 1). (B): Time-kill kinetics of *E. coli* C7 with Rifampicin (RIF) alone or combined with compound **17**. Bacterial enumeration replicates are represented in the plot. LOQ: limit of quantification.

Then, we investigated the cytotoxicity and the ability of such inhibitors to potentiate the antimicrobial activity of macrolide and rifamycin antibiotics. Phosphate **17** and phosphonate **24** were devoid of HepG2 cytotoxicity (CC_50_ > 1000 μM, Table 2), and, as expected by their mode of action, did not display any antibacterial activity (*E. coli* C7MIC > 1000 μM, Table 2). Yet at a concentration of 27 mg/L (100 μM), **17** displayed in the presence of the UhpT inducer glucose-6-phosphate (100 μM) a 32-fold potentiation of erythromycin (MIC from 32 to 1 mg/L) and a 16-fold potentiation of rifampicin (MIC from 4 to 0.25 mg/L) against *E. coli* C7. Against the same strain, the combination of **17** (300 μM) with rifampicin (1 mg/L) was rapidly bactericidal with more than 3 log reduction of the bacterial inoculum in less than 6 h and no detectable counts at 24 h, whereas the same compounds alone did not alter at all the bacterial growth (Figure 4). By inhibiting the first committed step in bacterial heptose biosynthesis in wild-type *E. coli*, this derivative is thus able to remove the hydrophilic outer core of Gram-negative bacteria and sensitize it back to a lipophilic macrolide and a rifamycin antibiotic.

**Table 2:** Minimum Inhibitory Concentration (MIC) on *E. coli* C7 and Cytotoxic Concentration CC_50_ on HepG2 Cells of Compounds **17** and **24** (*n* = 2)

compound	MIC *E. coli* C7 (μM)	CC_50_ HepG2 (μM)
**17**	>1000	>1000
**24**	>1000	>1000

Compound **17** was also tested against clinical isolates of *E. coli,Escherichia cloacae*, and *K. pneumoniae* for its ability to block LPS heptosylation (EC_50_). As detailed in Table 3, **17** in the presence of the UhpT inducer glucose-6-phosphate (100 μM) is a specific inhibitor of LPS heptosylation against various strains, including multiresistant clinical isolates expressing ESBL, AmpC, or NDM-1.

**Table 3:** EC_50_ of LPS Biosynthesis Inhibition of Clinical Isolates by Compound **17** in the Presence of 100 μM Glucose-6-Phosphate (*n* = 1)

strain	resistance mechanism	EC_50_ of LPS heptosylation by **17** (μM)
*E. coli* S1	ESBL	21
*E. cloacae* S2	AmpC	18
*K. pneumoniae* S3	NDM-1	21

**Figure 5:**
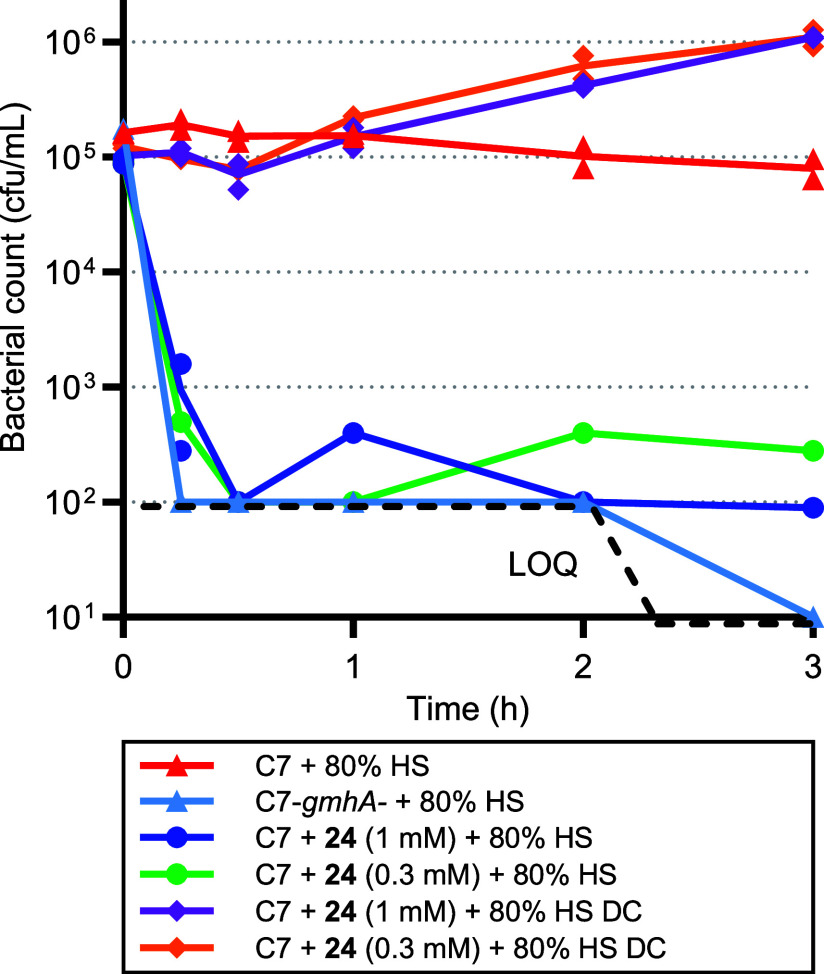
Time-kill kinetics of *E. coli* C7 wild type or *gmhA*-deleted strain in the presence of 80% human serum untreated or heated (DC). Bacteria are pregrown in LB broth for 5 h in absence or presence of compound **24** at 0.3 and 1 mM. Bacterial enumeration replicates are represented on the plot. LOQ: limit of quantification.

Finally, *E. coli* C7 bacteria grown for 5 h in the presence of 0.3 and 1 mM compound **24** were as susceptible as the *gmhA*-deleted strain to 80% human serum, with a 3 log reduction of the bacterial load in 30 min (Figure 5). The treatment of *E. coli* with a GmhA inhibitor was therefore able to sensitize the strain to the antibacterial innate components present in the serum, such as the complement, thus validating the concept of bacterial antivirulence together with the permeabilizing effect to other antibacterial agents.

## Conclusions

We report here the synthesis and structure–activity relationship of various sedoheptulose 7-phosphate analogues containing a hydroxamic acid moiety designed to target the zinc-bearing catalytic site of GmhA as confirmed by two liganded high-resolution diffraction data. Some of these phosphoryl and phosphonyl derivatives demonstrate low nanomolar GmhA inhibition. They are also able to reach their cytosolic target and inhibit LPS biosynthesis in a wild-type *E. coli* strain at low micromolar concentrations. The most active hydroxamates are phosphates **17** and **96**, phosphonate **24**, and monofluorophosphonate **76**, while distal hydroxylation or epimerization or chain shortening proved detrimental to inhibitory activity. In agreement with its mode of action, phosphoryl compound **17** does not display any antibacterial activity of its own but proves its ability to potentiate by 16 to 32-fold the activity of erythromycin and rifampicin on wild-type *E. coli*. These results pave the way toward novel possibilities of treating or preventing bloodstream infections caused by pathogenic Gram-negative bacteria without affecting their commensal flora, thus exerting potentially less selective pressure for antibiotic resistance than conventional antibacterial chemotherapy.

## Experimental Section

### Chemical Syntheses

The synthetic procedures for the preparation of compounds **41**, **54**, **68**, **76,** and **84** are fully described in the patent WO 2014/067904 A1.^34^ Experimental procedures and NMR spectra of compounds **10**–**27** and **85**–**96** have been compiled in the Supporting Information. If necessary, purity of compounds was checked by HPLC. All compounds are >95% purity.

### Bacterial Strains Used in This Study

*E. coli* C7 (R. Debré hospital, Paris), *E. coli* S1, *E. cloacae* S2, and *K. pneumoniae* S3 (Necker hospital, Paris).

### Purification of GmhA

*E. coli* BL21(DE3) (Novagen) cells were transformed with an expression plasmid, described in ref (13), containing full length GmhA from *B. pseudomallei* (strain K96423) with a TEV-cleaved, *N*-terminal hexa-histidine affinity tag. Cells were grown in Luria–Bertani (LB) broth supplemented with kanamycin (20 mg/L) to an optical density (OD_600_) of 0.5 prior to induction by the addition of 1 mM isopropyl β-d-1-thiogalactopyranoside (IPTG). Following a 3 h induction at 37 °C, cells were harvested and resuspended in buffer (Buffer A) containing 20 mM Tris–HCl, pH 8.0, 750 mM KCl, 1 mM β-mercaptoethanol (BME), and 3 mM imidazole. Cells were lysed by three passes through a French pressure cell at 20,000 psi. Clarified lysate was loaded onto a HisTrap-HP nickel affinity column (GE healthcare, United States of America) equilibrated with Buffer A. The protein was eluted after step washes of Buffer A supplemented with 15, 30, and 60 mM imidazole. GmhA was eluted at a final concentration of 210 mM imidazole. Protein was then further purified by anion exchange chromatography. GmhA containing fractions were buffer exchanged into a buffer (Buffer B) containing 20 mM Tris–HCl, pH 8.0, 1 mM EDTA, 10 mM DTT, 100 mM KCl, and 10% v/v glycerol. This sample was then applied to a 10/100 Mono-Q hp column (GE healthcare, United States of America) and eluted with Buffer B via a linear salt gradient of 100–500 mM KCl. Fractions containing pure GmhA were pooled and buffer exchanged into a crystallization buffer containing 10 mM HEPES, pH 7.0, 500 mM KCl, and 10% w/v PEG 4000 for cryoprotection. Purified GmhA was then concentrated via centrifugation (Vivaspin) to a final concentration of 10 mg/mL and stored at −80 °C.

### Crystallization of GmhA

A solution of purified GmhA [10 mg/mL GmhA, 500 mM KCl, 10% (w/v) PEG4000, 10 mM HEPES, pH 7.0] was mixed in a 1:1 ratio with a crystallization solution (100 mM sodium acetate, pH 4.6) and sealed over a reservoir containing 800 μL of 1.5 M (NH_4_)_2_SO_4_. Crystals formed within 48 to 72 h of equilibration at 4 °C. Drops containing crystals were then soaked with 0.2 μL of inhibitor solution (50 mM **17** or **84**, 50 mM HEPES, pH 7.5) for 90 min. Following soaking, crystals were harvested, flash frozen, and stored in Liquid N_2_ until data collection.

### X-ray Diffraction Data Collection

Frozen crystals were mounted in a 100 K nitrogen cryostream at beamlines X-25 and X-29 of the National Synchrotron Light source at Brookhaven National Laboratories. X-ray diffraction data were collected over a total of 180° of rotation about the omega axis. Crystals containing **17** were diffracted at a wavelength of 1.075 Å at beamline X-29 with a frame width of 0.3°, while **84** was diffracted at 1.100 Å at beamline X-25 with a width of 0.5°. X-ray diffraction data were deposited to the Zenodo data repository.^29,30^

### Structure Determination and Refinement

X-ray diffraction data from liganded **17** and **84** crystals were integrated and scaled using autoPROC (Global Phasing Ltd.) to a resolution limit of 1.31 and 1.40 Å, respectively.^31^ Phenix Phaser^32^ was used to calculate an initial electron density map by molecular replacement (MR) of an existing GmhA crystal structure (PDB: 2XBL).^13^ The structures of both liganded **17** and **84** crystals were solved in the *P*4_2_2_1_2 space group, revealing a single copy of GmhA, inhibitor, and zinc ion in the ASU. The unit cell contained 8 symmetry-related copies of the ASU, forming two identical GmhA tetramers. The atomic coordinates of GmhA and inhibitor were then built and refined against the electron density using Phenix AutoBuild and Phenix Refine.^32^ The refined coordinates of GmhA in complex with **17** and **84** were deposited to the PDB under accession codes 8V4J and 8V2T, respectively.^29,30^ Intermolecular interactions between GmhA, inhibitor, and Zn were analyzed using the protein–ligand interaction profiler^33^ and visualized in PyMOL.

### Inhibition of the Enzymatic Activity of GmhA (Luminescent Assay)

The assay buffer “AB” contained 50 mM HEPES, pH 7.5, 1 mM MnCl_2_, 25 mM KCl, 0.012% Triton-X100, 1 mM dithiothreitol (DTT), and 0.1 μM myelin basic protein (MBP, Sigma, ref M1891). The following components were added in a white polystyrene Costar plate up to a final volume of 30 μL: 10 μL of inhibitor dissolved in DMSO/water 50/50 and 20 μL of GmhA of *E. coli* in AB. After 30 min of preincubation at room temperature, 30 μL of the substrate mixture in AB were added in each well to give a final volume of 60 μL. This reaction mixture was then composed of 2 nM GmhA, 3 μM sedoheptulose-7-phosphate (Sigma), 3 μM ATP (Sigma), and 50 nM HldE of *E. coli* in assay buffer. After 30 min of incubation at room temperature, 100 μL of the revelation mix were added to a final volume of 160 μL, including the following constituents at the respective final concentrations: 10,000 light units/mL luciferase (Sigma), 20 μM D-luciferin (Sigma), and 100 μM *N*-acetylcysteamine (Aldrich). Luminescence intensity was immediately measured on a Luminoskan instrument (Thermofisher) and converted into inhibition percentages. For IC_50_ determinations, the inhibitor was tested at 6 to 10 different concentrations, and the related inhibitions were fitted using XLFit 5 (IDBS) to a classical Langmuir equilibrium model with Hill slope, according to the equation: %inhibition = 100 c^*n*^/(c^*n*^ + IC_50_^*n*^), where c is the compound concentration and n is the Hill coefficient.

### Inhibition of Bacterial LPS Biosynthesis: Bacterial Culture

The compounds to be tested were prepared in deionized water/DMSO (50/50) solutions and added (25 μL) in sterile culture microtubes. The test bacterial isolate was isolated on tryptic soy agar (TSA) overnight. Isolated colonies were cultured in 10 mL of LB medium at 37 °C up to an OD of typically 0.15. These exponentially growing bacteria were finally diluted to 5 × 10^5^ cfu/mL and added in each well (225 μL) for incubation with the compounds at 37 °C for approximately 5 h, up to an OD of ≈0.2–0.4. Some test compounds, e.g., phospho-sugars are actively transported into the bacterial cytosol via the phospho-sugar transporter UhpT (Figure S3). 2.5 μL portion of a 10 mM water solution of glucose-6-phosphate (G6P, 100 μM final concentration, from Sigma) was added in the culture tube in order to induce UhpT expression. LPS extraction: Bacterial cultures were normalized via OD determination, pelleted, and washed with 1 mL of phosphate-buffered-saline (PBS). The pellets were then denatured for 10 min at 95–100 °C in 50 μL of 0.2% sodium dodecyl sulfate (SDS), β-mercaptoethanol 1%, glycerol 36%, Tris 30 mM, pH 7.4, and bromophenol blue 0.001%. Samples were cooled down to room temperature, supplemented with 1.5 μL of proteinase K at 20 mg/mL, incubated for 1 h at 55 °C, and centrifuged for 30 min at 13,000 rpm at 25 °C. The resulting supernatant, containing LPS, was finally analyzed by SDS-PAGE electrophoresis. LPS SDS-PAGE electrophoresis: Polyacrylamide gels (16%/4% acrylamide for separation and concentration, respectively) were prepared, loaded with 8 μL of LPS extracts, and migrated. Silver staining: Gels were incubated overnight in 5% acetic acid/40% ethanol/deionized water, treated by 1% periodic acid/5% acetic acid for 15 min, washed 4 times for 10 min in deionized water, and finally incubated for 18 min in the dark in a silver nitrate solution composed of 56 mL of 0.1 M NaOH (56 mL), 33% aq ammonia (4 mL), 5% AgNO_3_ (45 mL), and deionized water (195 mL). Gels were then washed extensively in deionized water for 30 min and incubated for 10–15 min (up to LPS band appearance) in the revelation mix composed of 300 mL of deionized water (300 mL), 36.5% formaldehyde (0.3 mL, Fluka), and 2.3 M citric acid (0.1 mL). The revelation was stopped by incubating the gels in 10% acetic acid for 5 min. Gels were finally washed in deionized water, numerized with a Samsung PL51 camera, and analyzed by ImageJ software. The percentage of inhibition of LPS heptosylation was defined as the relative area of the R*e*-LPS band compared to the cumulated areas of R*e*-LPS and Core-LPS bands.

### MIC Determination of Antibiotics Combined with a GmhA Inhibitor Against *E. coli* C7 (018:K1:H7)

The compound to be tested was diluted in 50 mM HEPES buffer (pH 7.4) from 10 mM stock solution in DMSO. Five microliters of compound dilution or buffer were distributed in a sterile clear round-bottom 96-well polystyrene microplate (Corning). Five microliters of serial 2-fold dilutions of reference antibiotics [Erythromycin (Fluka), Rifampicin (Fluka)] in DMSO were added. An exponentially growing preculture of *E. coli* C7 (O18:K1:H7) in LB was diluted to 10^4^ cfu/mL and supplemented with 100 μM G6P, and 90 μL of this suspension were added to the microplates. After overnight incubation at 37 °C, the MIC of the antibiotics were determined for each test compound concentration as the lowest antibiotic concentration for which no bacterial pellet is visible without magnification.

### Time-Kill Kinetics of Rifampicin in Combination with Compound **17**

Rifampicin (Fluka) was diluted in DMSO, and the compound to be tested was diluted in water from 50 mM stock solution in 50 mM HEPES buffer, pH 7.4. Ten microliter of DMSO was distributed in sterile 1.5 mL polypropylene microtubes (Eppendorf). 2.5 μL of compound dilution or water, 2.5 μL of Rifampicin or DMSO, 2.5 μL of 10 mM G6P in water (100 μM final concentration), and 230 μL of LB were added to the microtubes. *E. coli* C7 (O18:K1:H7) was grown in 5 mL of LB broth at 37 °C until mid-log phase. The suspension was then diluted in LB to obtain a 100× inoculum of 5 × 10^7^ cfu/mL, 2.5 μL of which were added to each microtube (5 × 10^5^ cfu/mL final inoculum). The microtubes were incubated at 37 °C, and aliquots of 10 μL were sampled at 0, 2, 4, 6, 8, and 24 h for bacterial enumeration in duplicate.

### Engineering of gmhA-Deleted *E. coli* C7

The mutant was obtained by homologous recombination using the lambda Red recombinase system described by Datsenko and Wanner^35^ A chloramphenicol resistance cassette (CamR) was PCR-amplified from a pKD3 plasmid by Pfu DNA polymerase using primers with extensions homologous to the genetic environment upstream and downstream of the gmhA gene (forward primer: 5′-TGTTTACAATATAATTACAACAAAGCT CACATTGTTGC TGGTGTAGGCTGGAGCTGCTTC; reverse primer: 5′-TCAATTAACTGGATCAGGATATGGATCACTTTA ATGTGGACATATGAATATCCTCCTTAG), according to the supplier’s instructions, and purified using QIAquick PCR Purification Kit (Qiagen). *E. coli* C7 containing the curable thermosensitive helper plasmid pKD46 was grown at 30 °C with shaking in 70 mL of LB supplemented with 100 mg/L ampicillin and 1 mM l-arabinose to an OD of 0.5, washed thrice in 70, 35, and 17 mL of ice-cold 10% glycerol, and then resuspended in 180 μL of ice-cold 10% glycerol. Fifty five microliters of cells were electroporated with 5 μL of purified PCR product. The cells were resuspended in SOC medium, incubated for 1 h at 35 °C under shaking, then plated on LB agar containing 10 mg/L chloramphenicol. After overnight growth at 37 °C, isolated colonies were picked and checked for the insertion of the CamR cassette in the gmhA locus by colony PCR (forward primer: 5′-GTGATGATAT GGTTGTAGTGG; reverse primer: 5′-CATCCCGAGCAATTCG CACA). Clones with a PCR product of the expected 1.5 kb size corresponding to gmhA::CamR (vs 1.0 kb for gmhA) were grown three times overnight at 37 °C on nonselective agar, then checked again for chloramphenicol resistance and ampicillin susceptibility for insert stability and loss of the pKD46 thermosensitive plasmid, respectively. Selected clones were suspended in LB 20% glycerol and stored at −80 °C.

### Sensitization to Killing by the Complement of Human Serum

The *E. coli* C7 wild type and *gmhA*-deleted strains were isolated from TSA plates and grown overnight in 10 mL of LB broth at 35 °C. The suspension was then diluted in LB to obtain an inoculum of 5 × 10^5^ cfu/mL in 2 mL, complemented with compound **24** at a final concentration of 0 or 1 mM in 5% DMSO and glucose-6-phosphate at 100 μM. After 5 h of incubation under shaking at 37 °C, the bacterial inoculum was adjusted to 10^5^ cfu/mL in 20% LB broth mixed with 80% human serum (Biopredic) and filtered on 0.45 μm pore size membranes and used as such, or further heated for 30 min at 56 °C for complement deactivation. A volume of 250 μL of each condition was finally incubated at 37 °C in microtubes, and aliquots of 10 μL were sampled at 0, 15, 30, 60, 120, and 180 min for bacterial enumeration in duplicate.

### HepG2 Cytotoxicity

HepG2 cells (LGC Promochem, ref HB-8065) in EMEM medium supplemented with fetal bovine serum (10%), Ampicillin (100 mg/L), and Streptomycin (100 mg/L) were plated on cell culture plates (Costar 3596, 2 × 10^4^ cells/well) and incubated overnight at 37 °C in 5% CO_2_. The culture medium was then aspirated and replaced by supplemented EMEM (210 μL including 2% DMSO) containing dilutions of compounds. Plates were incubated overnight at 37 °C in 5% CO_2_. Cell viability was determined by the Celltiter Aqueous One Cell Proliferation Assay (G3581–80, Promega) according to the manufacturer’s recommendations, using a Multiskan absorption reader (490 nm).

## Data Availability

Authors will release the atomic coordinates and experimental data upon article publication.

## Supporting Information Available

General methods, synthetic procedures for compounds **17**, **20**, **24**, **25**, **27**, **31,** and **96**, HILIC reaction control of the final deprotection and stacked ^1^H NMR plot of individual fractions of compound **17**, ^1^H, ^13^C, and ^31^P NMR spectra of compounds **17**, **20**, **24**, **25**, **27**, **31** and **96**, 1D NOE-difference spectrum of compound **17** with selective saturation of one formyl proton, synthetic procedure reference for compounds **41**, **54**, **68**, **76** and **84**, X-ray data collection and refinement statistics, role of UhpT phospho-sugar transporter in the transport of d-*glycero*-d-*manno*-heptose 7-phosphate (H7P), IC_50_ fits of *E. coli* GmhA in biochemical inhibition assays, and EC_50_ fits of LPS biosynthesis inhibition assays (PDF)
Molecular formula strings (CSV)

## Accession Codes

**17**: 8V4J. **84**: 8V2T.

## Abbreviations

ADP: Adenosine diphosphate
ASU: Asymmetric unit
CDI: Carbonyldiimidazole
CPS: Capsular Polysaccharide
DMP: Dess-Martin periodinane
Fbas: Fructose bis-phosphate aldolase
Gmh: *Glycero*-*manno*-heptose
HEPES: 2-[4-(2-Hydroxyethyl)-1-piperazinyl]ethanesulfonic acid
HILIC: Hydrophilic interaction liquid chromatography
MR: Molecular replacement
PDF: Peptide deformylase
S-Layer: Surface-Layer
TEA: Triethylamine
