# Supplementary material for: Potentiating Activity of GmhA Inhibitors on Gram-Negative Bacteria
Source: J Med Chem. 2024 Apr 10;67(8):6610–23. doi: 10.1021/acs.jmedchem.4c00037 (PMC11056994; doi:10.1021/acs.jmedchem.4c00037)

Supporting Information

Potentiating Activity of GmhA Inhibitors on Gram-negative Bacteria

François Moreau,[a] Dmytro Atamanyuk,[a,b] Markus Blaukopf,[c] Marek Barath,[c,d] Mihály Herczeg,[c,e] , Nuno M. Xavier, [c,f] Jérôme Monbrun,[g,j] Etienne Airiau,[g] Vivien Henryon,[g] Frédéric Leroy,[h,k] Stéphanie Floquet, [a] Damien Bonnard, [a] Robert Szabla[i], Chris Brown[i], Murray S. Junop[i], Paul Kosma[c] and Vincent Gerusz*[a,l]*

[a] Mutabilis, 102 Avenue Gaston Roussel, 93230 Romainville, France

[b] Present address: AB-Science, 3 Avenue George V, 75008 Paris, France

[c] Department of Chemistry, University of Natural Resources and Life Sciences, Muthgasse 18, A-1190 Vienna, Austria

[d] Institute of Chemistry, Center for Glycomics, Slovak Academy of Sciences, Dúbravská cesta 9 SK-845 38 Bratislava, Slovakia

[e] Department of Pharmaceutical Chemistry, University of Debrecen, Hungary

[f] Centro de Química Estrutural, Institute of Molecular Sciences, Faculdade de Ciências, Universidade de Lisboa, Ed. C8, 5° Piso, Campo Grande, 1749-016 Lisboa, Portugal

[g] Activation, 10 Rue Jacquard, 69680 Chassieu, France

[h] Carbosynth Limited, 8&9 Old Station Business Park, Compton, Berkshire, RG20 6NE, United Kingdom

[i] University of Western Ontario, Dept. of Biochemistry, London ON, Canada

[j] Present address: DRT 30 Rue Gambetta 40100 Dax, France

[k] Present address: Novartis 20 Rue Rudolf Diesel, 01630 Saint-Genis-Pouilly, France

[l] Present address: Debiopharm, Rue du Levant 146, CP368, 1920 Martigny, Switzerland

Table of Contents

General Methods S2

Synthetic procedures for compounds **17**, **20**, **24**, **25**, **27**, **31** and **96** S3

Fig. S1. HILIC reaction control of the final deprotection and stacked ^{1}H NMR plot of individual fractions of compound **17**. S8

^{1}H, ^{13}C and ^{31}P NMR spectra of compounds **17**, **20**, **24**, **25**, **27**, **31** and **96** S19

Fig. S2. 1D NOe-difference spectrum of compound **17** with selective saturation of one formyl proton

Synthetic procedures reference for compounds **41**, **54**, **68**, **76** and **84** S20

Table S1. X-ray data collection and refinement statistics S31

Fig. S3. Role of UhpT phospho-sugar transporter in the transport of D-*glycero*-D-*manno*-heptose 7-phosphate (H7P) S34

Fig. S4: IC_{50} fits of *E. coli* GmhA in biochemical inhibition assays S35

Fig. S5: EC_{50} fits of LPS biosynthesis inhibition assays S36

Experimental section

General methods

All purchased chemicals were used without further purification unless stated otherwise. Solvents were dried over activated 4 Å (CH_2Cl_2, pyridine, THF, DMF) and 3 Å (CH_3CN) molecular sieves. Cation exchange resin DOWEX 50 H^+ was regenerated by consecutive washing with HCl (3 M), water and dry MeOH. Aqueous solutions of salts were saturated unless stated otherwise. Concentration of organic solutions was performed under reduced pressure < 40 °C. Optical rotations were measured with a Perkin-Elmer 243 B or Anton Paar MCP100 Polarimeter. Thin layer chromatography was performed on Merck precoated plates: generally on 5 x 10 cm, layer thickness 0.25 mm, Silica Gel $60F_{254}$; alternatively on HPTLC plates with 2.5 cm concentration zone (Merck). Spots were detected by dipping reagent (anisaldehyde-H_2SO_4) followed by heating. For column chromatography silica gel (0.040 – 0.063 mm) was used. HP-column chromatography was performed on pre-packed columns (YMC-Pack SIL-06, 0.005 mm, 25 x 1 cm and 25 x 2 cm). HPLC (analytical and preparative) was performed on an Agilent 1100 HPLC with diode array detection. Preparative HPLC was performed at 0.7 mL/min on a Thermo Electron, Hypersil BDS C-18 column (250 x 4.6 mm, 5 µm) using a gradient of acetonitrile and water with 0.1% TFA (50% in acetonitrile to 100% and then back to 50%). The tested compounds were determined to be >95% pure via HPLC.
NMR spectra were recorded with a Bruker Avance III 600 instrument (600.22 MHz for ^1H, 150.93 MHz for ^{13}C, 242.9 MHz for ^{31}P) or a Bruker Avance 300 instrument using standard Bruker NMR software (Topspin3.6.2). ^1H spectra were referenced to 7.26 ($CDCl_3$), 3.34 (MeOD) and 0.00 (D_2O, external calibration to 2,2-dimethyl-2-silapentane-5-sulfonic acid) ppm unless stated otherwise. ^{13}C NMR spectra were referenced to 77.00 ($CDCl_3$), 49.00 (MeOD) and 67.40 (D_2O, external calibration to 1,4-dioxane) ppm. ^{31}P NMR spectra were referenced to external ortho-phosphoric acid (δ 0.0) for solutions in D_2O. Assignments were based on COSY, HSQC, HMBC and TOCSY data. ESI-MS data were obtained on a Micromass Q-TOF Ultima Global instrument.

General procedure for the HILIC based monitoring of final deprotection steps:

Reactions were monitored on a ZIC HILIC (Merck 4.6x 150 mm (CatNo 1.50444.001) analytical HPLC column attached to a Shimadzu LC 10 HPLC system equipped with an Alltech 3300 ELSD and a Shimadzu LC MS 2020 Detector. Acetonitrile/water, both using 0.1% (v/v) as modifier for LCMS was used as solvent system. The gradient employed at a flow rate of 0.75 ml*min^{-1} a gradient starting from 95% acetonitrile which went after a 2 min plateau at 95% down to 40% acetonitrile over 15 minutes, after which a 40% acetonitrile plateaued was kept for 7 minutes before returning to the

initial 95% acetonitrile concentration. Deprotected compounds usually eluted in or close to the 40% acetonitrile plateau.

General procedure for the purification of compounds via HILIC chromatography:
After final deprotection the compound was dissolved in H_2O and made neutral by adding $NaHCO_3$. The solution was passed over a short plug of HILIC material (ZIC HILIC SPE SeQuant 500 mg) using 2:1 MeCN-H_2O as eluant. Product containing fractions were determined by TLC, concentrated and lyophilized. The residue was taken up in acetonitrile and water was added slowly until a clear solution for injection was obtained. The sample was injected via a sample loop into a semi preparative SeQuant ZIC HILIC column (Merck, 25x1 cm, Cat No 1.50494.0001) which was connected to a HPLC system equipped with a binary pump system and a fraction collector. The column was equilibrated with acetonitrile/water 85/15. A gradient from 85% to 40% acetonitrile at a flow rate of 3 ml/m over 7 column volumes followed by a short plateau of 1.5 column volumes at 40% acetonitrile with a final backwashing phase to 85% acetonitrile was employed. Fractions were collected at a size of 2 ml. TLC positive (staining with phosphor molybdate) fractions were individually concentrated and freeze dried (SpeedVac) and each fraction was individually checked by ^{1}H NMR. Pure product fractions were combined and lyophilized.

2,3,4-Tri-*O*-benzyl-5-*O*-triisopropylsilyl-D-ribose diethyldithioacetal (**11**). Compound **10** (300 mg, 0.57 mmol) was dissolved in dry pyridine (2 mL). DMAP (70 mg, 0.57 mmol) and triisopropylsilyl chloride (183 µL, 0.85 mmol) were added and the reaction mixture was stirred at room temperature for 5 h. Additional amounts of DMAP (70 mg, 0.57 mml) and TIPSCl (183 µL, 0.85 mmol) was added and stirring continued for 9 h. The reaction mixture was diluted and co-evaporated with toluene. The residue was dissolved in Et_2O (15 mL) and washed with satd aqu $NaHCO_3$. The organic phase was dried ($MgSO_4$), concentrated, and purified *via* column chromatography on silica gel (toluene-hexane 2:1→5:1) to give **11** (356 mg, 93%) as colorless oil; $[\alpha]_D^{20}$ +20.7 (*c* 1.0, $CHCl_3$); ^{1}H NMR (300 MHz, $CDCl_3$): δ 7.36-7.22 (15 H, Ar-H), 4.98-4.57 (m, 6 H, 3 x OC*H₂*Ph), 4.26 (d, 1 H, $J_{1,2}$ = 3.3 Hz, H-1), 4.13 (dd, 1 H, $J_{3,2}$ = 7.5 Hz, $J_{3,4}$ = 1.9 Hz, H-3), 4.05 (dd, 1 H, $J_{2,1}$ = 3.3 Hz, $J_{2,3}$ = 7.5 Hz, H-2), 3.99-3.91 (m, 2 H, H-4, H-5a), 3.89-3.79 (m, 1 H, H-5b), 2.70-2.55 (m, 4 H, 2 x SCH_2CH₃), 1.24-1.13 (m, 6 H, 2 x SCH_2CH_3), 1.05-0.95 [m, 21 H, $Si(CHMe_2)_3$]. ^{13}C NMR (150 MHz, $CDCl_3$): δ 139.00, 138.62, 138.51 (3 x Ar-C_q), 128.25, 128.15, 128.11, 127.82, 127.68, 127.47, 127.80, 127.28,127.26 (Ar-C), 82.78 (C-2), 81.04 (C-4), 78.81 (C-3), 74.75, 73.15, 72.93 (3 x O*C*H_2Ph), 64.10 (C-5), 54.14 (C-1), 26.21, 24.95 (2 x S*C*H_2CH_3), 18.02 [$Si(CH(CH_3)_2)_3$], 14.46, 14.44 (2 x SCH_2CH_3), and 11.91 [$Si(CH(CH_3)_2)_3$].ESI-HRMS: *m/z* calcd for $C_{39}H_{58}O_4S_2Si$ $[M+Na^+]^+$: 705.3438; found: 705.3437.

2,3,4-Tri-*O*-benzyl-5-*O*-triisopropylsilyl-D-ribose (12). NBS (464 mg, 2.6 mmol) was added at 0 °C to a solution of **11** (356 mg, 0.52 mmol) in a 5:1 mixture of acetone-water (12 mL). The reaction mixture was stirred for 30 min at 0 °C. Satd aqu $NaHCO_3$ (5 mL) followed by addition of satd aq $Na_2S_2O_3$ (5 mL) and stirring was continued for 15 min. The solution was concentrated, and the remaining aqueous phase was extracted with Et_2O (2 x 10 mL). The combined organic phases were dried ($MgSO_4$) and concentrated. The residue was purified by silica gel chromatography (toluene-hexane 1:1→ toluene) to give **12** (292 mg, 97%) as colorless syrup: $[\alpha]_D^{20}$ +12.2 (*c* 1.5, $CHCl_3$); ^{1}H NMR (300 MHz $CDCl_3$): δ 9.44 (d, 1 H, J = 0.9 Hz, CHO), 7.36-7.24 (m, 15 H, Ar-H), 4.85-4.47 (m, 6 H, 3 x OCH_2Ph), 4.10 (dd, 1 H, $J_{2,3}$ = 2.1 Hz, H-2), 4.03-3.91 (m, 2 H, H-3, H-5a), 3.84-3.76 (m, 2 H, H-4, H-5b), 1.13-0.96 [m, 21 H, Si(CHMe$_2$)$_3$]. ^{13}C NMR (75 MHz $CDCl_3$): δ 201.41 (HC=O), 138.27, 137.77, 137.59 (Ar-C_q), 128.46, 128.39, 128.21, 127.94, 127.89, 127.85, 127.80, 127.44 (Ar-C), 82.60 (C-2), 80.87 (C-3), 78.63 (C-4), 73.17, 73.11, 72.74 (3 x OCH_2Ph), 63.53 (C-5), 18.04,18.03 [Si(CH(CH_3)$_2$)$_3$] and 11.95 8 [Si(CH(CH$_3$)$_2$)$_3$]. ESI-MS: *m/z* calcd for $C_{35}H_{48}O_5Si$ [M+H$^+$]$^+$: 577.33; found 577.45.

1-*N*-Benzyloxyamino-2,3,4-tri-*O*-benzyl-1-deoxy-5-*O*-triisopropylsilyl-D-ribitol (13). Compound **12** (285 mg, 0.49 mmol) and *O*-benzylhydroxylamine (86 µL, 0.74 mmol) were stirred at 60 °C in 1:6.5 pyridine-MeOH (6 mL) overnight. An additional portion of *O*-benzylhydroxylamine (20 µL) was added and stirring continued for 12 h. The solution was concentrated, and the residue was subjected to flash-chromatography over a short plug of silica gel (2 g, hexane-EtOAc 10:1) to give the crude oxime (317 mg, 0.47 mol, 96%) as colorless oil. An aliquot of the oxime (150 mg, 0.22 mmol) was dissolved in acetic acid (1.5 ml) followed by portionwise addition of sodium cyanoborohydride (124 mg, 1.98 mmol) and stirring for 20 min at room temperature. The reaction mixture was diluted with water and twice extracted with dichloromethane. The organic phase was made neutral by addition of satd aq $NaHCO_3$ followed by drying ($MgSO_4$) and removal of the solvent. The residue was purified by HPLC (hexane-EtOAc 15:1) to give **13** (123 mg, 75% over two steps) as colorless oil; $[\alpha]_D^{20}$ -25.8 (*c* 1.1, $CHCl_3$); ^{1}H NMR (300 MHz, $CDCl_3$): δ 7.33-7.21 (20 H, Ar-H), 5.92 (bs, 1 H, NH), 4.80-4.48 (m, 8 H, 4 x OCH_2Ph), 4.12 (dt, 1 H, $J_{1b,2}$ = 8.0 Hz, $J_{1a,2}$ = 3.3 Hz, H-2), 4.00 (dd, 1 H, $J_{5a,5b}$ = 10.7 Hz, $J_{5a,4}$ = 3.7 Hz, H-5a), 3.88 (dd, 1 H, $J_{3,2}$ = 3.4 Hz, $J_{3,4}$ = 6.1 Hz, H-3), 3.83 (dd, 1 H, $J_{5b,4}$ = 6.0 Hz, H-5b), 3.62 (dt, 1 H, H-4), 3.27 (dd, 1 H, $J_{1a,1b}$ = 13.7 Hz, $J_{1a,2}$ = 3.3 Hz, H-1a), 3.11 (dd, 1 H, $J_{1b,2}$ = 8.1 Hz, H-1b), 1.14-0.96 [m, 21 H, Si[CH(CH$_3$)$_2$]$_3$]. ESI-HRMS: *m/z* calcd for $C_{42}H_{57}NO_5Si$ [M+H$^+$]$^+$ 684.4079; found 684.4078.

1-*N*-Benzyloxy-*N*-formylamino-2,3,4-tri-*O*-benzyl-1-deoxy-5-*O*-triisopropylsilyl-D-ribitol (14) Formic acid (41 µL, 1.08 mmol) was added to a solution of carbonyl diimidazole (CDI) (175 mg, 1.0 mmol) in THF (2 mL) at 0 °C was added and stirred at 0 °C for 30 min. Then a solution of **13** (148 mg, 216 µmol) in THF (1 mL) was added and the reaction mixture was stirred at 0 °C for 1 h. The reaction

mixture was concentrated, and the residue was purified on silica gel (hexane-EtOAc 3:1) to give **14** (146 mg, 94 %) as colorless oil; $[\alpha]_D^{20}$ -5.4 (*c* 0.9, $CHCl_3$). ^1H NMR (600 MHz, toluene-d_8, 80 °C): δ 8.07 (bs, 1 H, NCHO), 7.33-7.02 (20 H, Ar-H), 4.79-4.55 (m, 8 H, 4 x OCH_2Ph), 4.23-4.19 (m, 1 H, H-2), 4.03 (dd, 1 H, $J_{5a,5b}$ = 10.6 Hz, $J_{5a,4}$ = 3.7 Hz, H-5a), 3.93-3.88 (m, 2 H, H-3, H-5b), 3.82-3.71 (m, 3 H, H-4, H-1a, H-1b), 1.08-1.03 [m, 21 H, Si[CH($CH_3)_2)_3$]. ^{13}C NMR (150 MHz, toluene-$_{d6}$, 80 °C, HSQC data): δ 139.54, 139.33, 139.12 (Ar-C_q, overlapped with toluene signals), 129.56, 129.27, 128.74, 128.70, 128.60, 128.55, 128.36, 128.32, 127.84, 127.80 (Ar-C), 81.30 (C-4), 80.10 (C-3), 77.23 (C-2), 77.33 (NO*C*H_2Ph), 73.98, 73.83, 72.29 (3 x O*C*H_2Ph), 64.87 (C-5), 48.94 (C-1), 18.42 [Si(CH(*C*$H_3)_2)_3$], 12.76 [Si($CHMe_2)_3$]. ESI-HRMS: *m/z* calcd for $C_{43}H_{57}NO_6Si$ $[M+H^+]^+$ 712.4028; found 712.4037.

1-*N*-Benzyloxy-*N*-formylamino-2,3,4-tri-*O*-benzyl-1-deoxy-D-ribitol (15) Compound **14** (146 mg, 0.20 mmol) was dissolved in dry THF (1 mL) and TBAF (308 µl of a 1 M solution in THF) was added. The mixture was stirred at rt for 30 minutes when TLC showed complete conversion. The reaction mixture was concentrated, and the residue dissolved in EtOAc, filtered through a short plug of silica gel (2 g) and concentrated. Purification by HPLC (hexane-EtOAc 2:1→1:1) gave **15** as colorless syrup (100 mg, 87%). $[\alpha]_D^{20}$ +44 (*c* 0.5, $CHCl_3$), ^1H NMR (600 MHz, toluene- d_8, 80 °C): δ 8.06 (bs, 1 H, NCHO), 7.25-7.02 (20 H, Ar-H), 4.65-4.40 (m, 8 H, 4 x OC*H_2*Ph), 4.12-4.08 (m, 1 H, H-2), 3.81 (dd, 1 H, *J* = 6.5 Hz, *J* = 2.5 Hz, H-3), 3.75-3.62 (m, 4 H, H-5a, H-5b, H-1a,H-1b), 3.60-3.56 (m, 1 H, H-4), ^{13}C NMR (150 MHz, toluene- d_8, 80°C): δ 129.19, 128.91, 128.39, 128.36, 128.34, 128.26, 128.10, 128.00, 127.96 (Ar-C), 79.92 (C-4), 79.84 (C-3), 77.11 (NO*C*H_2Ph), 76.94 (C-2*), 73.96, 72.91, 72.56 (3 x O*C*H_2Ph), 61.52 (C-5), 48.66* (C-1). *determined by HSQC. ESI-HRMS: *m/z* calcd for $C_{34}H_{37}NO_6$ $[M+H^+]^+$ 556.2694; found 556.2702.

1-*N*-Benzyloxy-*N*-formylamino-2,3,4-tri-*O*-benzyl-5-dibenzyloxyphosphoryl-1-deoxy-D-ribitol (16). Compound **15** (100 mg, 0.18 mmol) was twice evaporated with toluene and the residue was dried under vacuum for 12 h. Dibenzyl *N*,*N*-diisopropylaminophosphoramidite (91 µL, 0.27 mmol) was added to a solution of **15** in anhydrous DCM (1.5 mL) followed by slow addition of 1*H*-tetrazole (0.45 M in MeCN, 600 µL, 0.27 mmol) at rt. The solution was stirred for 40 min and then cooled to -78 °C. A solution of *m*CPBA (70%, 71 mg, 0.28 mmol) in DCM (1 mL) was added slowly to the reaction mixture and stirred for 20 min at -78 °C. Triethylamine (40 µL, 0.28 mmol) was added and the reaction mixture was allowed to warm to rt. The reaction mixture was diluted with DCM (10 mL), washed with sat aqu $NaHCO_3$, dried ($MgSO_4$) and concentrated. Purification of the residue on silica gel (hexane-EtOAc 1:2) followed by HPLC (hexane-EtOAc 2:1 → 1:1) gave **16** as colorless oil (137 mg, 93%); $[\alpha]_D^{20}$ -44 (*c* 0.5, $CHCl_3$), ^1H NMR (600 MHz, toluene- d_8, 80°C): δ 8.04 (bs, 1 H, NCHO), 7.33-6.99 (30 H, Ar-H), 4.95-4.87 (m, 2 H, P(O)*C*H_2Ph), 4.66-4.41 (m, 9 H, 4 x OC*H_2*Ph, H-5a), 4.25-4.20 (m, 1 H, H-5b), 4.12-4.09 (m, 1 H,

H-2), 3.84-3.79 (m, 2 H, H-3, H-4), 3.75-3.58 (m, 2 H, CH_2N). ^{13}C NMR (150 MHz, toluene- d_8, 80°C): δ 129.59, 129.28, 128.77, 128.68,128.65, 128.63, 128.53, 128.50, 128.36 (Ar-C), 79.56 (C-3), 78.86 ($J_{P;C}$ = 6.2 Hz, C-4), 77.45 (NO*C*H_2Ph), 77.17 (C-2), 74.17, 73.33, 73.19 (3 x OCH_2Ph), 69.48 (2 x P(O)*C*H_2Ph), 67.05 ($J_{P,C}$ = 5.4, C-5), 49.63* (C-1).*determined by HSQC, ESI-HRMS: *m/z* calcd for $C_{48}H_{50}NO_9P$ [M+Na$^+$]$^+$ 838.3115; found 838.3116.

1-*N*-Formyl-*N*-hydroxyamino-1-deoxy-5-phosphoryl-D-ribitol monosodium salt (17) $Pd(OH)_2$ (10 mg) was added to a solution of compound **16** (50 mg, 61 µmol) in 5:1:1 THF-H_2O-AcOH (5 mL,) and stirred under H_2 atmosphere at rt for 10.5 h. The progress of the reaction was monitored by HPLC-MS/ELS (Sequant ZIC HILIC 4.6 x 150 mm, gradient MeCN-H_2O 85 → 40 % MeCN). The suspension was filtered through a syringe filter, the syringe filter was washed with 1:1 H_2O-MeOH (10 mL), 1:1 H_2O-THF (10 mL) and the combined filtrates were concentrated. The residue was dissolved in H_2O and made neutral by adding $NaHCO_3$ (11 mg, 0.1 mmol). The solution was passed over a short plug of ZIC-HILIC silica gel (500 mg) using 2:1 MeCN-H_2O as eluant. Product containing fractions were concentrated and lyophilized. The residue (21 mg) was redissolved in 2:1 MeCN-H_2O (300 µL) and purified by repeated ZIC-HILIC chromatography (Sequant ZIC HILIC 10 x 250 mm, gradient MeCN-H_2O 85 → 40%). Fractions were concentrated, individually checked by ^1H NMR analysis and pooled accordingly (Fig.S1 Supporting Information). Lyophilization of the combined fractions gave **17** (5.2 mg, 30%) as colorless solid; $[\alpha]_D^{20}$ -15 (*c* 0.5, H_2O), ^1H NMR (600 MHz, D_2O, rotameric mixture): δ 8.33 (NCHO minor), 7.90 (NCHO major), 4.17-4.13 (m, 1 H, H-2), 3.98-3.89 (m, 2 H, H-5a, H-5b), 3.84-3.80 (m, 1 H, H-4), 3.77 (dd, 1 H, $J_{3,4}$ = 7.2 Hz, $J_{3,2}$ = 5.2 Hz, H-3), 3.71 (dd, 1 H, $J_{1a,1b}$ = 14.9 Hz, $J_{1a,2}$ = 2.8 Hz, H-1a), 3.66 (dd, 1 H, $J_{1b,2}$ = 8.8 Hz, H-1b), ^{13}C NMR (150 MHz, D_2O): δ 165.11* (N**C**HO minor), 161.11* (N**C**HO, major), 73.10 (C-3), 71.91 ($J_{C,P}$ = 6.3 Hz, C-4), 68.08 (C-2), 65.71 ($J_{C,P}$ = 4.6 Hz, C-5), 53.60 (C-1), ^{31}P NMR (242 MHz, D_2O): δ 3.67; ESI-HRMS: *m/z* calcd for $C_6H_{14}NO_9P$ [M-H]$^-$ 274.0333; found 274.0337.

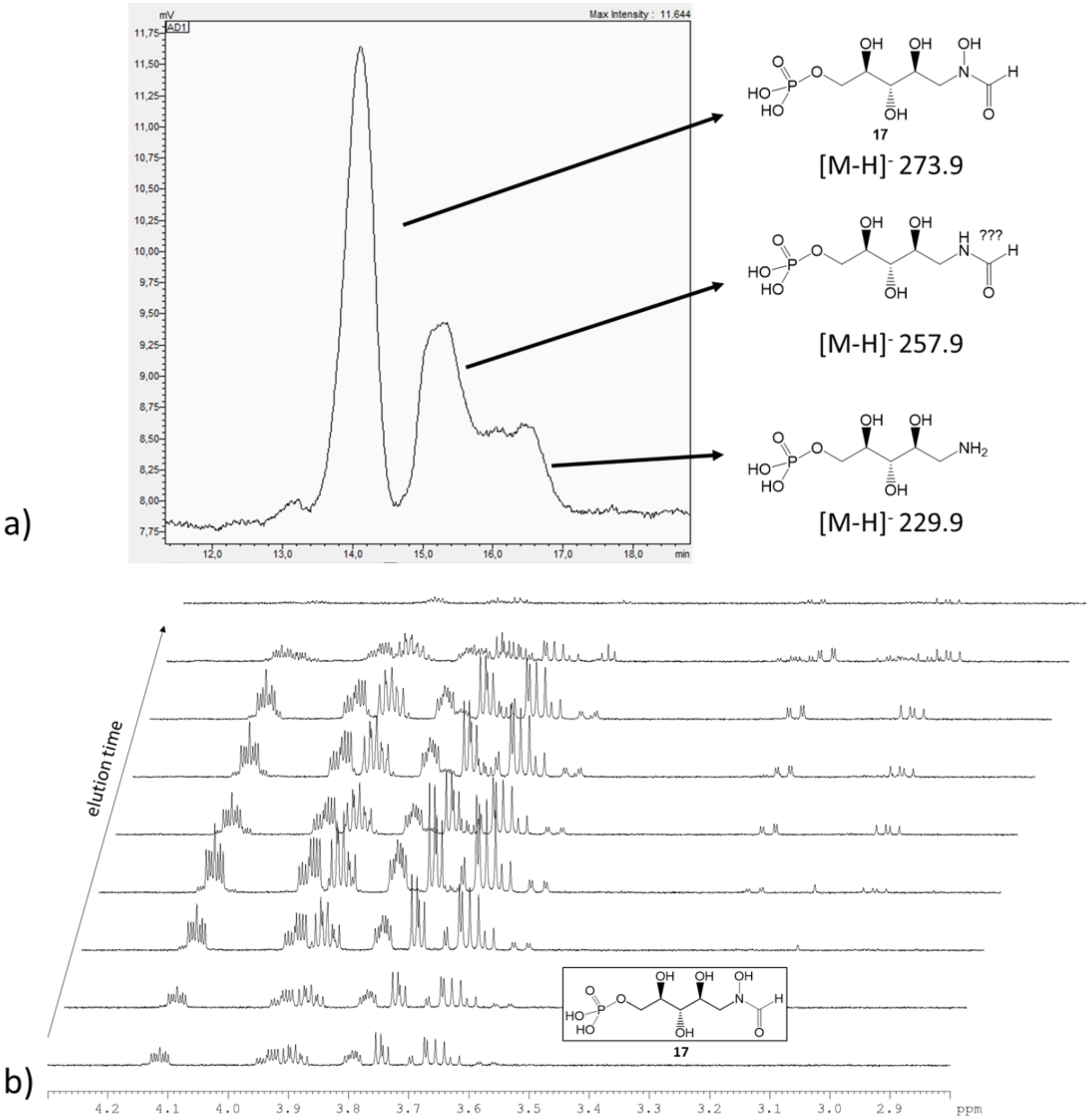

Fig. S1. a) ELSD track of a typical analytical HILIC reaction control of the final deprotection; b) stacked ^{1}H NMR plot of individual fractions of a preparative HILIC purification of compound **17**.

2,3,4-Tri-*O*-benzyl-1-(*N*-benzyloxy-*N*-formylamino)-1,5-dideoxy-5-iodo-D-ribitol (18). A solution of imidazole (44 mg, 0.65 mmol), triphenylphosphine (142 mg, 0.54 mmol), I_2 (137 mg, 0.54 mmol) and compound **15** (250 mg, 0.45 mmol) in anhydrous toluene (5 mL) was stirred under argon at 80 °C for 1 h. The dark yellow reaction mixture was cooled to rt and concentrated. The residue was taken up in DCM (10 mL), filtered through cotton, washed with 5% aq $Na_2S_2O_3$ (2 x 5 mL) and $NaHCO_3$ (5 mL). The aqueous phases were reextracted with DCM (10 mL) and the combined organic phases were dried ($MgSO_4$) and filtered. Silica gel (3 g) was added to the filtrate and the solvent was removed. The remaining silica gel was directly applied onto a prepacked silica gel column. Elution with 2:1 hexane-EtOAc afforded the target compound **18** (240 mg, 66%) as colorless oil; R_f 0.6 (hexane-EtOAc 2:1);

$[\alpha]_D^{20}$ -12 (*c* 0.5, $CHCl_3$). ^{1}H NMR (600 MHz, toluene-d_8, 85 °C) δ: 8.03 (s, 1 H, NCHO), 7.28-6.92 (m, 20 H, Ar-H), 4.67-4.27 (m, 8 H, 4 x O*CH₂*Ph), 4.09 (m, 1 H, H-2), 3.76 (m, 1 H, H-3), 3.72-3.55 (2 x bs, 2 H, H-1a, H-1b), 3.37 (dd, 1 H, $J_{5a,5b}$ = 10.7 Hz, $J_{5a,4}$ = 4.0 Hz, H-5a), 3.28 (m, 1 H, H-4), 3.21 (dd, 1 H, $J_{5b,5a}$ = 10.6 Hz, $J_{5b,4}$ = 3.7 Hz, H-5b). ^{13}C NMR (150 MHz, toluene-d_8, 85°C): δ 138.95, 138.87, 138.50 (Ar-C*q*), 136.01 (C*q*, $NOCH_2Ph$), 129.57-128.00 (Ar-C), 81.78 (C-3), 78.06 (C-4), 77.52 (O*C*H_2Ph), 77.12 (C-2), 74.52, 73.36, 72.72 (3 x O*C*H_2Ph), 8.25 (C-5). ESI-HRMS: *m/z* calcd for $C_{34}H_{36}INO_5$ $[M+H^+]^+$ 666.1711; found 666.1716.

Diethyl [2,3,4-tri-*O*-benzyl-1-(*N*-benzyloxy-*N*-formylamino)-1,5-dideoxy-D-ribitol] 5-phosphonate (19) A solution of compound **18** (222 mg, 0.33 mol) and $P(OEt)_3$ (300 µL) was heated to 150 °C for 3 h. The reaction mixture was cooled to rt and the residual $P(OEt)_3$ was removed *in vacuo*. The residual oil was chromatographed on silica gel (2:1 hexane-EtOAc). The product containing fractions were pooled, concentrated, and purified by HPLC (2:1 hexane-EtOAc) to give **19** as colorless oil (100 mg, 44%); R_f 0.26 (2:1 hexane-EtOAc); $[\alpha]_D^{20}$ +0.5 (c 0.9, $CHCl_3$); ^{1}H NMR (DMSO, 600 MHz, 90°C): δ 8.14 (bs, 1 H, NCHO), 7.0-7.24 (m, 20 H, Ar-H), 4.92-4.89 (m, 2 H, NO*CH₂*Ph), 4.74-4.56 (m, 6 H, 2 x O*CH₂*Ph), 4.14-4.08 (m, 1 H, H-4), 4.03-3.95 (m, 6 H, H-2, H-3, 2 x O*CH₂*CH_3), 3.88-3.80 (m, 2 H, H-1a, H-1b), 2.23 (ddd, 1 H, $J_{5a,5b}$ = 15.8 Hz, $J_{5a,4}$ = 4.4 Hz, $J_{5a,P}$ = 18.4 Hz, H-5a), 2.15 (ddd, 1 H, $J_{5b,5a}$ = 15.8 Hz, $J_{5b,4}$ = 7.4 Hz, $J_{5b,P}$ = 17.6 Hz, H-5b), 1.24-1.20 (m, 6 H, 2 x OCH_2*CH₃*); ^{13}C NMR (DMSO, 150 MHz, 90 °C): δ 137.64, 137.52, 137.37 (C*q*, 3 x C-Ar), 134.38 (C*q*, $NOCH_2Ph$), 128.92, 128.88, 128.26, 127.67, 127.51, 127.39, 127.31, 127.26, 126.85, 126.83, 126.79, 126.64, 126.61, 126.54 (Ar-C), 79.88 (d, $J_{C4,P}$ = 10.3 Hz, C-3), 75.37 (O*C*H_2Ph), 74.72 (C-2), 74.15 (C-4), 71.94, 71.10, 71.05 (O*C*H_2Ph), 60.25 (d, $J_{P,OCH2CH3}$ = 6.0 Hz, PO*C*H_2CH_3), 60.15 (d, $J_{P,OCH2CH3}$ = 6.0 Hz, PO*C*H_2CH_3), 46.82* (*det by HSQC, NCH_2), 27.02 (d, $J_{P,C\text{-}5}$ = 139 Hz, C-5), 15.31, 15.27, 15.24 (2 x OCH_2*C*H_3); ^{31}P NMR (DMSO, 242 MHz, 90 °C): δ 28.78. ESI-HRMS: *m/z* calcd for $C_{38}H_{46}NO_8P$ $[M+Na^+]^+$ 698.2853; found 698.2582.

Sodium 1-(*N*-benzyloxy-*N*-formylamino)-1,5-dideoxy-D-ribitol] 5-phosphonate (20) Compound **19** (44.5 mg, 64 µmol) was dissolved in anhydrous DCM, pyridine (21 µL, 0.26 mmol) and TMSBr (34 µl, 0.26 mmol) were added under argon atmosphere, and the reaction was stirred at room temperature for 29 h. The reaction was monitored by HPLC MS (C-4, gradient 95:5 H_2O-MeCN 5 → MeCN). Upon full conversion to the product the reaction mixture was concentrated, taken up in DCM (10 mL) and washed with citric acid (0.25 M). The aqueous phase was reextracted with DCM. The organic phase was dried ($MgSO_4$) and concentrated to give a white solid (36 mg) that was dissolved in 5:1 THF-H_2O containing 1% AcOH (2 mL). $Pd(OH)_2$-C (6.2 mg) was added and the reaction mixture was stirred under hydrogen atmosphere (1 bar) at rt for 7 h when fresh catalyst (6.2 mg) was added and stirring was

continued for further 15 h for completion. The reaction was monitored by HPLC-MS (Sequant ZIC HILIC 4.6 x 150 mm, Gradient 15:85 MeCN-H_2O → 4:6 MeCN-H_2O, R_t of product 13.5 min). The reaction mixture was filtered through a syringe filter and the filter was successively washed with 1:1 MeOH-H_2O (15 mL). The filtrate was concentrated, and the residue was dissolved in H_2O, neutralized with $NaHCO_3$ (10 mg, 0.12 mmol) and concentrated. The residue was passed (1: 1 MeCN-H_2O) through a short plug of ZIC-HILIC silica gel (500 mg). Product containing fractions were concentrated, dissolved in 3:1 MeCN-H_2O (200 µL) and purified by repeated ZIC-HILIC chromatography (Column: Sequant ZIC HILIC 10 x 250 mm, Merck, gradient 15:85 MeCN-H_2O → 4:6 MeCN-H_2O, flow rate 3 mL, fraction size 2 mL). Fractions were concentrated and pooled according to ^1H NMR analysis (Fig.S2 Supporting Information) to give the target compound **20** (4 mg, 23 %) as white solid. $[\alpha]_D^{20}$ -6.7 (*c* 0.4, H_2O); ^1H NMR (600 MHz, D_2O): δ 8.33 (s, 0.2 H, NC(=O)H, minor rotamer), 7.89 (s, 0.8 H, NC(=O)H, major rotamer), 4.07-4.02 (m, 1 H, H-4), 4.02-3.98 (dt, 1 H, H-3), 3.75 (dd, 1 H, $J_{1a,1b}$ 14.9, $J_{1a,2}$ 2.2 Hz, H-1a), 3.67-3.61 (m, 2 H, H-2, H-1b), 1.83 (ddd, 1 H, $J_{5a,5b}$ 14.9, $J_{5a,4}$ 3.7, $J_{5a,P}$ 17.8 Hz, H-5a), 1.61 (ddd, 1 H, $J_{5b,5a}$ 15.0, $J_{5b,4}$ 9.4, $J_{5b,P}$ 15.1 Hz, H-5b).^{31}P NMR (D_2O, 242 MHz): δ 20.53 ($P(O)(OH)_2$). ^{13}C NMR (D_2O, 150 MHz): δ 161.08 (CHO), 76.72 (d, *J* 11.9 Hz, C-3), 69.19 (d, *J* 3.3 Hz, C-4), 68.44 (C-2, minor rotamer), 67.76 (C-2, major rotamer), 54.12 (C-1, major rotamer), 50.07 (C-1, minor rotamer), 31.53 (d, *J* 128.39 Hz, C-5); ESI-MS: *m/z* calcd. for $C_6H_{14}NO_8P$ $[M-H]^-$: 258.0384, found 258.0383.

2,3,4-Tri-*O*-benzyl-1-(*N*-benzyloxy-*N*-formylamino)-1-deoxy-5-oxo-D-ribitol (21). Dess-Martin reagent (200 mg, 0.46 mmol) was added to a solution of **15** (170 mg, 0.31 mmol) in DCM (4 mL). The reaction was stirred at rt for 1 h, then diluted with DCM and washed sequentially with satd aq $NaHCO_3$ and $Na_2S_2O_3$. The organic phase was washed with brine, dried and concentrated to give the crude product, which was stored under Ar atmosphere at -78 °C. Yield: 168 mg, (quant.) of aldehyde **21** as a colorless syrup; $[\alpha]_D^{20}$ +4.9 (*c* 0.6, $CHCl_3$); ^1H NMR (300 MHz, $CDCl_3$): δ 9.47 (s, 1H, CHO), 8.28-7.26 (m, 21 H, H-Ar, H-formyl), 5.30 (s, 0.4 H), 5.06-4.40 (br. m, 8 H, CH_2Ar), 4.31- 3.20 (br. m, 5H, H-2, H-3, H-4, H-1a, H-1b); ^{13}C NMR (150 MHz, 90 °C, toluene-d8): δ 190.0 (*C*H=O), 161.2 (NCH=O), 137.4–125.2 (24 x C-Ar), 82.1 (C-4), 81.2 (C-3), 76.2 (O*C*H_2Ph), 75.3 (C-2), 73.1, 73.1, 72.2 (3 x O*C*H_2Ph), 46.1 (C-1). ESI-MS: *m/z* calcd for $C_{34}H_{35}NO_6$ $[M+Na]^+$: 576.2357; found 576.2365.

Dibenzyl [2,3,4-tri-*O*-benzyl-1-(*N*-benzyloxy-*N*-formylamino)-1-deoxy-D-ribo-5-(*E*)-hex-enitol)] 6-phosphonate (23). The bisphosphonate reagent **22** (450 mg, 0.84 mmol) was dissolved in THF (2 mL) and cooled to –78 °C. Then BuLi (0.35 mL, 0.8 mmol) was added dropwise, and the reaction mixture was stirred at –78 °C for 30 min. Then aldehyde **21** (235 mg, 0.042 mmol) was added in one portion and the solution was stirred for 90 min before warming up to rt and continued stirring for 12 h. The

reaction was quenched by adding aq NH_4Cl and the suspension was extracted with EtOAc. The organic layer was washed with brine, dried and concentrated. Purification of the crude by column chromatography (1:2 EtOAc-hexane) gave compound **23** as syrup**.** Yield: 225 mg, (76 %); $[\alpha]_D^{20}$ +3.3 (*c* 0.7, $CHCl_3$). ^{1}H NMR (toluene-d_8, 70°): δ 8.04 (s, 1H, HC=O), 7.28–6.91 (m, 31 H, 30 Ar-H, H-5), 6.08 (m, 1 H, H-6), 4.95–4.89 (m, 4 H, 2 x OCH_2Ph), 4.60–4.50 (m, 4H, 2 x OCH_2Ph), 4.44 (AB, 2 H, OCH_2Ph), 4.29 (AB, 2 H, OCH_2Ph), 4.13 (bt, 1 H, H-4), 3.98 (bt, 1 H, H-2), 3.67 (m, 3 H, H-3, H-1a, H-1b), ^{13}C NMR (toluene-d_8, 70°): δ 149.4 (C-formyl), 137.1 (6 x Ar-C), 129.0–124.3 (24 x Ar-C), 121.5 (C-5), 120.3 (C-6), 81.6 (C-3), 79.6 (C-4), 77.2 (C-2), 76.7 (O*C*H_2Ph), 73.6, 72.6, 71.9 (3 x O*C*H_2Ph), 66.9 (O*C*H_2Ph), 47.3 (C-1). ESI-MS**:** *m/z* calcd for $C_{56}H_{47}N_2O_4P$ $[M+H]^+$: 812.3609; found: 812.3623.

Sodium 1-*N*-formylamino-1-deoxy-D-*ribo*-hexitol 6-phosphonate (24). Compound **23** (35 mg, 0.04 mmol) was dissolved in 1 : 1: 2 THF-MeOH-H_2O (1 mL) and $Pd(OH)_2$ catalyst was added (8 mg). The reaction was stirred under hydrogen atmosphere for 72 h at rt. TLC (3:1 EtOAc-MeOH and 10:10:3 CHCl-MeOH-water) showed several spots. Every 24 h a new portion of catalyst (8 mg) was added. After 72 h most of the product had been formed according to LC-MS. The suspension was filtered, and the filtrate was concentrated. The residue was treated with 8.3 mg $NaHCO_3$ and filtered. The filtrate was concentrated and lyophilised. Purification on a HILIC column (2 x) gave 2.1 mg of **24** (16%) as syrup. $[\alpha]_D^{20}$ -34.7 (*c* 0.2, water); ^{1}H NMR (600 MHz, D_2O, ratio of rotamers 5.5:1): δ 8.42 (s, H-formyl minor rotamer), 7.97 (s, H-formyl major rotamer), 4.15 (ddd, 1 H, *J* = 2.5, *J* = 5.9 Hz, H-2), 3.79 (dd, 1H, *J* = 2.5, *J* = 14.9 Hz, H-1a), 3.76 (ddd, 1 H, *J* = 2.6, *J* = 6.3, *J* = 9.1 Hz, H-4), 3.72 (dd, 1 H, *J* = 9.2, *J* = 15.0 Hz, H-1b),3.69 (dd, 1 H, H-3), 1.96 (dddd, 1 H, *J* = 2.8, *J* = 4.6, *J* = 11.9 Hz, H-5a), 1.83 (ddd, 1 H, *J* = 4.5, *J* = 11.9 Hz, H-6a), 1.69 (dddd, 1 H, *J* = 4.6, *J* = 9.1 Hz, H-5b), 1.61 (ddd, 1 H, *J* = 4.6, *J* = 11.5 Hz, H-6b); ^{13}C NMR (150 MHz, D_2O): δ 160.9 (CHO), 76.0 (C-3), 72.8 (d, C-4), 68.2 (C-2), 54.0 (C-1), 27.2 (C-5), 25.3 (C-6). ^{31}P NMR: δ 25.47.ESI-MS: *m/z* calcd for $C_7H_{16}NO_8P$ $[M]^-$ 272.0541; found: 272.0552.

Sodium 1-*N*-formylamino)-1-deoxy-D-*ribo*-5-(*E*)-hexenitol 6-phosphonate (25). Compound **23** (20 mg, 0.025 mmol) was dissolved in dry DCM (0.5 mL) and a 1 M sol. of $SnCl_4$ in DCM (0.17 mmol) was added dropwise. The solution was stirred at rt overnight. After this time another aliquot (0.1 mmol) of the reagent was added and stirring was continued for another 3 h. Then the mixture was poured into a solution of $NaHCO_3$ (61 mg, 0.73 mmol) in water (2 mL) and stirred for 10 min. DCM was evaporated and the residue containing insoluble Sn(IV) salts was filtered through cotton and a micro filter (2 x). The filtrate was concentrated and purified by ZIC-HILIC chromatography (column: Sequant ZIC HILIC 10 x 250 mm, Merck, 15:85 MeCN-H_2O → 4:6 MeCN-H_2O, flow rate 3 mL, fraction size 2 mL). Purity of fractions was checked by ^{1}H NMR and product containing fractions were pooled and

lyophilized. To remove residual sodium bicarbonate, the material was passed over DOWEX-50 cation exchange resin (H^+-form) and the pH of the filtrate was adjusted to 7.0 by addition of 0.01 M NaOH followed by lyophilization to give **25** as syrup. Yield: 3.6 mg (50%). $[\alpha]_D^{20}$ +4.1 (*c* 0.4, D_2O); ^{1}H NMR (D_2O 600 MHz, ratio of rotamers 7.5:1): δ 8.49 (s, CH=O, minor rotamer), 7.81 (s, 1 H, CH=O, major rotamer), 6.25 (dt, 1 H, *J* = 6.5, *J* = 17.4 Hz, H-5), 6.08 (dt, 1 H, *J* = 16.1 Hz H-6), 4.36 (bt, 1 H, *J* = 6.1 Hz, H-4), 4.06 (ddd, 1 H, *J* = 2.6 Hz, *J* = 6.7 Hz, *J* = 8.3 Hz, H-2), 3.75-3.70 (m, 2 H, H-3, H-1a), 3.64 (ddd, 1 H, *J* = 13.6, *J* = 7.3 Hz, H-1b). ^{13}C NMR (D_2O, 125 MHz): δ 156.07 (C=O), 137.09 (C-5), 131.68 (d, *J* = 167.0 Hz, C-6), 75.09 (C-3), 72.99 (d, *J* = 17.0 Hz, C-4), 67.46 (C-2), 54.34 (C-1). ^{31}P NMR (242 MHz, D_2O): δ 9.62. ESI-MS: *m/z* calcd for $C_7H_{16}NO_8P$ $[M-H]^-$ 272.0541; found: 272.0552.

Dibenzyl [2,3,4-tri-*O*-benzyl-1-(*N*-benzyloxy-*N*-formylamino)-1-deoxy-5,6-dihydroxy-D-ribo-hexitol)]-6-phosphonate (26). Hexenitol **23** (50 mg, 0.06 mmol) was dissolved in 2:1 acetone-water (0.75 mL) and NMO (10 mg, 0.1 mmol) was added. Then potassium osmate (2.25 mg, 6.2 µmol) was added and the reaction was stirred overnight at rt. Since TLC (1:2 EtOAc-hexane) showed only minor conversion, 0.1 equivalents of potassium osmate and 1.6 eq. of NMO were added. This was repeated 3 times until an acceptable conversion was obtained (5 days). The reaction was quenched by adding satd aq Na_2SO_3 followed by extraction with EtOAc. The organic phase was washed with aq 4 % HCl, satd aq $NaHCO_3$, and brine. The organic layer was dried ($MgSO_4$), concentrated and the product was isolated by column chromatography (1:2 EtOAc-hexane) to give **26** as syrup. Yield: 41 mg (79 %). $[\alpha]_D^{20}$ +11.9 (*c* 0.56, $CHCl_3$); ^{1}H NMR (600 MHz, toluene-d_8 80°C): δ 8.09 (s, 1H, H-formyl), 7.30–6.96 (m, 30 H, Ar-H), 5.02 (d, 2 H, OC*H₂*Ph), 4.87 (d, 2 H, OC*H₂*Ph) 4.70–4.62 (m, 6 H, 3 x OC*H₂*Ph), 4.54 (s, 2H, OC*H₂*Ph), 4.44 (t, 1 H, *J* = 6.9 Hz, H-5), 4.41 (d, 1 H, *J* = 9.7 Hz, H-6), 4.19 (dd, 1 H, *J* = 4.2 Hz, *J* = 2.5 Hz, H-3), 4.08 (m, 2 H, H-3, H-2), 3.80 (bs, 2 H, H-1a, H-1b); ^{13}C NMR (150 MHz): δ 138.5 –124.5 (30 x Ar-C), 80.4 (C-4), 79.4 (C-2), 76.8 (C-3), 76.4 (O*C*H₂Ph), 74.5 (O*C*H₂Ph), 73.5, 72.7 (2 x O*C*H₂Ph), 70.6 (C-5), 68.8 (O*C*H₂Ph), 68.5 (C-6), 67.5 (O*C*H₂Ph), 48.9 (C-1). ESI HR-MS: *m/z* cald for $C_7H_{16}NO_8P$ $[M+H]^+$: 846.3402; found: 846.3401.

Sodium 1-(*N*-benzyloxy-*N*-formylamino)-1-deoxy-5,6-dihydroxy-D-*ribo*-hexitol)] 6-phosphonate (27). Compound **26** (24 mg, 0.03 mmol) was dissolved in 1:2 THF-water and $Pd(OH)_2$ catalyst (4 mg) was added. The suspension was stirred overnight and checked by TLC ($CHCl_3$-MeOH-water 10:10:3). After completeness, solid sodium bicarbonate (4 mg, 0.09 mmol) was added, and all volatiles were removed. Then the crude was purified by repeated ZIC-HILIC chromatography (column: Sequant ZIC HILIC 10 x 250 mm, Merck, Gradient MeCN-H_2O 85 to 40 %, flow rate 3 mL, fraction size 2 mL). Purity of fractions was checked by ^{1}H NMR and product containing fractions were pooled and lyophilized to give 27 (2.4 mg, 24%) as colorless solid. $[\alpha]_D^{20}$ –20.5 (*c* 0.24, water). ^{1}H NMR (D_2O, 600 MHz, pD =

7.4): δ 8.29 (s, 0.2 H, NC(O)H, minor rotamer), 7.84 (s, 0.8 H, NC(O)H, major rotamer), 4.22 (m, 1H, *J* = 5 Hz, H-2), 4.01 (dt, 1H, H-5), 3.88 (m, 2H, H-3, H-4), 3.83 (dd, 1H, $J_{6,5}$ = 2.5 Hz, $J_{6,P}$ = 10 Hz, H-6), 3.72 (dd, 1H, $J_{1a,2}$ = 2 Hz, $J_{1b,1a}$ = 15 Hz, H-1a), 3.65 (dd, 1H, $J_{1b,2}$ = 9 Hz, H-1b). ^{13}C NMR (150 MHz): δ 159.7 (CH=O), 74.65 (C-3), 72.85 (C-5), 72.1 (C-4), 69.3 (C-6), 68.4 (C-2) and 54.4 (C-1). ESI HR-MS: *m/z* calcd for $C_7H_{16}NO_{10}P$ [M-H]$^-$: 304.0439; found 304.0440.

Dibenzyl [2,3,4-tri-*O*-benzyl-1-(*N*-benzyloxyamino)-1,6-dideoxy-D-*allo/*L-*talo*-hexitol)] 6-phosphonate (29) and dibenzyl [2,3,4-tri-*O*-benzyl-1-(*N*-benzyloxy-*N*-formylamino)-1,6-dideoxy-D-*allo/*L-*talo*-hexitol)]-6-phosphonate (30) Dibenzyl methanephosphonate **28** (134 mg, 0.48 mmol) was dissolved in THF (1.5 mL) and cooled to –78 °C. Then a 2 M solution of BuLi in hexane (190 µL) was added dropwise and the reaction was stirred at the same temperature for 30 min. Subsequently aldehyde **21** (180 mg, 0.32 mmol) was added dropwise and the solution was stirred at –78 °C. The reaction was quenched after 90 min by adding aq NH_4Cl. The suspension was extracted with EtOAc, washed with brine. dried ($MgSO_4$), concentrated. The residue was purified by column chromatography (1:2 EtOAc-hexane) to give first 50 mg (28 %) of recovered aldehyde **21** followed by the deformylated product **30** (55 mg, 22 %) and **29** (25 mg, 10%) as syrup; $[\alpha]_D^{20}$ +8.6 (*c* 0.45, $CHCl_3$); ^{1}H NMR (toluene-d_8 , 90 °C): δ 8.06 (s, 1 H, H-formyl), 7.30–6.94 (m, 30 H, Ar-H), 4.95–4.44 (13 H, 6 x OC*H*$_2$Ph, H-2), 4.20 (bt, 1 H, H-5), 3.93 (bt, 1 H, H-4), 3.83 (bt, 1 H, H-3), 3.77 (m, 2 H, H-6a, H-6b), 2.21 (ddd, 1 H, *J* = 15.0 and 2.5 Hz, H-1a), 2.12 (ddd, 1 H, H-1b). ^{13} C NMR (150 MHz, toluene-d_8 90 °C): δ 161.0 (C=O), 137.4–124.6 (36 Ar-C), 82.5 (d, C-P coupling, *J* = 14 Hz, C-4), 79.8 (C-3), 76.8 (C-2), 76.7 (O*C*H$_2$Ph), 74.2, 73.5, 72.7 (3 x O*C*H$_2$Ph), 67.5 (d, C-P coupling, *J* = 6 Hz, C-5), 67.0, 67.0 (2 x O*C*H$_2$Ph), 48.3 (C-1), 29.8 (d, $J_{C\text{-}P}$ = 138 Hz, C-6).[31] P NMR (242 MHz, toluene-d_8 90 °C) : δ 25.2 (s). ESI HR-MS: *m/z* calcd for $C_{49}H_{52}NO_9P$ [M+H]$^+$: 830.3452; found 830.3448.

^{1}H NMR for **30** (toluene-d_8 90 °C, major isomer): δ 7.27–6.95 (m, 30 H, Ar-H), 4.92–4.80 (m, 4 H, 2 x OC*H*$_2$Ph), 4.68–4.48 (m, 9 H, 4 x OC*H*$_2$Ph, H-5), 4.13 (ddd, 1 H, H-2), 3.94 (dd, 1 H, *J* = 4.6 Hz, H-3), 3.82 (dd, 1 H, *J* = 4.9 Hz, H-4), 3.36 (dd, 1 H, *J* = 4 Hz, *J* = 14 Hz, H-1a), 3.21 (dd, 1 H, *J* = 7 Hz, H-1b), 2.26 (ddd, 1 H, *J* = 3, *J* = 15 Hz, H-6a), 2.14 (ddd, 1 H, *J* = 7 Hz, H-6b). ^{13}C NMR (toluene-d_8 90 °C): δ 137.4–124.4 (36 Ar-C), 82.4 (d, $J_{C\text{-}P}$ = 15 Hz, C-4), 80.2 (C-3), 77.1 (C-2), 75.7, 74.0, 73.5, 72.4 (4 x O*C*H$_2$Ph), 67.6 (d, $J_{C\text{-}P}$ = 5 Hz, C-5), 67.0, 66.9 (2 x OC*H*$_2$Ph), 52.8 (C-1), 29.9 (d, $J_{C\text{-}P}$ = 140 Hz, C-6). ^{31} P NMR (242 MHz, toluene-d_8 90 °C): δ 32.5. ESI HR-MS: *m/z* calcd for $C_{48}H_{52}NO_8P$ [M+H]$^+$: 802.3503; found 802.3525.

Formylation reaction of 30. Carbonyldiimidazole (62 mg, 0.38 mmol) was dissolved in THF (1 mL) and cooled to 0 °C. Then formic acid (15 µL, 0.38 mmol) was added and the mixture was stirred at 0° C for 30 min under Ar. Meanwhile a solution of **30** (60 mg, 0.075 mmol) in THF (0.5 mL) was

prepared. The solution of CDI and formic acid was then added, and the mixture was stirred at rt for 36 h. Then all volatiles were evaporated, the residue was coevaporated with toluene and purified by column chromatography (1:2 EtOAc-hexane) to give unreacted **30** (15 mg, 24%) followed by **29** (45 mg, 73%).

Sodium [1-(*N*-hydroxy-*N*-formylamino)-1,6-dideoxy-D-*allo*/L-*talo*-hexitol)]-6-phosphonate (31) Compound **29** (20 mg, 0.024 mmol) was dissolved in 1:2 THF-water and $Pd(OH)_2$ catalyst (4 mg) was added. The suspension was stirred overnight and checked by TLC (10:10:3 $CHCl_3$-MeOH-H_2O). The catalyst was filtered off and the filtrate was washed with 1:1 MeOH-H_2O and purified twice by HILIC separation (column: Sequant ZIC HILIC 10 x 250 mm, Merck, gradient 15:85 MeCN-H_2O → 4:6 MeCN-H_2O, flow rate 3 mL, fraction size 2 mL). Purity of fractions was checked by ^{1}H NMR and product containing fractions were pooled and lyophilized to give **31** (0.65 mg, 10%) as colorless solid; $[\alpha]_D^{20}$ +17.4 (*c* 0.2, water); ^{1}H NMR (D_2O, 600 MHz, ratio of rotamers 4.1:1): δ 8.31 (s, CHO, minor form), 7.86 (s, 1 H, CHO major isomer), 4.27 (ddd, 1 H, *J* = 8.7, *J* = 4.7 Hz, H-2), 4.19 (dt, 1 H, *J* = 3.6, *J* = 3.6, *J* = 9.8 Hz, H-5), 3.80 (dd, 1H, *J* = 8.3, *J* = 4.0 Hz, H-3), 3.76 (dd, 1H, *J* = 8.2, *J* = 4.4 Hz, H-4), 3.72-3.70 (m, 2H, H-1a, H-1b),1.11 (ddd, 1 H, *J* = 3.5, *J* = 14.6 Hz, H-6a), 1.65 (ddd, 1 H, *J* = 9.3, *J* = 14.9 Hz, H-6b); ^{13}C NMR (D_2O, 150 MHz): δ 75.13 (d, *J* = 9.3 Hz, C-4), 73.62 (C-3), 69.82 (C-5), 68.53 (C-2), 54.06 (C-1), 30.79 (d, *J* = 104.1 Hz, C-6); ^{31}P NMR (242 MHz, D_2O): δ 29.4. ESI HR-MS: *m/z* calculated for $C_7H_{16}NO_9P$ $[M-H]^-$: 288.0490; found 288.0495.

Phenyl 2,3,4,6-tetra-*O*-acetyl-1-thio-D-altropyranoside (87). Acetic anhydride (50 mL) and a catalytic amount of DMAP were added to a solution of D-altrose **85** (2 g, 11 mmol) in dry pyridine (60 mL) at ice-bath temperature. The mixture was then stirred at rt for 30 min, whereupon TLC (3:2 hexane-EtOAc) showed complete conversion. EtOAc (150 mL) was added and the mixture was washed with 1 M aq HCl (200 mL). The aqueous phase was extracted with EtOAc (3 × 50 mL). The combined organic layers were sequentially washed with water and satd aq $NaHCO_3$, then dried ($MgSO_4$), filtered and concentrated. Diethyl ether was added to the residue and the solution was filtered through a short Florisil pad. The eluate was concentrated to afford penta-*O*-acetyl-D-altrose **86** (ratio α/β/furanose: 1:0.07:0.08) as a pale yellow oil (4.3 g, ~quant.). Thiophenol (16.5 mmol, 1.7 mL) was then added to a solution of **86** (4.3 g, 11 mmol) in anhydrous dichloromethane (60 mL) under argon. Then $BF_3{\cdot}Et_2O$ (55 mmol, 6.8 mL) was added dropwise at 0 °C. The reaction mixture was allowed to warm to rt and was stirred overnight. Additional thiophenol (8.8 mmol, 0.9 mL) and $BF_3{\cdot}Et_2O$ (0.04 mmol, 5.1 mL) were added and stirring was continued for 4 h. The reaction mixture was diluted with CH_2Cl_2, cooled to 0 °C, washed with satd aq $NaHCO_3$ followed by re-extraction of the aqueous phase with CH_2Cl_2 (3 x). The combined organic phases were then washed with water, dried

($MgSO_4$) and concentrated. The crude was purified by column chromatography on silica gel (7:3 → 3:2 hexane-EtOAc) to afford **87** as a colorless oil (4.0 g, 82%, ratio of α-pyranose/β-pyranose/furanose = 1 : 0.1 : 0.15), along with recovered starting material **86** (244 mg, 6%). ^{1}H NMR ($CDCl_3$, 300 MHz, α-anomer): *δ* 7.57-7.45 (m, 2 H, Ph), 7.35-7.23 (m, 3 H, Ph), 5.41 (br d, 1 H, H-1), 5.33 (td, 1 H, $J_{1,3}$ = 0.9, $J_{2,3}$ = $J_{3,4}$ = 3.4 Hz, H-3), 5.24-5.17 (m, 2 H, H-2, H-4), 4.81 (ddd, 1 H, $J_{4,5}$ = 10.2, $J_{5,6a}$ = 5.7, $J_{5,6b}$ = 2.2 Hz, H-5), 4.36 (dd, part A of ABX system, 1 H, $J_{6a,6b}$ = 12.2 Hz, H-6a), 4.20 (dd, part B of ABX system, 1 H, H-6b), 2.23, 2.14, 2.08 and 2.05 (4 x s, each 3 H, 4 x Ac). ^{13}C NMR ($CDCl_3$, 75 MHz, α-anomer): *δ* 170.7, 169.6, 169.4, 169.4 (C=O), 135.2 (Cq, Ph), 131.9, 129.2, 127.9, (*C*H, Ph), 86.1 (C-1), 71.4 (C-2), 66.9 (C-3), 65.6, 65.3 (C-4, C-5), 62.8 (C-6), 20.9, 20.8, 20.7 and 20.6 (CH_3CO).

Phenyl 1-thio-D-altropyranoside (88). A solution of **87** (1.55 g, 3.52 mmol) in 8:1:1 MeOH-H_2O-Et_3N was stirred at rt overnight. The solution was concentrated and the residue was dissolved in water and lyophilized to give **88** as colorless amorphous solid. Yield: 873 mg (91%); $[\alpha]_D^{23}$ +62 (*c* 1.0, MeOH); ^{1}H NMR (MeOD, 300 MHz): *δ* 7.58-7.50 (m, 2 H, Ph), 7.33-7.20 (m, 3 H, Ph), 5.30 (brd, 1 H, $J_{1,2}$ = 1.6 Hz, H-1), 4.33 (ddd, 1 H, $J_{5,6a}$ = 2.9, $J_{5,6b}$ = 5.1 Hz, H-5), 4.08 (dd, 1 H, $J_{1,2}$ = 1.6, $J_{2,3}$ = 3.6 Hz, H-2), 3.98-3.90 (m, 2 H, H-3, H-4), 3.88 (dd, 1 H, $J_{5,6a}$ = 2.9, $J_{6a,6b}$ = 11.9 Hz, H-6a), 3.81 (dd, 1 H, $J_{5,6b}$ = 5.2 Hz, H-6b). ^{13}C NMR (MeOD, 75 MHz): *δ* 139.0 (Cq, Ph), 132.2, 129.8, 127.8, (*C*H, Ph), 89.7 (C-1), 74.4 (C-2), 71.7, 71.4 (C-3, C-5), 66.0 (C-4), 63.0 (C-6). HRMS (ESI$^+$): *m/z* calcd for $C_{12}H_{16}O_5S$ $[M + NH_4]^+$ 290.1057; found: 290.1056.

Phenyl 6-*O*-triisopropylsilyl-1-thio-α-D-altropyranoside (89). DMAP (cat. amount) was added to a solution of **88** (823 mg, 3.03 mmol, ratio: α-pyranoside/furanoside = 1 : 0.11) in dry pyridine (30 mL) under argon and cooled to 0 °C. TIPSCl (2 equiv., 6.05 mmol, 1.16 g, 1.3 mL) was then added dropwise, the mixture was warmed to rt and stirred for 3 h. Second portions of TIPSCl (1 equiv. 0.7 mL) and DMAP (cat. amount) were added and the mixture was stirred overnight, followed by a third addition of TIPSCl (1 equiv., 0.7 mL) and DMAP. The mixture was stirred for 7 h, when TLC (1:9 EtOAc-MeOH,) showed virtually complete conversion. EtOAc was added and the organic phase was washed with water. The aqueous phase was extracted with EtOAc (3 ×). The combined organic phases were washed with water, dried ($MgSO_4$) and concentrated. The residue was subjected to chromatography on silica-gel (2:3 EtOAc-hexane) to give first the furanoside (60 mg) followed by a 10 : 0.7 mixture of α-pyranoside/furanoside (963 mg) and pure **89** (199 mg) as a colorless oil; $[\alpha]_D^{22}$+154 (*c* 1.2, $CHCl_3$). ^{1}H NMR ($CDCl_3$, 300 MHz): *δ* 7.52-7.44 (m, 2 H, Ph), 7.33-7.20 (m, 3 H, Ph), 5.31 (d, 1 H, $J_{1,2}$ = 2.4 Hz, H-1), 4.37 (ddd, 1 H, H-5), 4.18 (ddd, 1 H, H-2), 4.11-3.98 (m, 3 H, $J_{5,6a}$ = 4.8, $J_{6a,6b}$ = 10.0 Hz, H-3, H-4, H-6a), 3.93 (dd, part B of ABX system, 1 H, $J_{5,6b}$ = 7.7 Hz, H-6b), 3.71 (d, 1 H, *J* = 1.6 Hz, OH), 2.85 (d, 1 H, *J* = 1.6 Hz, OH,), 2.18 (d, 1 H, *J* = 6.3 Hz, OH-2), 1.14-1.05 (m, 21 H, 3 × *i*-Pr, TIPS). ^{13}C NMR ($CDCl_3$,

75 MHz): δ 136.3 (Cq, Ph), 131.2, 129.1, 127.3, (*C*H, Ph), 87.9 (C-1), 71.9 (C-3), 70.1 (C-2), 69.5 (C-4), 68.4 (C-5), 66.3 (C-6), 18.0 (Me, *i*-Pr, TIPS), 11.9 (C*H*, i-Pr, TIPS). HRMS (ESI$^+$): *m/z* calcd for $C_{21}H_{36}O_5SSi$ $[M + Na]^+$ 451.1945; found: 451.1958.

Phenyl 2,3,4-tri-*O*-benzyl-6-*O*-triisopropylsilyl-1-thio-α-D-altropyranoside (90). NaH (60%, 6 equiv., 13.5 mmol, 540 mg) was added to a solution of **89** (963 mg, 2.25 mmol, ratio of α pyranoside/furanoside = 10 : 0.7) in dry DMF (60 mL) under argon at 0 °C. After a few minutes, benzyl bromide (6 equiv., 13.5 mmol, 1.6 mL) was added dropwise. The mixture was stirred at rt for 2 h, whereupon TLC (1:2 EtOAc-hexane) showed complete conversion. The mixture was cooled to 0 °C and water was added. The phases were separated, and the aqueous phase was extracted with diethyl ether (3 ×). The combined organic phases were washed with water, dried ($MgSO_4$) and concentrated. The residue was purified by chromatography on silica-gel (1:23 EtOAc-hexane) to give pure **90** as a colorless oil (131 mg) followed by **90** containing residual furanoside (1.369 g, ratio α-pyranoside/furanoside = 10:0.8 mixture); total yield = 1.5 g, (96%); $[\alpha]_D^{20}$ +70 (*c* 1.0, $CHCl_3$). ^1H NMR ($CDCl_3$, 600 MHz): δ 7.53-7.50 (m, 2 H, Ph), 7.39-7.17 (m, 18 H, Ph), 5.47 (br.s, 1 H, H-1), 4.71 (d, part A of AB system, 1 H, *J* = 12.4 Hz, OCH_2), 4.62 (d, part B of AB system, 1 H, OCH_2), 4.57 (d, part A of AB system, 1 H, *J* = 12.4 Hz, OCH_2), 4.54-4.47 (m, 3 H, OCH_2, H-5), 4.39 (d, part B of AB system, 1 H, OCH_2), 4.03-3.95 (m, 3 H, $J_{5,6a}$ = 4.0, $J_{6a,6b}$ = 11.0 Hz, H-6a, H-6b, H-4), 3.96 (dd, 1 H, $J_{1,2}$ = 1.5, $J_{2,3}$ = 4.1 Hz, H-2), 3.83 (t, 1 H, $J_{2,3} = J_{3,4}$ = 3.4 Hz, H-3), 1.14-1.04 (m, 21 H, 3 × *i*-Pr, TIPS). ^{13}C NMR ($CDCl_3$, 150 MHz) δ: 138,7, 138.4, 138.3, 138.1 (4 × Cq, Ph), 130.5, 128.8, 128.6, 128.5, 128.4, 128.1, 128.0, 127.9, 127.8, 127.7, 127.6, 126.6 (*C*H, Ph), 85.6 (C-1), 78.1 (C-2), 73.7 (C-3), 72.9 (C-4), 72.6, 72.3, 72.0, 72.0, 70.2 (4 × CH_2Ph, C-5), 63.2 (C-6), 18.2, 18.1 (Me, *i*-Pr, TIPS) 12.2 (C*H*, *i*-Pr, TIPS). HRMS (ESI$^+$): *m/z* calcd for $C_{42}H_{54}O_5SSi$ $[M + NH_4]^+$ 716.3799; found: 716.3803.

Phenyl 2,3,4-tri-*O*-benzyl-1-thio-α-D-altropyranoside (91). A 1 M solution of TBAF in THF (0.2 mL, 0.2 mmol) was added to a solution of **90** (34 mg, 0.05 mmol) in THF (1.3 mL) under argon and the solution was stirred overnight at rt. The solution was diluted with diethyl ether and washed with satd aq NH_4Cl. The aqueous layer was extracted with Et_2O (3 ×), the combined organic layers were dried ($MgSO_4$) and concentrated. The residue was purified by chromatography on silica gel (1:5 →1:2.5, hexane-EtOAc) to give **91** (17 mg, 64%) as a colorless oil. This protocol was also applied to a 10:0.8 mixture of α-pyranoside/furanoside (1.369 g, 1.95 mmol) which afforded 628 mg (60%) of the deprotected phenyl 1-thioaltroside. $[\alpha]_D^{25}$ +103 (*c* 0.6, $CHCl_3$). ^1H NMR (toluene-d_8, 600 MHz): δ 7.56-7.53 (m, 2 H, Ph), 7.39-7.36 (m, 2 H, Ph), 7.23-7-20 (m, 2 H, Ph), 7.17-6.97 (m, 14 H, Ph), 5.61 (br.s, 1 H, H-1), 4.79 (dt, 1 H, $J_{5,6a} = J_{5,6b}$ = 3.3, $J_{4,5}$ = 9.8 Hz, H-5), 4.65 (d, part A of AB system, 1 H, *J* = 12.4 Hz, OCH_2), 4.56 (d, part B of AB system, 1 H, OCH_2), 4.34 (s, 2 H, OCH_2), 4.26 (d, part A of AB system, 1 H,

J = 12.1 Hz, OCH_2), 4.09-4.05 (m, 2 H, $J_{3,4}$ = 2.9 Hz, H-4, OCH_2), 4.00 (br.d, 1 H, $J_{2,3}$ = 3.7 Hz, H-2), 3.88-3.83 (m, 2 H, H-3, H-6), 3.81 (dd, part B of ABX system, 1 H, $J_{5,6a}$ = 4.0, $J_{6a,6b}$ = 11.7 Hz, H-6b). ^{13}C NMR (toluene-d$_8$, 150 MHz): δ 86.5 (C-1), 78.8 (C-2), 74.0 (C-3), 73.6, 73.1, 72.1, 71.8 (C-4, 3 × CH$_2$Ph), 69.5 (C-5), 62.6 (C-6). HRMS (ESI$^+$): *m/z* calcd for $C_{33}H_{34}O_5S$ [M + NH_4]$^+$ 560.2465; found: 560.2467; calcd for [M + Na]$^+$ 565.2019; found: 565.2024.

Phenyl 2,3,4-tri-*O*-benzyl-6-*O*-(bisphenoxy-phosphoryl)-1-thio-α-D-altropyranoside (92). A solution of **91** (245 mg, 0.45 mmol), triethylamine (0.1 mL, 0.72 mmol) and DMAP (cat. amount) in dry dichloromethane (25 mL) was cooled to 0 °C under argon, followed by addition of diphenyl phosphorochloridate (0.1 mL, 0.48 mmol). The reaction mixture was allowed to warm up to rt and was stirred overnight. The mixture was diluted with dichloromethane (20 mL) and then washed with satd aq $NaHCO_3$. The organic phase was dried ($MgSO_4$) and concentrated. The residue was purified by column chromatography on silica gel (1:4 hexane-EtOAc, containing 1% of NEt_3) to give **92** (338 mg, 97%) as a colorless oil; $[\alpha]_D^{24}$+20.7 (*c* 0.5, $CHCl_3$). ^{1}H NMR ($CDCl_3$, 300 MHz): δ 7.50-7.42 (m, 2 H, Ph), 7.40-7.15 (m, 28 H, Ph), 5.46 (br.s, 1 H, H-1), 4.74 (ddd, 1 H, $J_{5,6}$ = 3.0 and 5.8 Hz, H-5), 4.68 (d, part A of AB system, 1 H, J = 12.3 Hz, OCH_2), 4.61-4.49 (m, 4 H, H-6a, H-6b, OCH_2), 4.38 (d, part B of AB system, 1 H, J = 12.2 Hz, OCH_2), 4.30 (br s, 1 H, OCH_2), 3.98 (dd, 1 H, $J_{2,1}$ = 0.9, $J_{2,3}$ = 3.5 Hz, H-2), 3.90 (dd, 1 H, $J_{3,4}$ = 2.9, $J_{4,5}$ = 10.0 Hz, H-4) and 3.82 (t, 1 H, H-3). ^{13}C NMR ($CDCl_3$, 75 MHz): δ 150.7, 137.9, 137.6 (Cq, Ph), 130.7, 129.8, 129.7, 129.7,129.0, 128.6, 128.5, 128.5, 128.1, 128.0, 127.9, 127.8, 126.9, 125.3, 120.4, 120.3, 120.3, 120.3 (CH, Ph), 85.9 (C-1), 77.4 (C-2), 72.6, 72.4, 72.3, 72.1 (2 x CH$_2$Ph, C-3, C-4), 71.5 (CH$_2$Ph), 68.5 (C-6, $J_{C-6,P}$ = 5.6 Hz), 67.3 (C-5, $J_{C-5,P}$ = 8.2 Hz). ^{31}P NMR ($CDCl_3$, 121.5 MHz) δ: -11.97

2,3,4-Tri-*O*-benzyl-6-*O*-(bisphenoxy-phosphoryl)-D-altro-pyranose (93) *N*-bromosuccinimide (NBS, 5 equiv, 2 mmol, 380 mg) was added to a solution of **92** (331 mg, 0.43 mmol) in 5:1 acetone-water (36 mL) at -11 °C in the dark. After 10 min at -11 to 0 °C, whereupon complete conversion was observed by TLC, the reaction mixture was diluted with dichloromethane (40 mL). The organic phase was washed with satd aq $NaHCO_3$ (10 mL) and satd aq $Na_2S_2O_3$ soln. (10 mL). The combined aqueous phases were extracted with dichloromethane (3 ×) and the combined organic phase was dried ($MgSO_4$) and concentrated. The residue was purified by column chromatography on silica gel (1:2 EtOAc-hexane) to yield **93** as colorless oil (284 mg, 98%).^{1}H NMR ($CDCl_3$, 300 MHz, α/β ratio = 1.8:1): δ 7.39-7.06 (m, Ph), 5.06 (br s, H-1α), 5.04 (d, $J_{1,2(\beta)}$ = 1.8 Hz, H-1 β), 4.71 (d, part A of AB system, J = 11.7 Hz, OCH_2), 4.65-4.23 (m, H-6a α, H-6a β, H-5α, OCH_2-α, OCH_2-β), 4.11 (ddd, H-5 β), 3.95-3.87 (m, $J_{3,4(\alpha)}$ = 2.8, $J_{4,5(\alpha)}$ = 10.7 Hz, H-3 α, H-4 α,), 3.78-3.71 (m, $J_{3,4(\beta)}$ = 2.8, $J_{4,5(\beta)}$ = 10.7 Hz, H-3 β, H-4 β), 3.60 (br d, 1 H, $J_{2,3(\alpha)}$ = 3.6 Hz, H-2 α), 3.48 (dd, 1 H, $J_{2,3(\beta)}$ = 3.5 Hz, H-2 β). ^{13}C NMR ($CDCl_3$, 75 MHz): δ

150.8, 150.7, 138.0, 137.8, 137.7, 137.6, 137.0 (Cq, Ph), 129.8, 129.8, 128.8, 128.8,128.7, 128.6, 128.6, 128.5, 128.4, 128.2, 128.2, 128.1, 128.1, 128.1, 128.0, 125.3, 125.3, 120.5, 120.4, 120.4, 120.3 (*C*H, Ph), 93.1 (C-1 α), 92.0 (C-1 β), 76.9 (C-2 β), 74.9 (C-2 α), 74.3, 74.3, 73.6, 73.3, 72.3, 72.2, 72.2, 72.0, 71.9 (*C*H_2Ph, C-3, C-4, α, β), 71.3 (C-5 β, $J_{C\text{-}5,P}$ = 8.2 Hz), 68.5 (C-6 β, $J_{C\text{-}6,P}$ = 6.2 Hz), 68.4 (C-6 α, $J_{C\text{-}6,P}$ = 5.9 Hz), 66.1 (C-5 α, $J_{C\text{-}5,P}$ = 8.2 Hz). ^{31}P NMR ($CDCl_3$, 121.5 MHz) *δ*: -11.87, -12.02. HRMS (ESI$^+$): *m/z* calcd for $C_{39}H_{39}O_9P$ [*M* + NH_4]$^+$ 700.267; found: 700.2668.

2,3,4-Tri-*O*-benzyl-6-*O*-(bisphenoxy-phosphoryl)-D-altrono-1.5-lactone (94) A suspension of **93** (37 mg, 54 μmol) and activated 4 Å molecular sieves (150 mg) in anhydrous dichloromethane (30 mL) was stirred at room temp for 20 min. Then PCC (148 mg, 0.69 mmol) was added, and the suspension was stirred for 1 h. Within 4 h, 4 additional portions of PCC (4 x 40 mg) were added to drive the reaction to completion. EtOAc (20 mL) was added, followed by filtration over a Florisil pad. The eluate was concentrated and purified by silica gel chromatography (3:1 hexane-EtOAc, containing 0.1% of TEA) to give lactone **94** (24 mg, 65%) as a colorless oil, $[\alpha]_D^{22}$ -4.0 (*c* 0.5, $CHCl_3$). ^{1}H NMR ($CDCl_3$, 300 MHz): *δ* 7.38-7.10 (m, 23 H, Ph), 6.95-6.87 (m, 2 H, Ph), 4.81-4.70 (m, 2 H, H-5, H-a, Bn, *J* = 11.8 Hz. H-5, OCH_2), 4.57-4.48 (m, H-6a, H-6b, OCH_2), 4.35 (2 d, AB system, 2 H, C*H*$_2$Ph, *J* = 11.4 Hz, OCH_2), 4.13 (dd, 1 H, $J_{3,4}$ = 2.4, $J_{4,5}$ = 8.9 Hz, H-4), 4.06 (d, 1 H, $J_{2,3}$ = 4.2 Hz, H-2), 3.87 (dd, 1 H, H-3). ^{13}C NMR ($CDCl_3$, 75 MHz): *δ* 167.2 (*C*O), 150.6, 150.4, 137.4, 137.2 (Cq, Ph), 130.0, 130.0, 130.0, 129.8, 128.3, 128.3,128.2, 128.0, 125.7, 125.7, 125.6, 120.8, 120.4, 120.3, 120.3, 120.3 (*C*H, Ph), 76.4 (C-5, $J_{C\text{-}5,P}$ = 8.4 Hz), 74.9 (C-2), 73.3 (C-3), 73.2 (*C*H_2Ph), 72.9, 72.6 (2 x *C*H_2Ph), 70.6 (C-2), 67.0 (C-6, $J_{C\text{-}5,P}$ = 5.4 Hz). ^{31}P NMR ($CDCl_3$, 121.5 MHz): *δ* -12.18. HRMS (ESI$^+$): calcd for $C_{39}H_{37}O_9P$ [*M* + H]$^+$ 681.2248; found 681.2266; calcd for [*M* + NH_4]$^+$: 698.2513; found: 698.2530.

D-Altrono-1.4-lactone 6-*O*-dihydrogenphosphate (95). A catalytic amount of 10% Pd/C was added to a solution of aldonolactone **94** (93 mg, 0.14 mmol) in anhydrous THF (6 mL). The suspension was placed under H_2 atmosphere and stirred overnight at rt, whereupon TLC showed complete conversion (EtOAc). The catalyst was filtered off, the eluate was concentrated under vacuum, the residue was dissolved in water and lyophilized. The resulting crude compound (52 mg) was dissolved in THF (4 mL), and PtO_2 (cat. amount) was added. The suspension was stirred under hydrogen atmosphere overnight, whereupon TLC (10:9:1 EtOAc-MeOH-H_2O) showed complete conversion. The catalyst was filtered off and the eluate was concentrated in vacuum. The residue was dissolved in water and extracted with diethyl ether. The aq phase was lyophilized to give a white hygroscopic solid which was subjected to gel filtration using a PD-10 Sephadex G 25 column (water as eluent). The eluate was lyophilized to give **95** as amorphous solid (32.6 mg, 90%) containing the free acid in a 3:1 ratio; data for **95**: ^{1}H NMR (D_2O, 600 MHz): *δ* 4.58 (d, 1 H, $J_{2,3}$ = 8.6 Hz, H-2), 4.41 (t, 1 H, H-3), 4.36

(dd, 1 H, , $J_{3,4}$ = 8.1, $J_{2,3}$ = 3.9 Hz, H-4), 4.20 (ddd, 1 H, H-5), 4.08-4.01 (m, H-6a), 3.99-3.94 (ddd, H-6 b).^{13}C NMR (D_2O, 150 MHz) δ: 176.7 (C-1), 80.7 (C-4), 77.8 (C-2), 73.6 (C-3), 69.9 (d, C-5, $J_{C\text{-}5,P}$ = 7.7 Hz), 66.2 (d, C-6, $J_{C\text{-}6,P}$ = 4.9 Hz).^{31}P NMR ($CDCl_3$, 242.9 MHz) δ: 0.27.LCMS: $[M + H]^+$ 259; $[M + Na]^+$ 281; $[M - H]^+$ 257.

D-Altronohydroxamic acid 6-(dihydrogen phosphate) (96). A 50% aqueous hydroxylamine solution (0.4 mmol, 24 µL) was added to a solution of D-altrono-γ-lactone 6-(dihydrogen phosphate) **95** (10 mg, 39 µmol) in water (1.5 mL). The reaction mixture was stirred at rt for 4 h. The solution was concentrated under vacuum and then lyophilized. The residue was subjected to gel filtration chromatography (Bio-gel P-2) to afford **96** (10 mg, 88%). During prolonged NMR measurements, the compound slowly degraded (inter alia 6→5 phosphate migration). ^{1}H NMR (D_2O, 600 MHz) δ: 4.50 (d, 1 H, $J_{2,3}$ = 1.7 Hz, H-2), 4.06-3.91 (m, 4 H, H-3, H-5, H-6a, H-6b), 3.79 (dd, 1 H, $J_{4,5}$ = 4.7, $J_{4,3}$ = 8.8 Hz, H-4).^{13}C NMR (D_2O, 150 MHz) δ: 172.18 (C-1), 72.90 ($J_{C,P}$ = 6.3 Hz, C-5), 72.82 (C-3), 71.54 (C-2), 71.29 (C-4), 65.54 ($J_{C,P}$ = 4.2 Hz, C-6); ^{31}P NMR (D_2O, 242.9 MHz) δ: 2.67. LCMS: calcd for $C_6H_{14}NO_{10}P$ $[M + H]^+$: 292.0; found: 292; $[M - H]^-$: 290.

^{1}H, ^{13}C and ^{31}P NMR spectra of deprotected hydroxamate derivatives

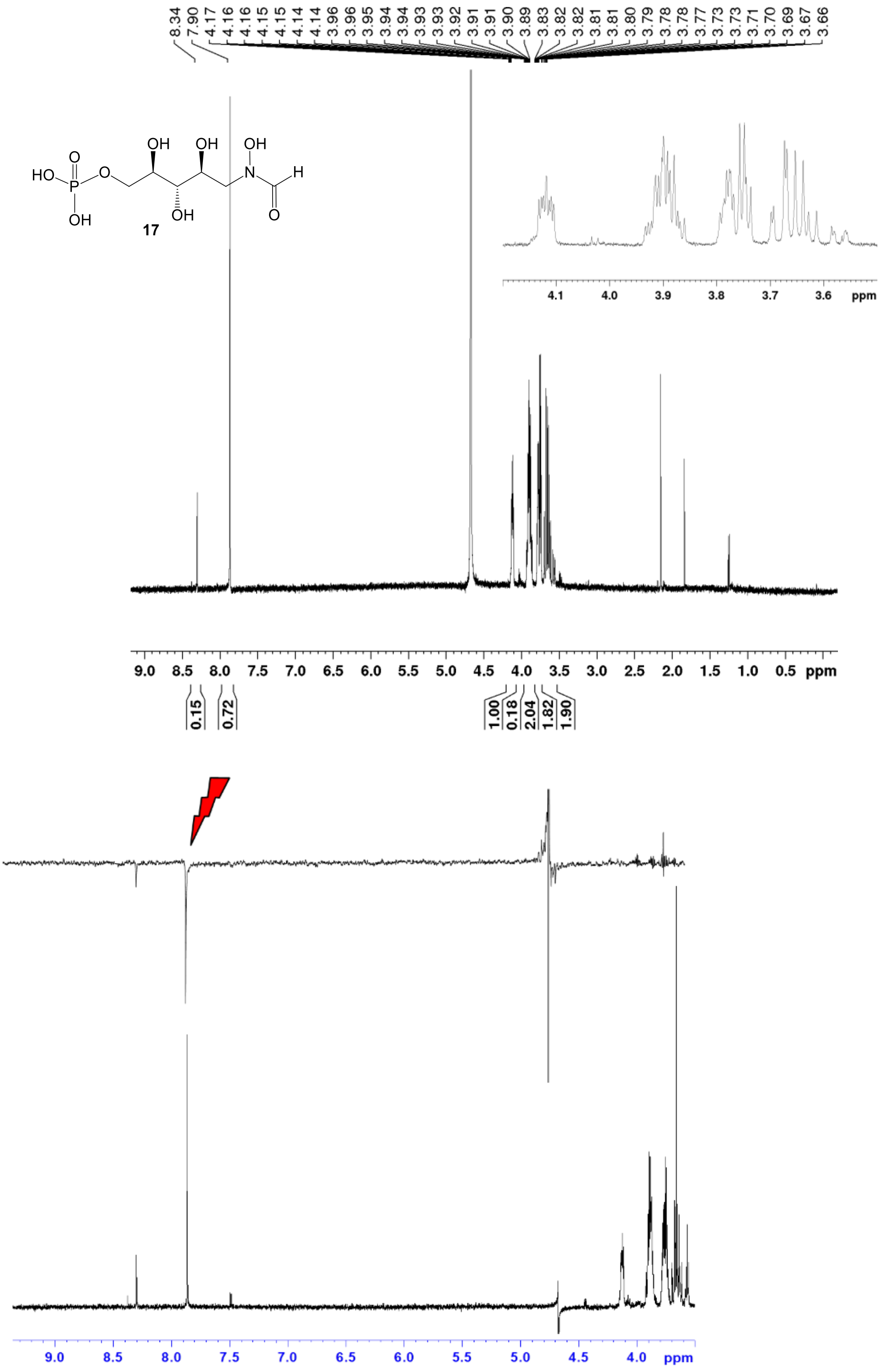

Figure S2. 1D NOe-difference spectrum of compound **17** with selective saturation of one formyl proton (top spectrum).

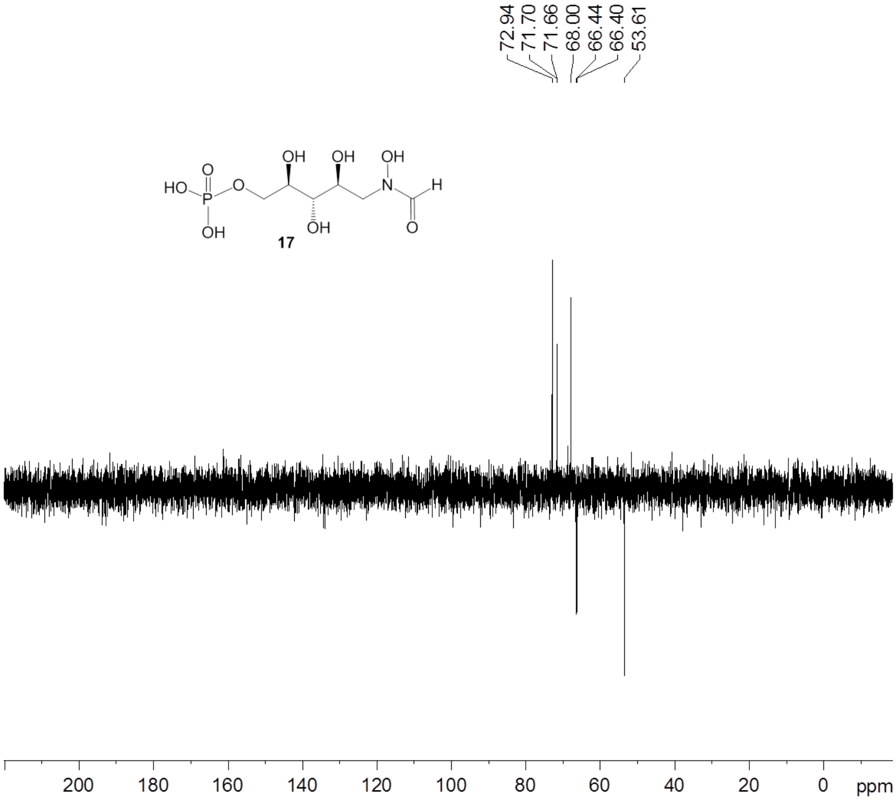
72.94
71.70
71.66
68.00
66.44
66.40
53.61
17
200
180
160
140
120
100
80
60
40
20
0
ppm

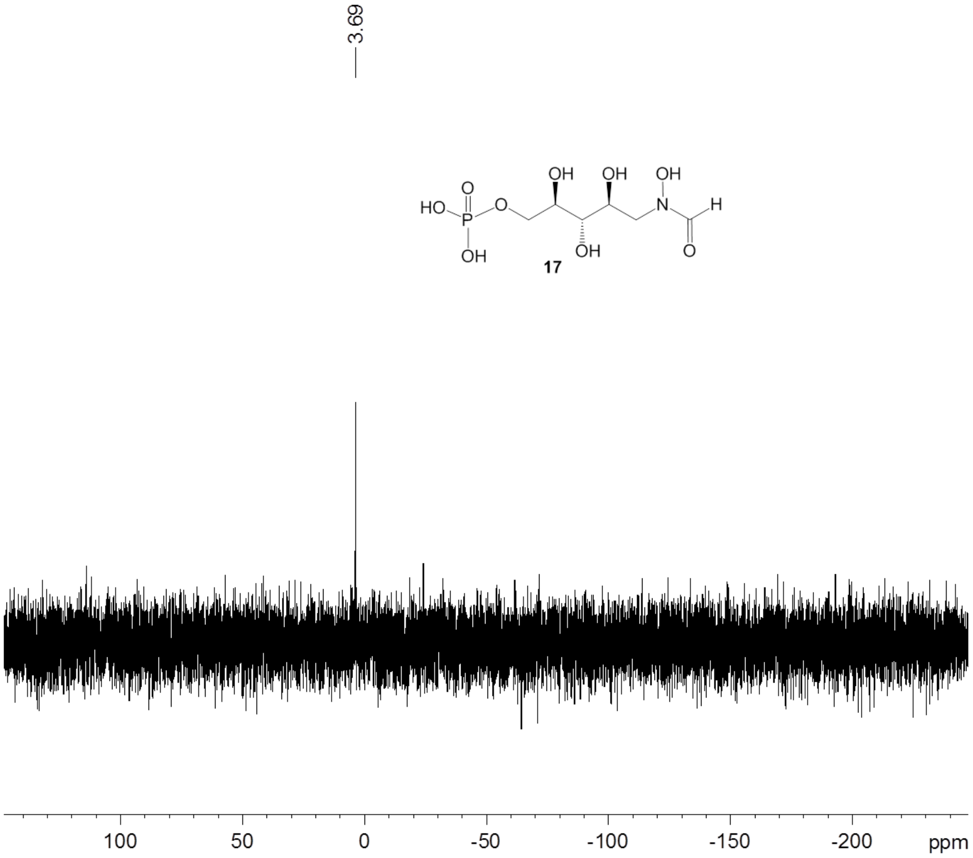
3.69
17
100
50
0
-50
-100
-150
-200
ppm

Fig

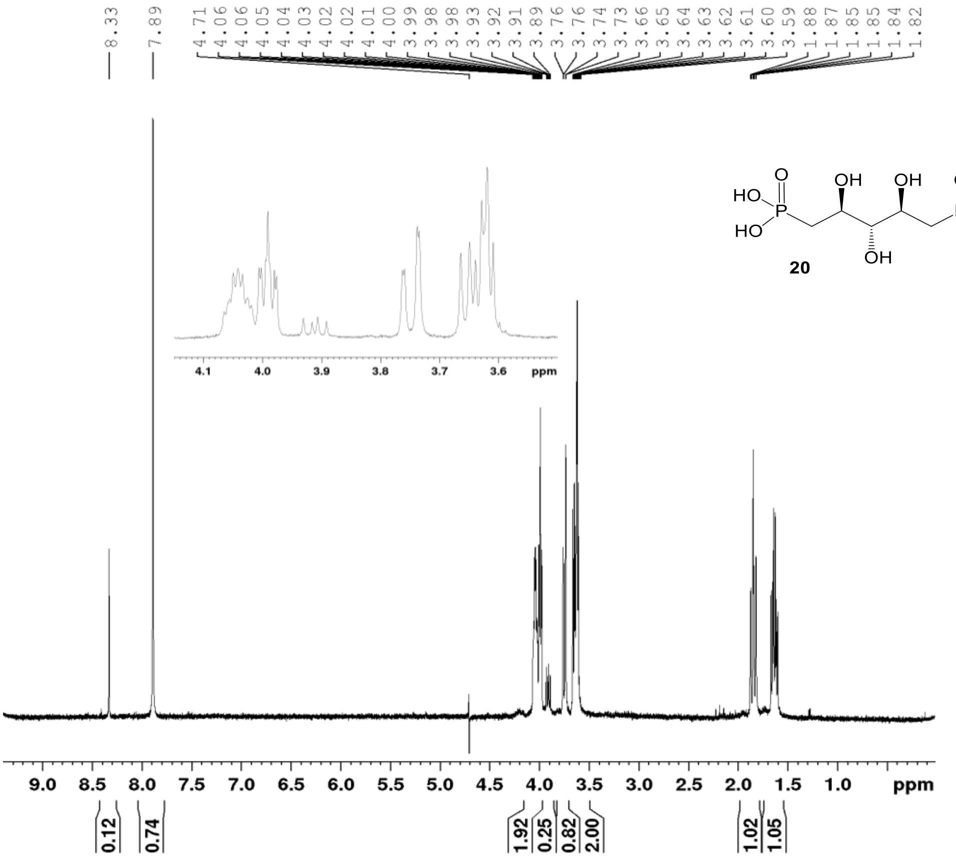
8.33
7.89
4.71
4.06
4.06
4.05
4.04
4.03
4.02
4.02
4.01
4.00
3.99
3.98
3.98
3.93
3.92
3.91
3.89
3.76
3.76
3.74
3.73
3.66
3.65
3.64
3.63
3.62
3.61
3.60
3.59
1.88
1.87
1.85
1.84
1.82
HO
HO
P
O
OH
OH
OH
OH
N
H
O
20
4.1 4.0 3.9 3.8 3.7 3.6 ppm
9.0 8.5 8.0 7.5 7.0 6.5 6.0 5.5 5.0 4.5 4.0 3.5 3.0 2.5 2.0 1.5 1.0 ppm
0.12
0.74
1.92
0.25
0.82
2.00
1.02
1.05

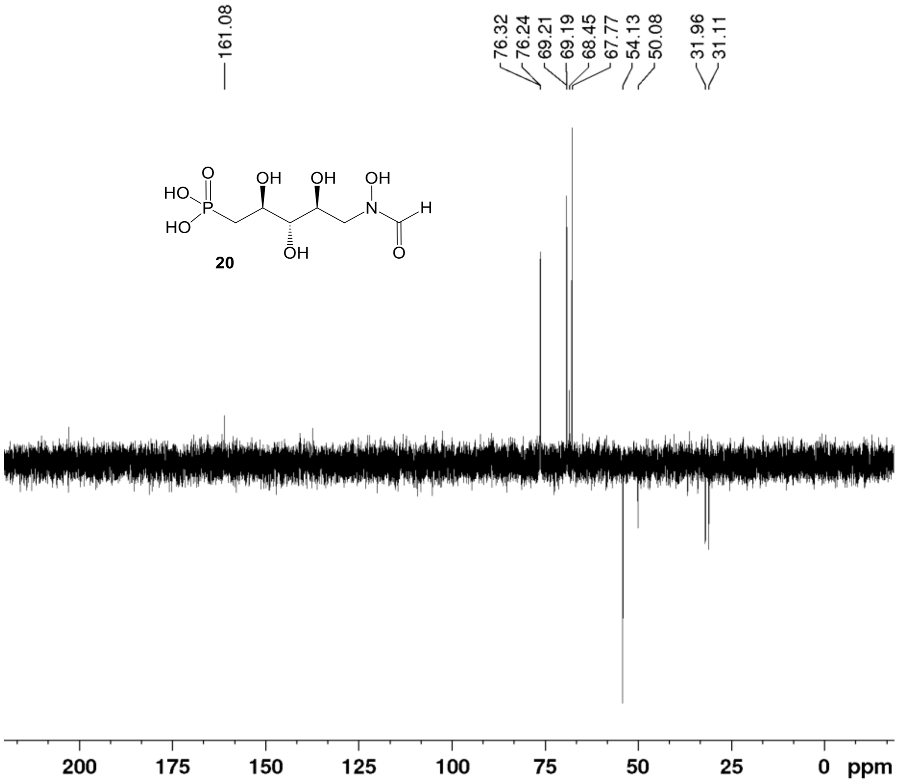
161.08
76.32
76.24
69.21
69.19
68.45
67.77
54.13
50.08
31.96
31.11
HO
HO
P
O
OH
OH
OH
OH
N
H
O
20
200 175 150 125 100 75 50 25 0 ppm

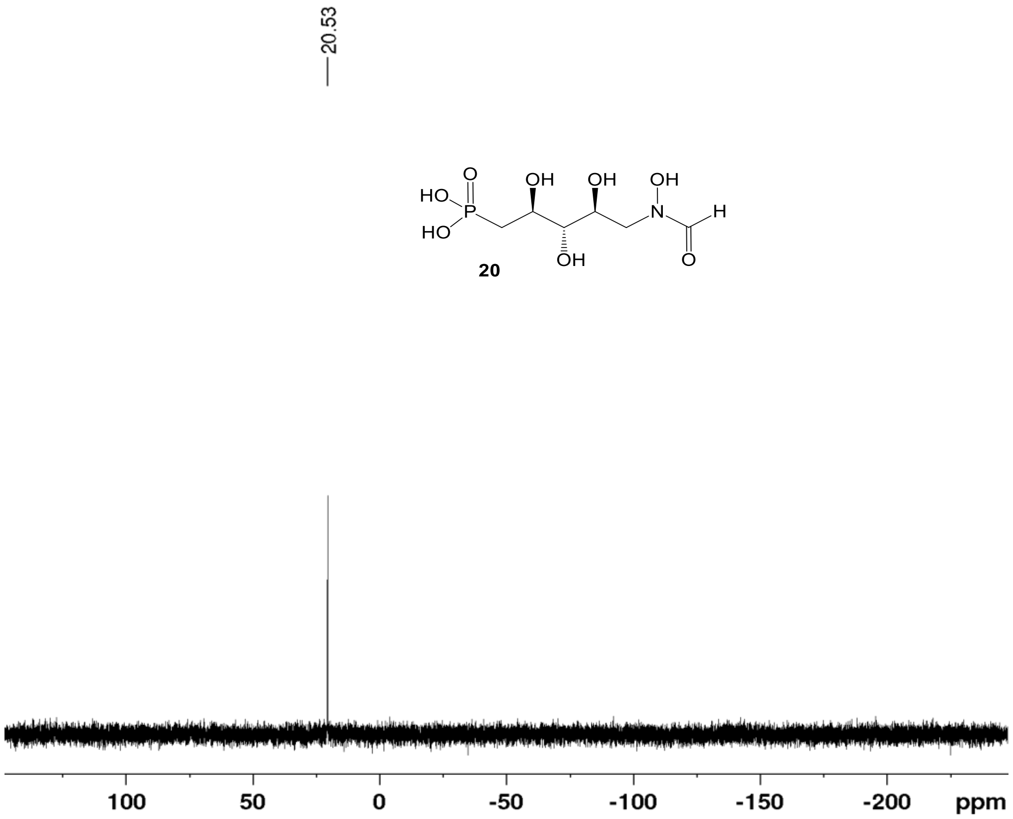
20.53
HO
HO
P
O
OH
OH
OH
OH
N
H
O
100
50
0
-50
-100
-150
-200
ppm

20

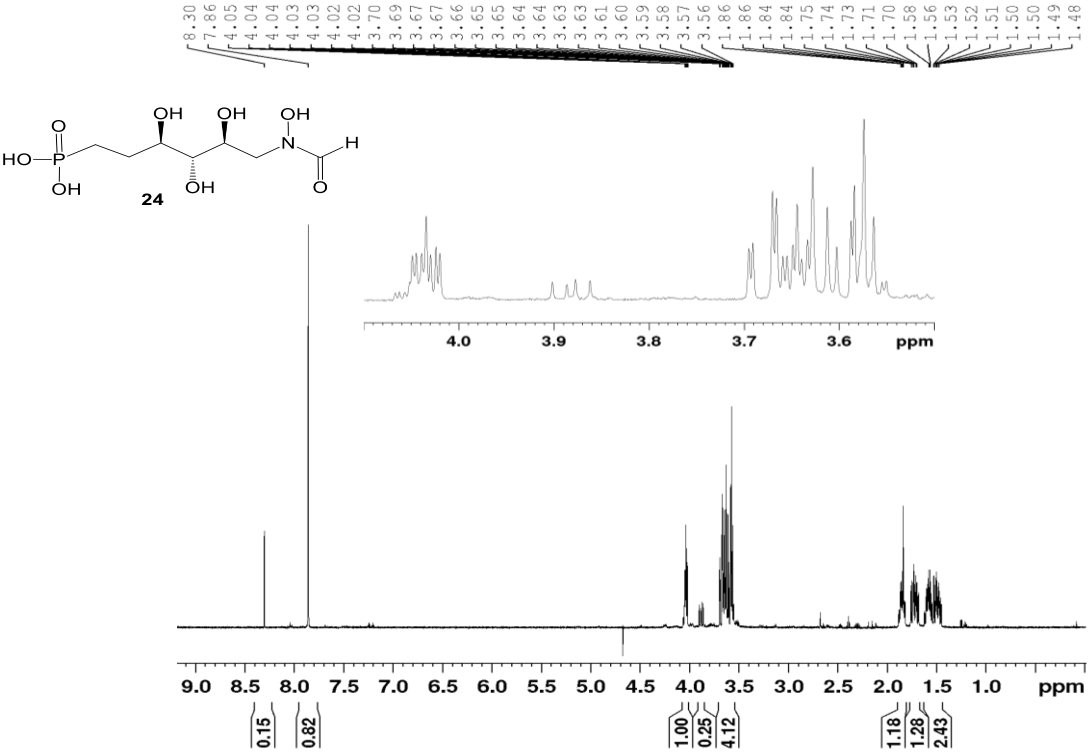
8.30
7.86
4.05
4.04
4.04
4.03
4.03
4.02
4.02
3.70
3.69
3.67
3.67
3.66
3.65
3.65
3.64
3.63
3.63
3.61
3.60
3.59
3.58
3.57
3.56
1.86
1.86
1.84
1.84
1.75
1.74
1.73
1.71
1.70
1.58
1.56
1.53
1.52
1.51
1.50
1.50
1.49
1.48
HO
P
O
OH
OH
OH
OH
OH
N
H
O
4.0
3.9
3.8
3.7
3.6
ppm
9.0
8.5
8.0
7.5
7.0
6.5
6.0
5.5
5.0
4.5
4.0
3.5
3.0
2.5
2.0
1.5
1.0
ppm
0.15
0.82
1.00
0.25
4.12
1.18
1.28
2.43

24

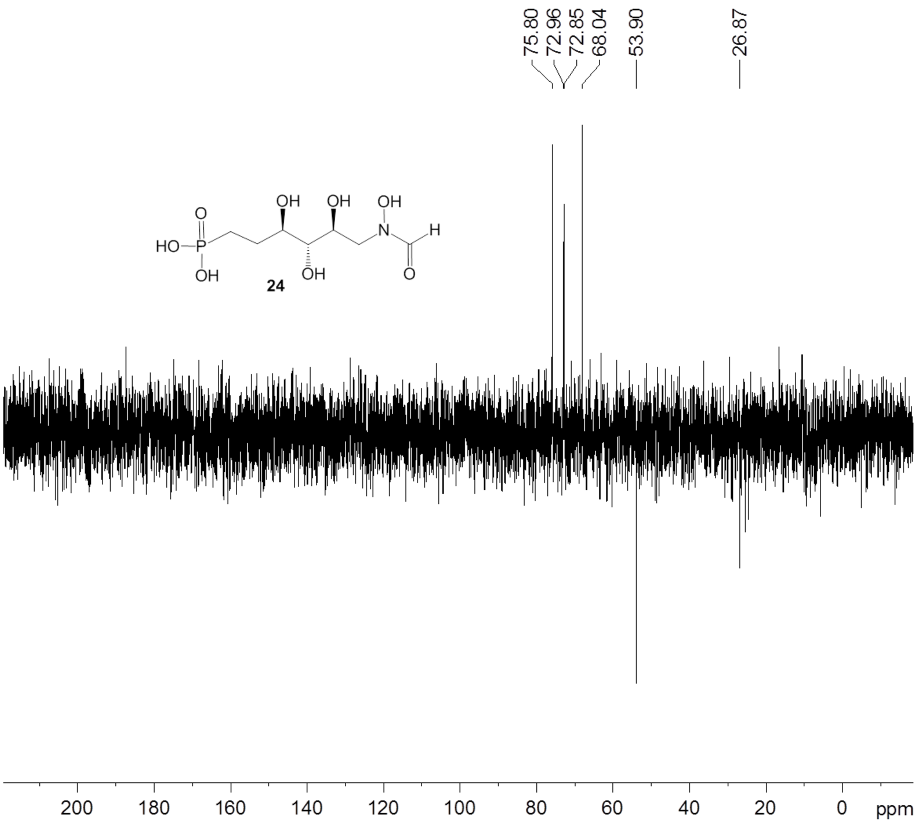
75.80
72.96
72.85
68.04
53.90
26.87
24
200 180 160 140 120 100 80 60 40 20 0 ppm

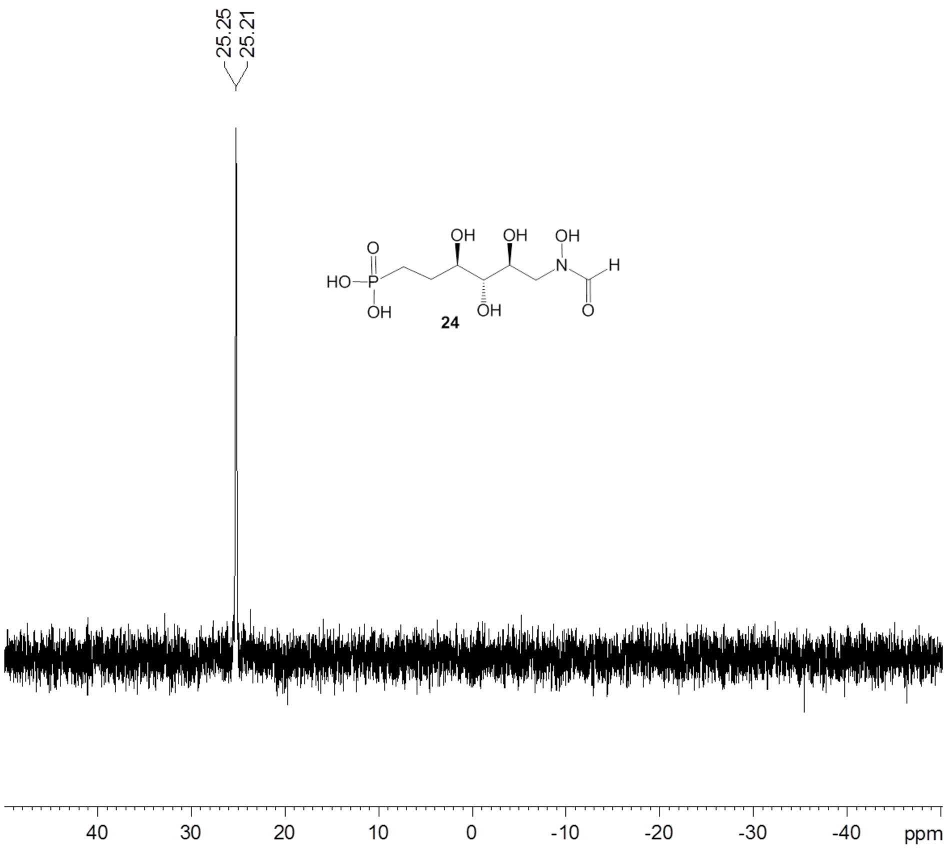
25.25
25.21
24
40 30 20 10 0 -10 -20 -30 -40 ppm

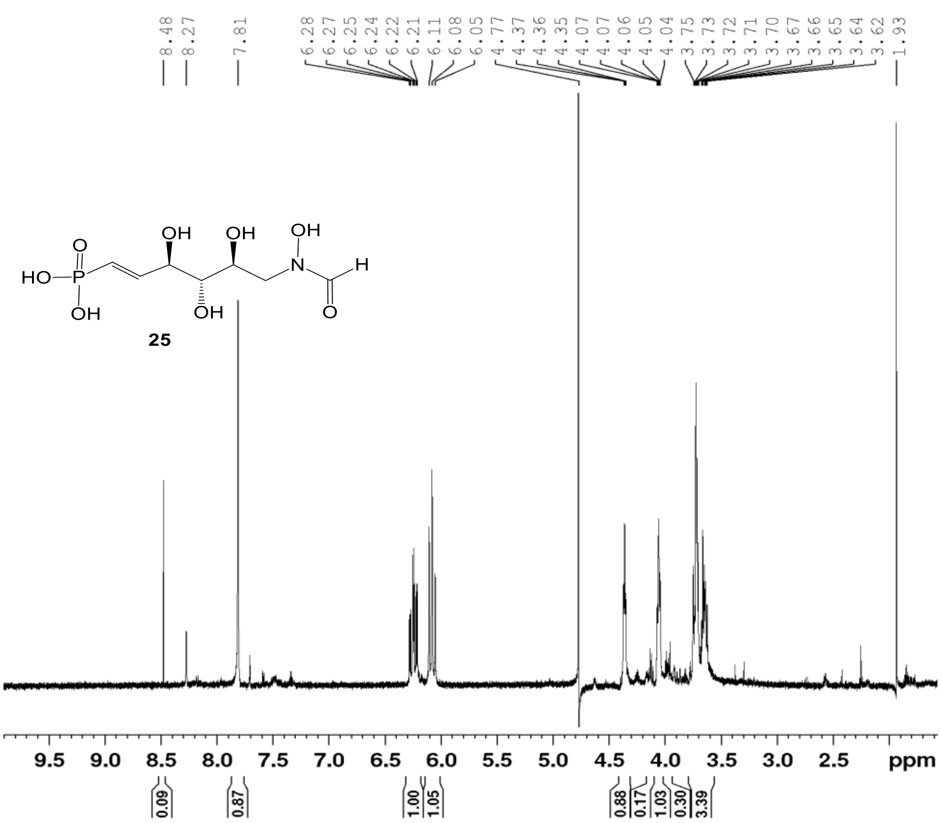
8.48
8.27
7.81
6.28
6.27
6.25
6.24
6.22
6.21
6.11
6.08
6.05
4.77
4.37
4.36
4.35
4.07
4.07
4.06
4.05
4.04
3.75
3.73
3.72
3.71
3.70
3.67
3.66
3.65
3.64
3.62
1.93
25
9.5 9.0 8.5 8.0 7.5 7.0 6.5 6.0 5.5 5.0 4.5 4.0 3.5 3.0 2.5 ppm
0.09
0.87
1.00
1.05
0.88
0.17
1.03
0.30
3.39

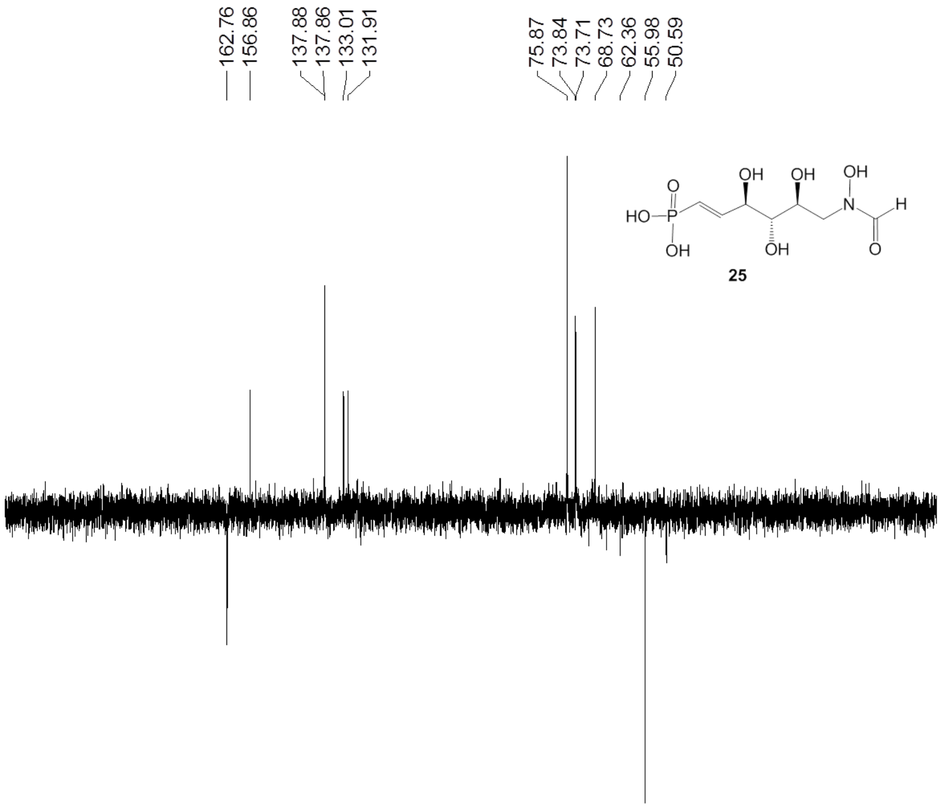
162.76
156.86
137.88
137.86
133.01
131.91
75.87
73.84
73.71
68.73
62.36
55.98
50.59
25
200 180 160 140 120 100 80 60 40 20 0 ppm

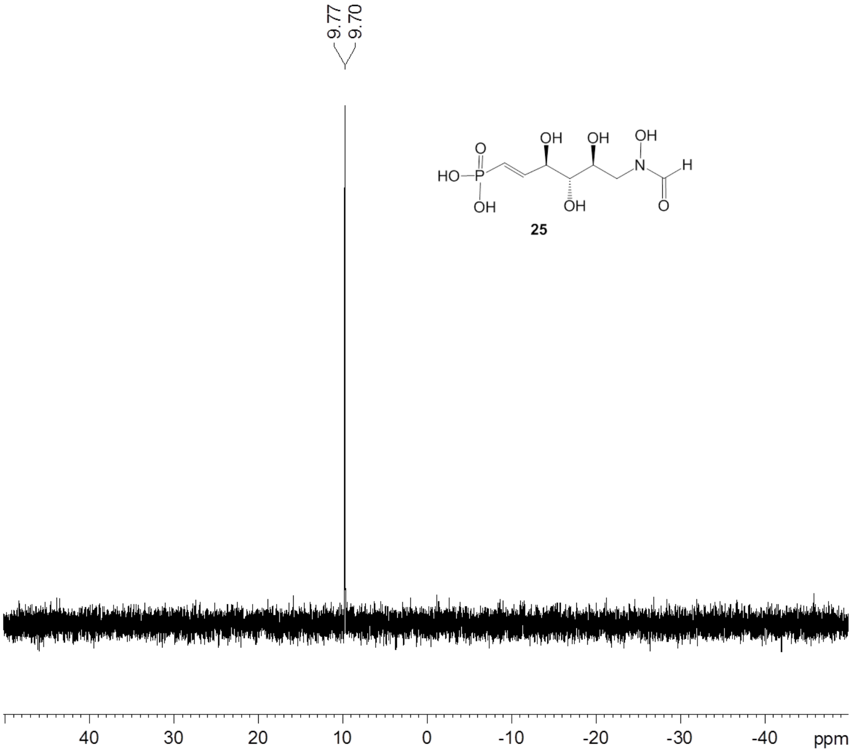
9.77
9.70
25
40
30
20
10
0
-10
-20
-30
-40
ppm

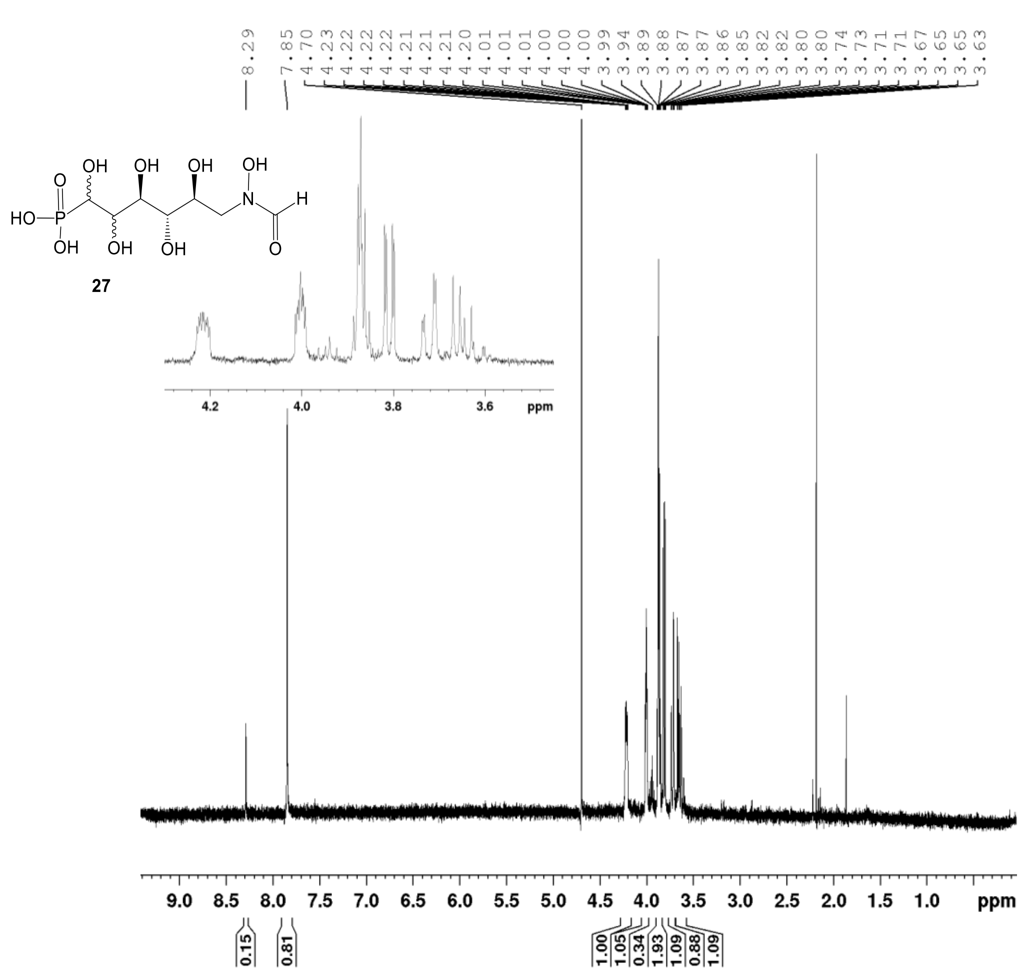
8.29
7.85
4.70
4.23
4.22
4.22
4.22
4.21
4.21
4.21
4.20
4.01
4.01
4.01
4.00
4.00
4.00
3.99
3.94
3.89
3.88
3.87
3.87
3.86
3.85
3.82
3.82
3.80
3.80
3.74
3.73
3.71
3.67
3.65
3.63
27
4.2
4.0
3.8
3.6
ppm
9.0
8.5
8.0
7.5
7.0
6.5
6.0
5.5
5.0
4.5
4.0
3.5
3.0
2.5
2.0
1.5
1.0
ppm
0.15
0.81
1.00
1.05
0.34
1.93
1.09
0.88
1.09

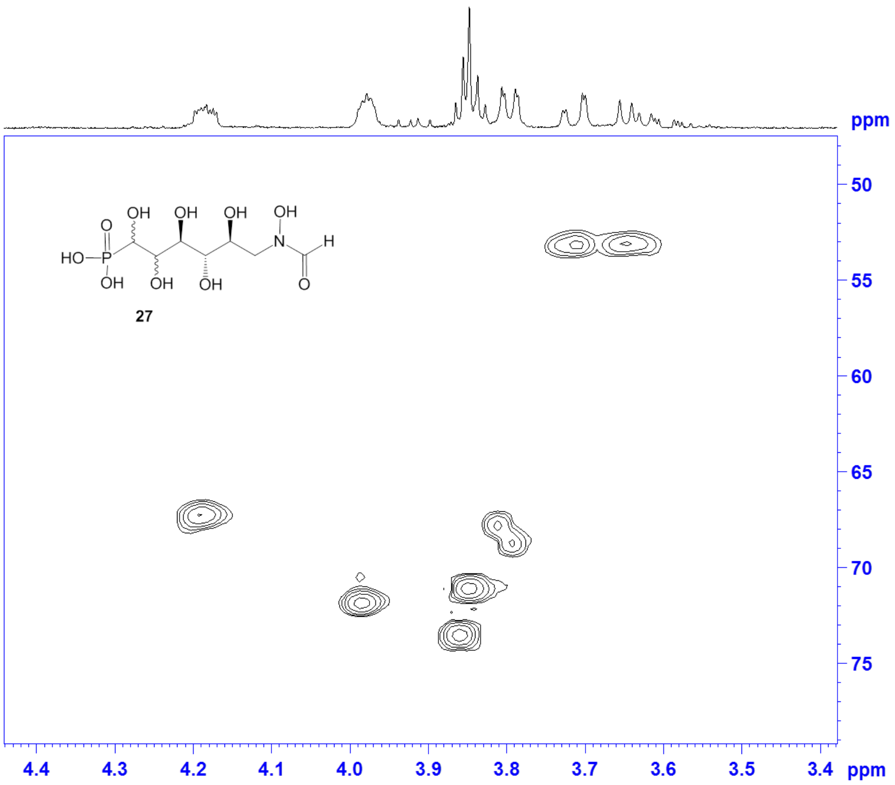

ppm
50
55
60
65
70
75
4.4
4.3
4.2
4.1
4.0
3.9
3.8
3.7
3.6
3.5
3.4
ppm
HO
P
O
OH
OH
OH
OH
OH
OH
OH
N
H
O
27

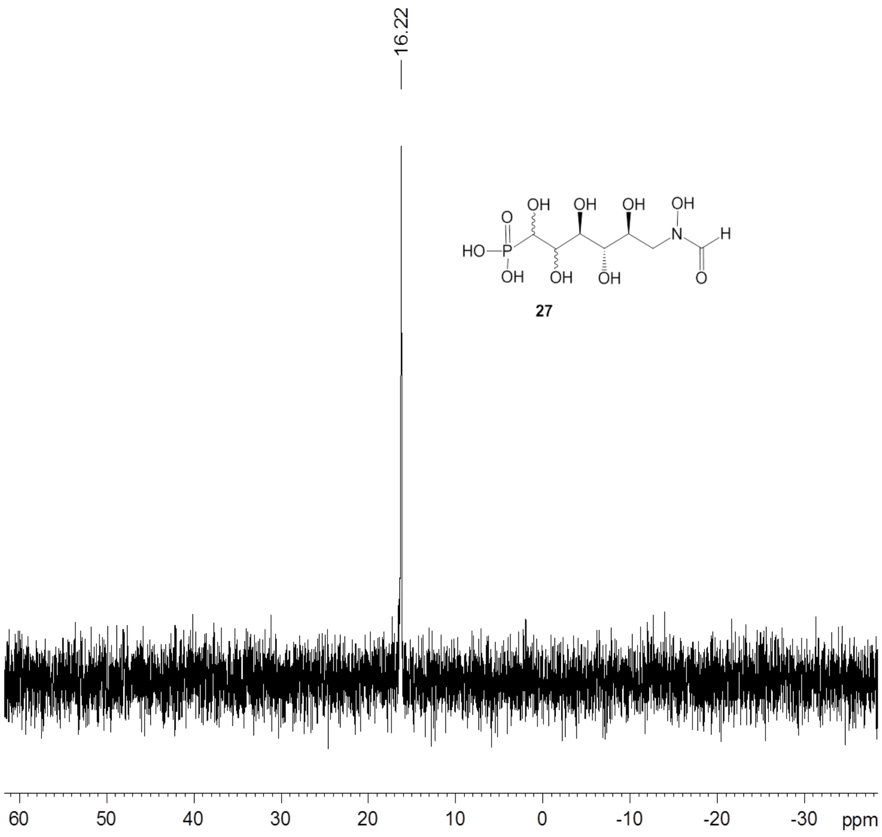

16.22
HO
P
O
OH
OH
OH
OH
OH
OH
OH
N
H
O
27
60
50
40
30
20
10
0
-10
-20
-30
ppm

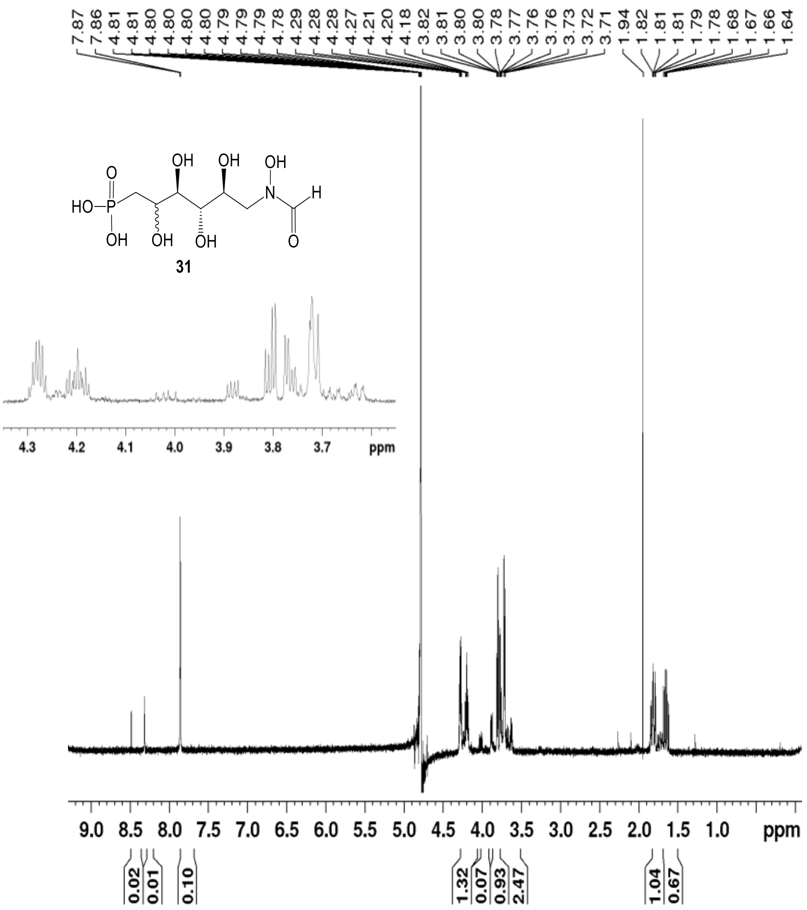
7.87 7.86 4.81 4.81 4.80 4.80 4.80 4.80 4.79 4.79 4.79 4.78 4.29 4.28 4.28 4.27 4.21 4.20 4.18 3.82 3.81 3.80 3.80 3.78 3.77 3.76 3.76 3.73 3.72 3.71 1.94 1.82 1.81 1.81 1.79 1.78 1.68 1.67 1.66 1.64
O
HO–P
OH
OH OH OH OH OH
N
H
O
31
4.3 4.2 4.1 4.0 3.9 3.8 3.7 ppm
9.0 8.5 8.0 7.5 7.0 6.5 6.0 5.5 5.0 4.5 4.0 3.5 3.0 2.5 2.0 1.5 1.0 ppm
0.02 0.01 0.10 1.32 0.07 0.93 2.47 1.04 0.67

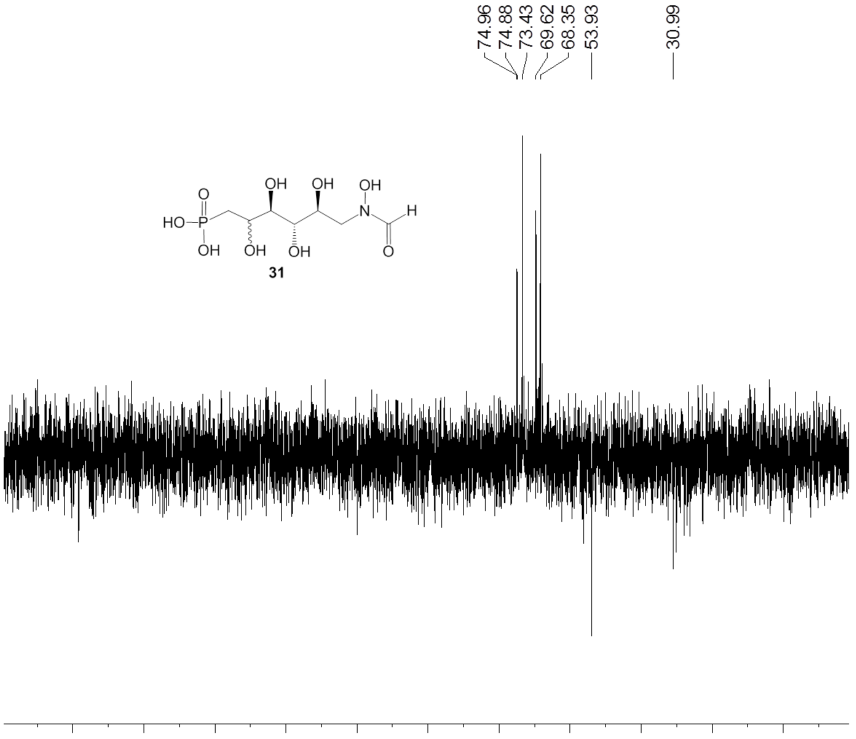
74.96 74.88 73.43 69.62 68.35 53.93 30.99
31
200 180 160 140 120 100 80 60 40 20 0 ppm

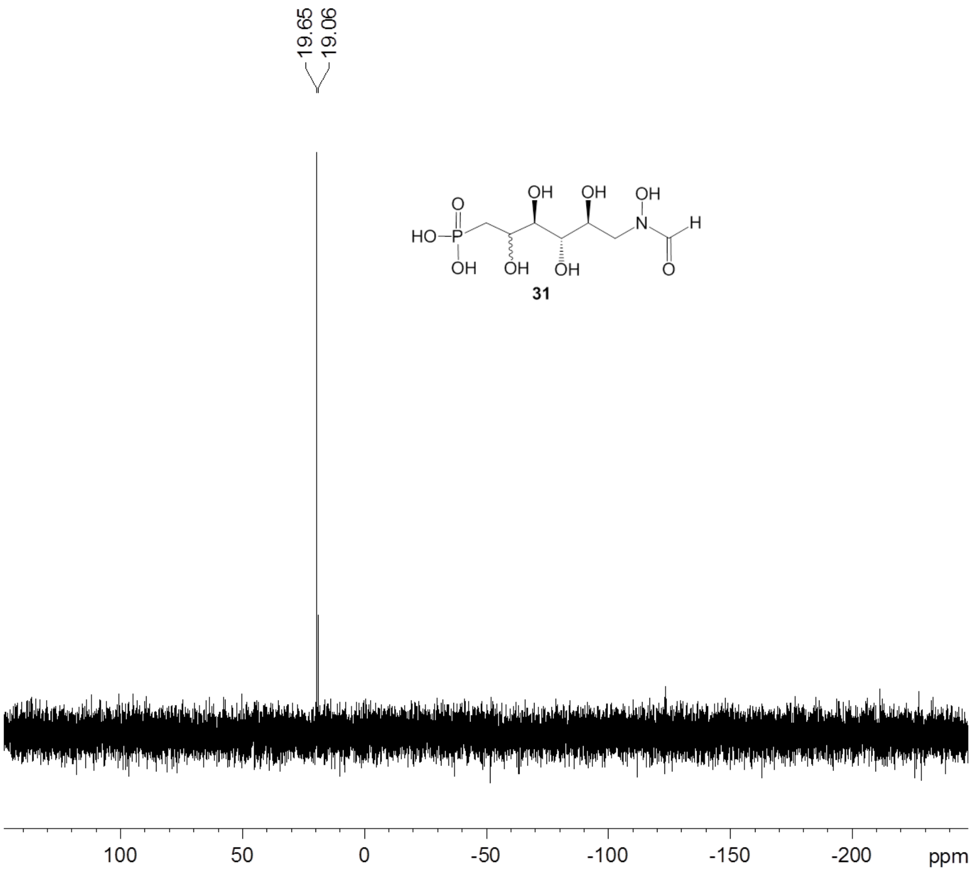
19.65
19.06
HO
P
O
OH
OH
OH
OH
OH
OH
N
H
O
31
100
50
0
-50
-100
-150
-200
ppm

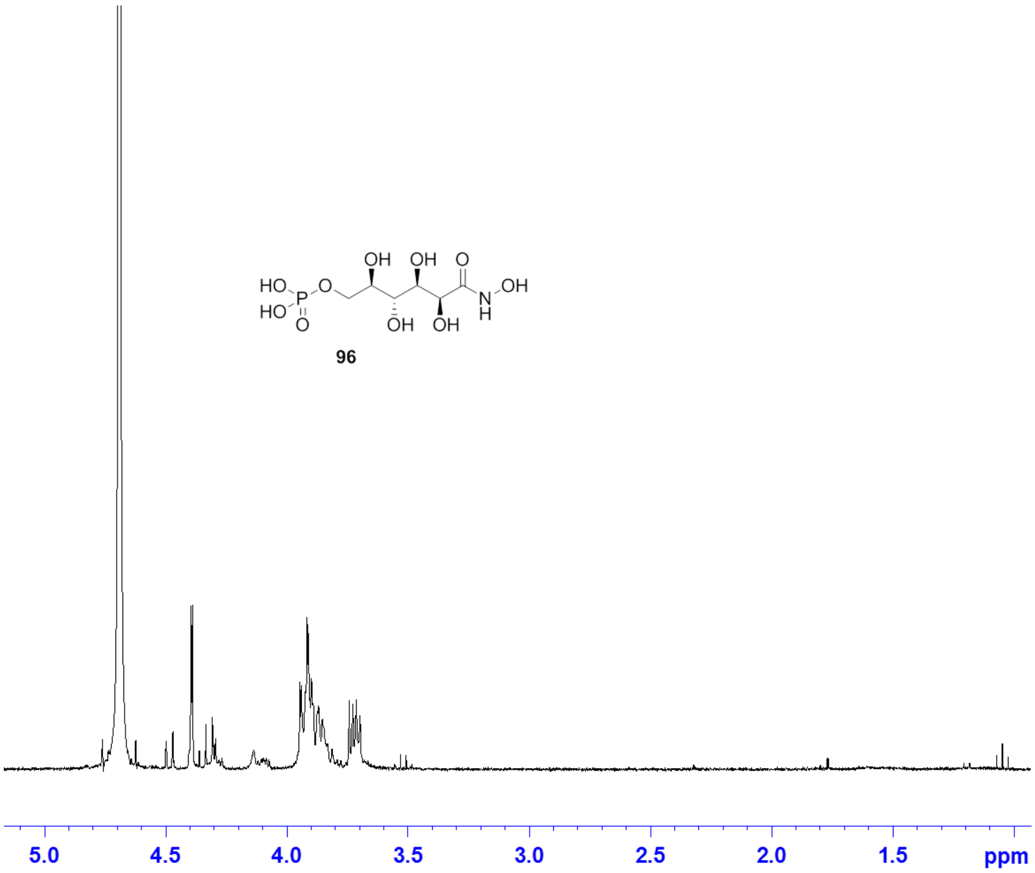
HO
HO
P
O
O
OH
OH
O
OH
OH
N
H
OH
96
5.0
4.5
4.0
3.5
3.0
2.5
2.0
1.5
ppm

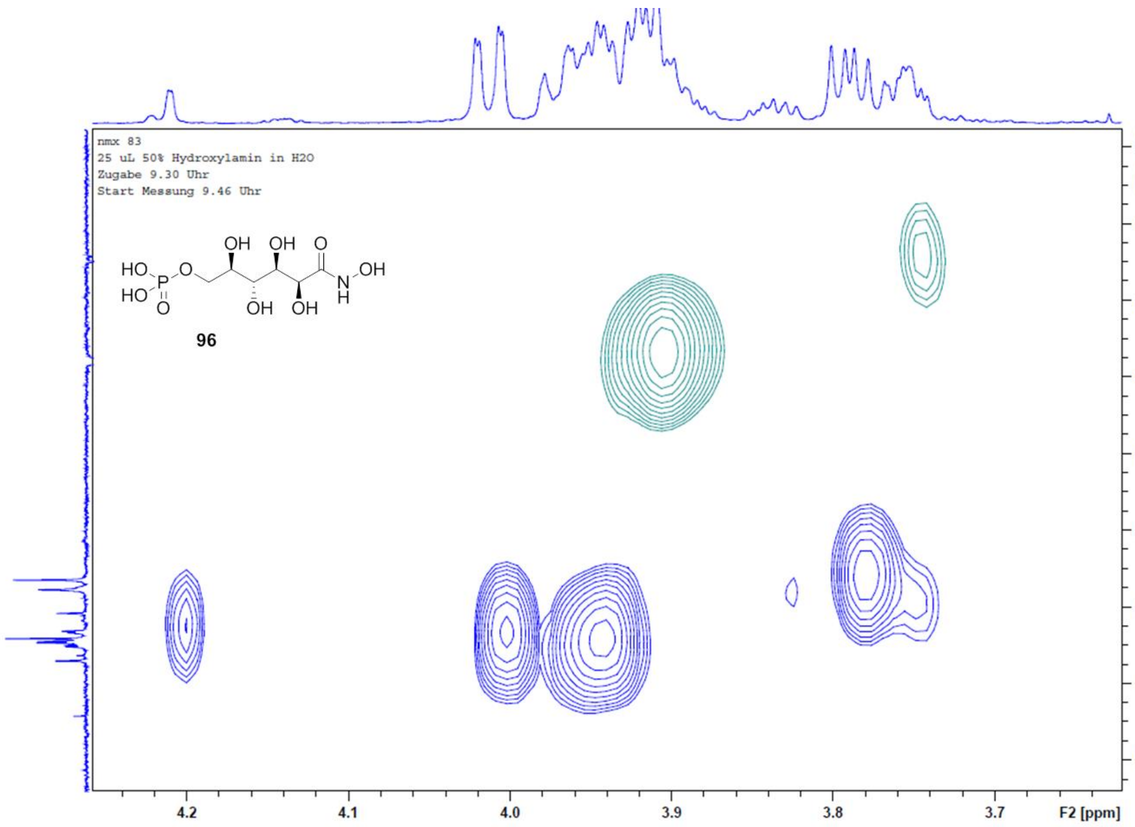
nmx 83
25 uL 50% Hydroxylamin in H2O
Zugabe 9.30 Uhr
Start Messung 9.46 Uhr
96
F1 [ppm]
F2 [ppm]

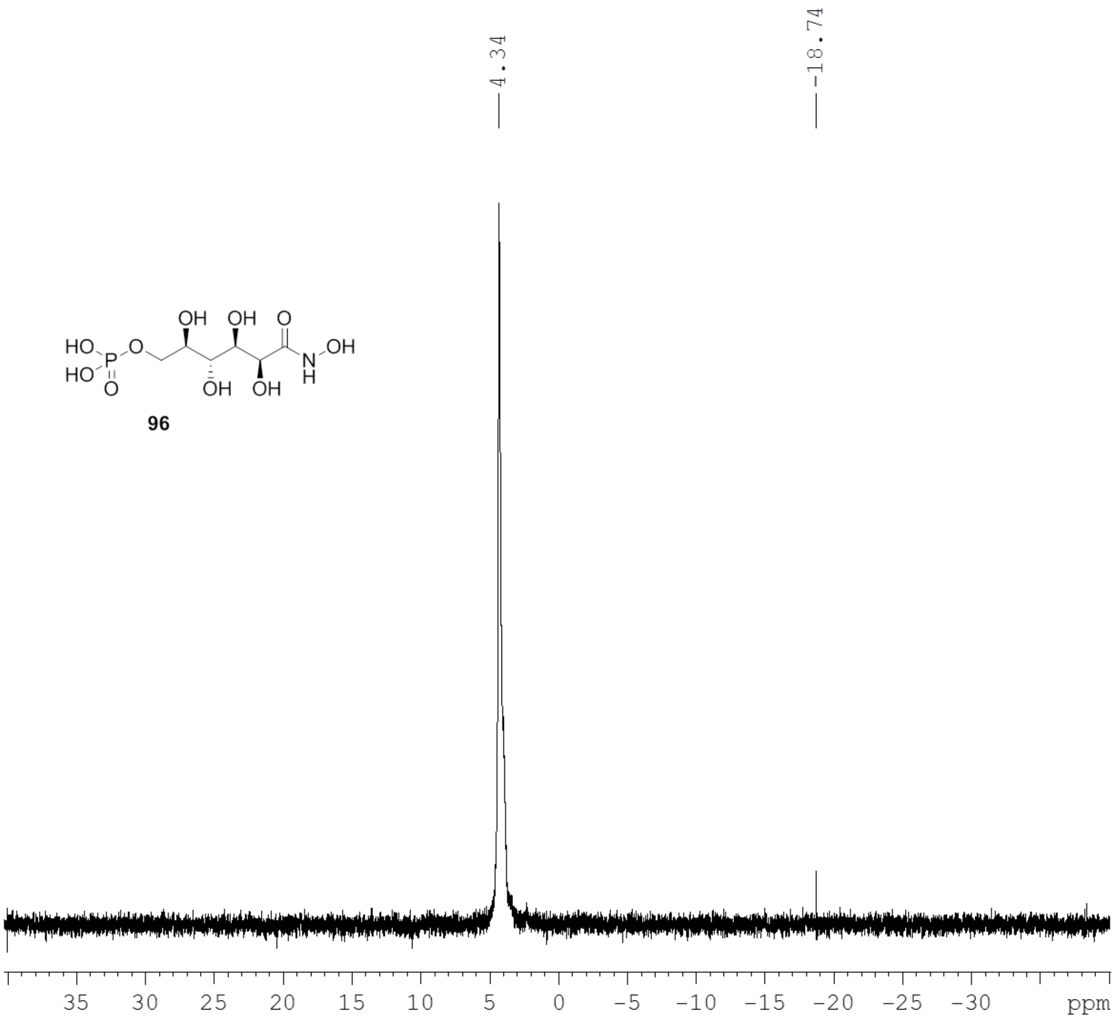
4.34
-18.74
96
ppm

The synthetic procedures for compounds **41**, **54**, **68**, **76** and **84** are fully described in the published patent WO 2014/067904 A1.

Compound **41** is described in example 4.

Compound **54** is described in example 11.

Compound **68** is described in example 15.

Compound **76** is described in example 9.

Compound **84** is described in example 12.

Table S1. X-Ray data collection and processing statistics.

PDB Accession Code	**8V4J**	**8V2T**
Inhibitor	**17**	**84**
Wavelength (Å)	1,075	1,100
Resolution range (Å)	36.83 - 1.31 (1.357 - 1.31)	39.06 - 1.402 (1.452 - 1.402)
Space group	P 42 21 2	P 42 21 2
Cell dimensions: a, b, c (Å)	60.972 60.972 92.429	60.906 60.906 92.728
Cell dimensions: α, β, γ (°)	90 90 90	90 90 90
Total reflections	582507 (57379)	417025 (33367)
Unique reflections	42503 (4190)	34849 (3323)
Multiplicity	13.7 (13.7)	12.0 (10.0)
Completeness (%)	99.68 (100.00)	99.64 (96.54)
Mean I/sigma(I)	41.13 (8.89)	21.23 (3.22)
Wilson B-factor	10.62	13.42
R-merge	0.03807 (0.2714)	0.06467 (0.4377)
R-meas	0.03959 (0.2819)	0.06761 (0.4607)
R-pim	0.01072 (0.07575)	0.01944 (0.1396)
CC1/2	1 (0.985)	0.999 (0.946)
CC*	1 (0.996)	1 (0.986)
Reflections used in refinement	42502 (4190)	34846 (3320)
Reflections used for R-free	2125 (212)	1745 (171)
R-work	0.1607 (0.1950)	0.1568 (0.2528)
R-free	0.1725 (0.2104)	0.1633 (0.2526)
CC(work)	0.966 (0.917)	0.966 (0.896)
CC(free)	0.959 (0.901)	0.927 (0.886)
Number of non-hydrogen atoms	1698	1647
macromolecules	1424	1407
ligands	20	22
solvent	254	218
Protein residues	192	191
RMS bond length deviation (Å)	0.01	0.008
RMS bond angle deviation (°)	1.03	0.85
Ramachandran favored (%)	98.42	98.41
Ramachandran allowed (%)	1.58	1.59
Ramachandran outliers (%)	0	0
Rotamer outliers (%)	0.68	0.69
Clashscore	0,69	1,06
Average B-factor	15.14	16.5
macromolecules	12.87	14.76
ligands	12.42	17.04
solvent	28.1	27.69

Statistics for the highest-resolution shell are shown in parentheses.

Figure S3: Role of UhpT phospho-sugar transporter in the transport of D-*glycero*-D-*manno*-heptose 7-phosphate (H7P). Complementation of *E. coli* C7-*gmhA*- by the GmhA product H7P. Silver-stained LPS SDS-PAGE of *E. coli* C7 WT (lane 1) or *gmhA*- (lanes 2-4). Culture in presence of 300 µM H7P (lane 3) restores some full length LPS, showing a limited entry of H7P into the cells. UhpT induction by 100 µM glucose-6-phosphate (G6P) allows a strong transport of H7P, as evidenced by the restoration of a full LPS profile (lane 4).

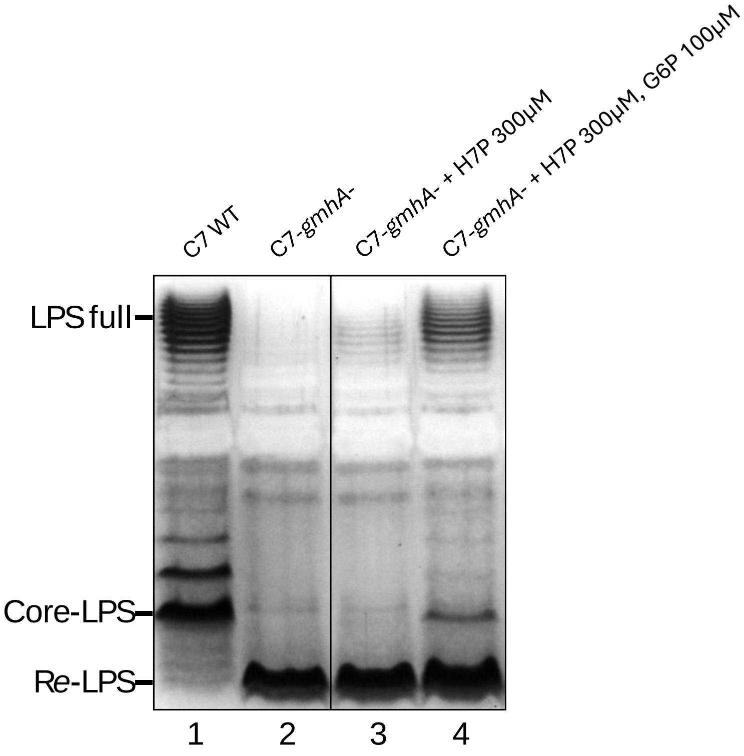

Figure S4: IC_{50} fits of *E. coli* GmhA in biochemical inhibition assays. Red dashed lines show the fit IC_{50} values. A: compounds **17** to **25**; B: compounds **27** to **54**; C: compounds **68** to **96**.

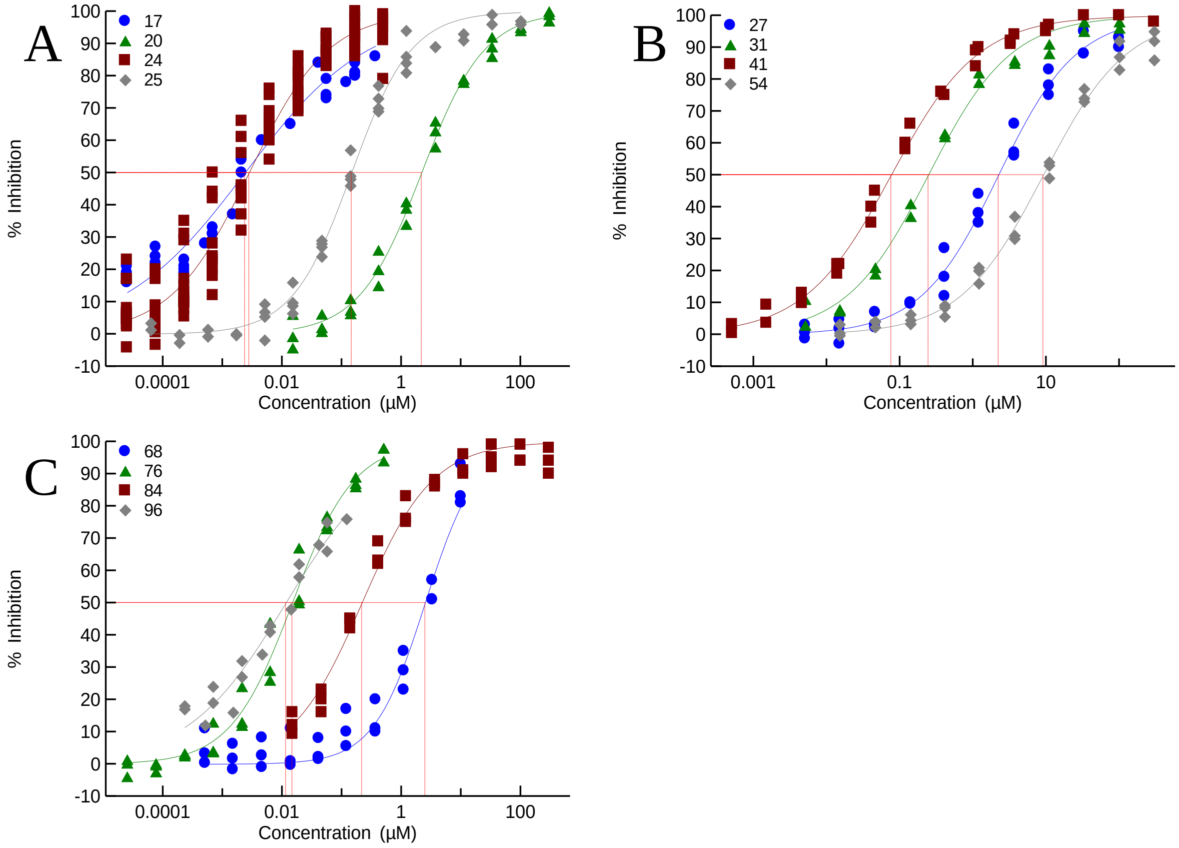

Figure S5: EC_{50} fits of LPS biosynthesis inhibition. Red dashed lines show the fit EC_{50} values. A: compounds **17** to **25**; B: compounds **27** to **54**; C: compounds **68** to **96**.

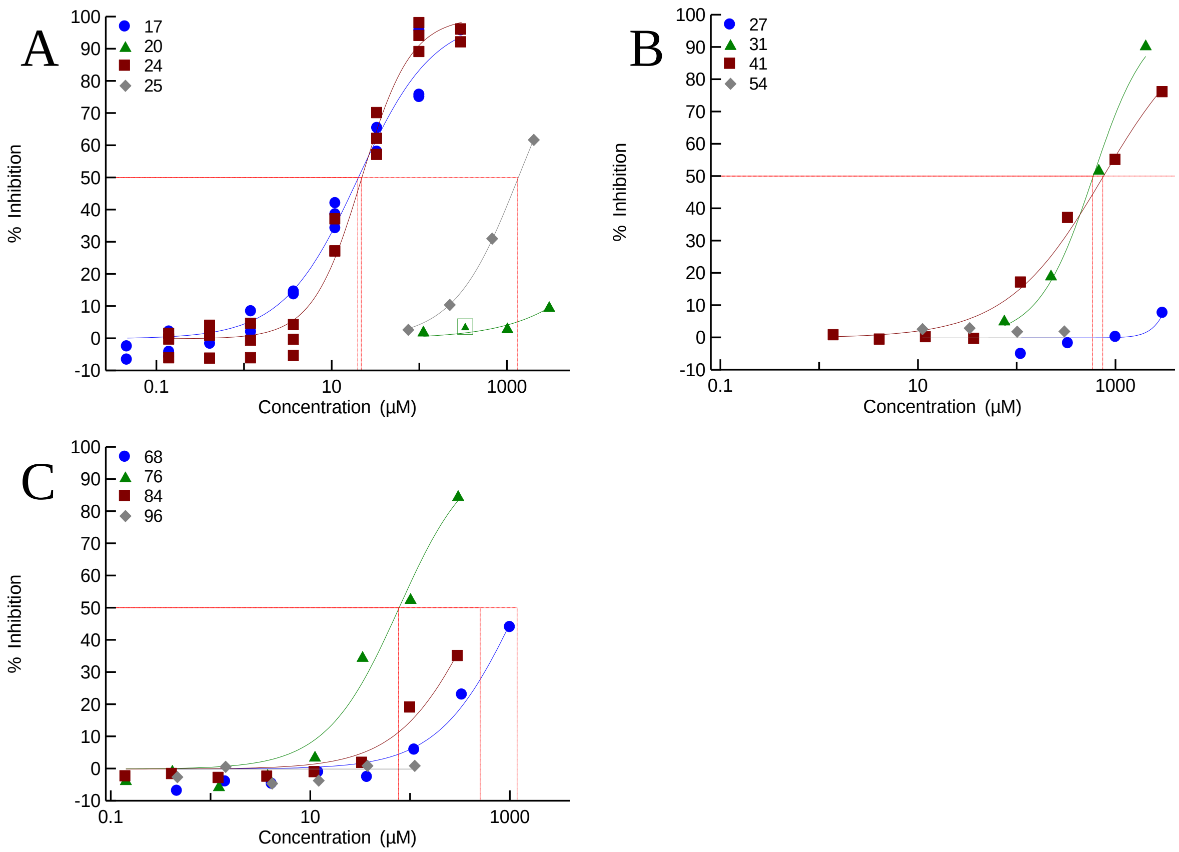

Supplement: Supplementary file 1 — jm4c00037_si_001.pdf [file jm4c00037_si_001.pdf]
